# Enabling synthesis in fragment-based drug discovery by reactivity mapping: photoredox-mediated cross-dehydrogenative heteroarylation of cyclic amines†
†Electronic supplementary information (ESI) available. See DOI: 10.1039/c8sc04789h


**DOI:** 10.1039/c8sc04789h

**Published:** 2018-12-21

**Authors:** Rachel Grainger, Tom D. Heightman, Steven V. Ley, Fabio Lima, Christopher N. Johnson

**Affiliations:** a Astex Pharmaceuticals , 436 Cambridge Science Park, Milton Road , Cambridge , CB4 0QA , UK . Email: rachel.grainger@astx.com ; Email: chris.johnson@astx.com; b Department of Chemistry , University of Cambridge , Lensfield Road , Cambridge CB2 1EW , UK; c Novartis Pharma AG , Novartis Campus , 4002 Basel , Switzerland

## Abstract

In fragment-based drug discovery (FBDD), a weakly binding fragment hit is elaborated into a potent ligand by bespoke functionalization along specific directions (growth vectors) from the fragment core in order to complement the 3D structure of the target protein. This structure-based design approach can present significant synthetic challenges, as growth vectors often originate on sp^2^ or sp^3^ ring carbons which are not the most synthetically accessible points on the fragment. To address this issue and expedite synthesis in FBDD, we established a nanogram-to-gram workflow for the development of enabling synthetic transformations, such as the direct C–H functionalization of heterocycles. This novel approach deploys high-throughput experimentation (HTE) in 1536-well microtiter plates (MTPs) facilitated by liquid handling robots to screen reaction conditions on the nanomolar scale; subsequently the reaction is upscaled in a continuous flow to generate gram-quantities of the material. In this paper, we disclose the use of this powerful workflow for the development of a photoredox-mediated cross-dehydrogenative coupling of fragments and medicinally relevant heterocyclic precursors *via* Minisci-type addition of α-amino radicals to electron-deficient heteroarenes. The optimized reaction conditions were employed on the milligram-scale on a diverse set of 112 substrates to map out structure–reactivity relationships (SRRs) of the transformation. The coupling exhibits excellent tolerance to a variety of functional groups and N-rich heteroarenes relevant to FBDD and was upscaled in a continuous flow to afford gram-quantities of pharmaceutically relevant sp^2^–sp^3^ privileged architectures.

## Abstract 2

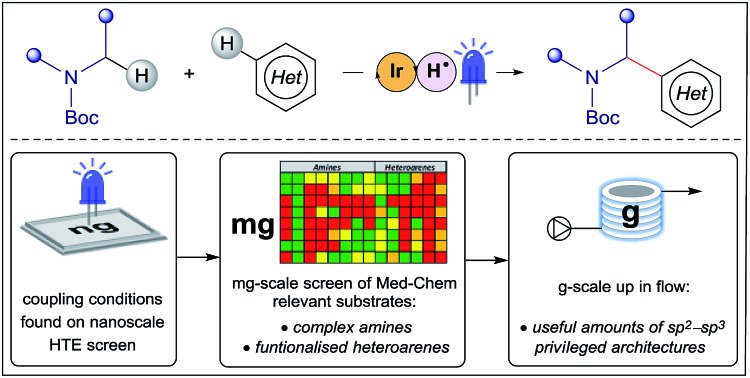
A nanogram-to-gram workflow has been established for the identification and development of synthetic transformations which are enabling in Fragment-Based Drug Discovery (FBDD). In this study, we disclose a method for the synthesis of privileged sp^2^–sp^3^ architectures *via* direct cross-dehydrogenative coupling of heterocycles.

## Introduction

### Increasing target affinity & selectivity with structure-based design

**Fig. 1:**
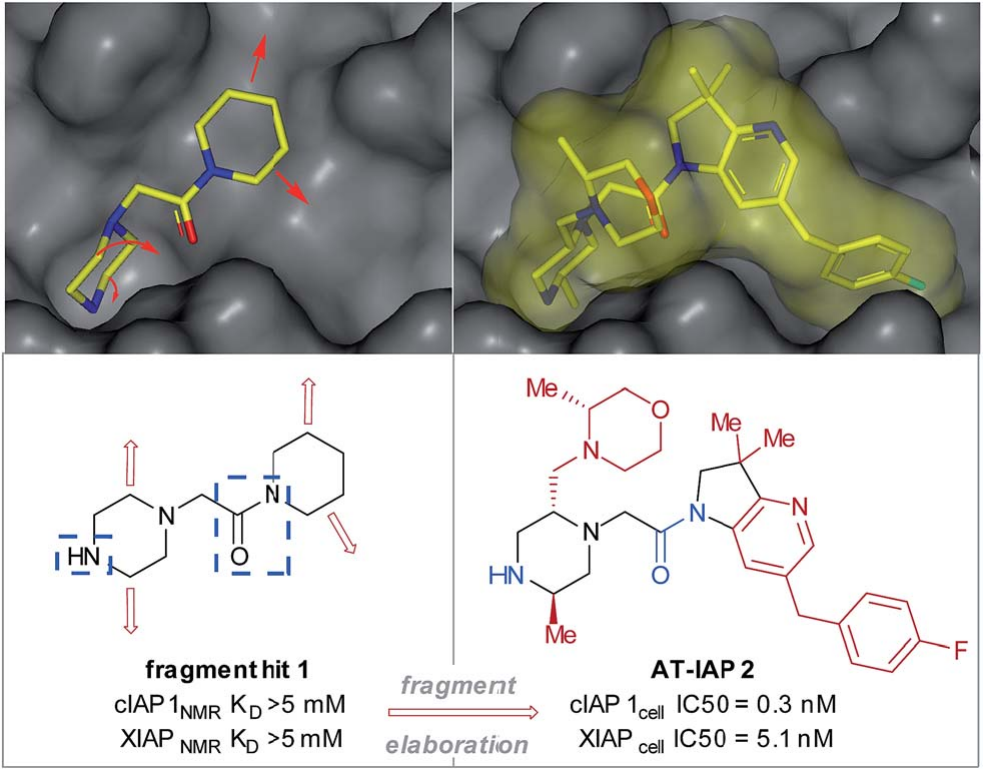
Fragment hit 1 was found to bind to the apoptosis proteins cIAP1 and XIAP (>5 mM affinity). The key interacting motifs (the minimal pharmacophore) are highlighted in blue. Using structure-based design, this fragment hit was elaborated along specific growth vectors (highlighted in red) to fill the binding pockets of the two proteins, resulting in nanomolar lead compound **2**, with ∼15-fold selectivity for cIAP1 *vs.* XIAP, as determined by cellular assays.^8^–^10^

Fragment-based drug discovery (FBDD) is an established medicinal chemistry approach to generate leads for protein targets.^1^–^3^ To date, this has resulted in two approved drugs (venetoclax and vemurafenib) and numerous FBDD-derived candidates in clinical trials.^1^ The FBDD approach involves screening low molecular weight (140–200 Da, clog *P* 0–2) polar compounds (*e.g.* fragment hit 1, Fig. 1), that bind to biological macromolecules with a high ligand efficiency (LE).^4^ After hit identification,^5^ high resolution X-ray crystallographic structural data drive the subsequent fragment-to-lead (F2L) optimization process, during which the fragment hit is functionalized in specific directions (growth vectors) to generate a high-affinity lead compound that forms favorable H-bonding interactions with the residues on the protein in addition to having good 3D shape complementarity with the pocket through lipophilic interactions (Fig. 1).^6^,^7^ During the design process, the growth vectors that are identified on the fragment as points for synthetic elaboration are mandated by the protein architecture and often do not correspond to those which are the most synthetically accessible points on the molecule. As a result, fragment analogues containing functional handles are needed during the F2L stage to ensure that the desired growth vector elaboration is achieved. However, the commercial availability of pre-functionalized and/or protected fragments may be limited, and the resulting additional synthetic steps cumulatively increase the time taken to identify the lead and subsequent clinical candidate. For medicinal chemists, there is often a trade-off between the perceived value of a proposed target molecule (reflecting the strength of the design rationale) and the ability to devise an efficient synthetic route, to the extent that high-quality designs are not acted upon due to the constraints of the synthetic methodology and raw material availability. A long-term vision is to remove such synthetic constraints and greatly expand the network of available molecules related to a given fragment.^11^,^12^ Accordingly, use of C–H functionalization to directly elaborate unfunctionalized growth vectors on the native fragment would allow rapid investigation of structure–activity relationships (SARs) and accelerate the F2L process.

### Aliphatic heterocycles in FBDD

Piperazines, morpholines and other saturated N-heterocycles are architectures that are not only prevalent in FBDD but are ubiquitous throughout medicinal chemistry.^13^,^14^ Selective elaboration of these architectures and related ring-systems is not trivial; due to the strength of the C–H bond, strongly basic or reactive reagents are often required resulting in reaction conditions which are non-selective and incompatible with sensitive functional groups.^15^–^18^ During a recent FBDD campaign to target the inhibitor of apoptosis proteins (IAPs), fragment 1 which contains acetyl-linked piperidine and piperazine rings was found to bind the proteins cIAP1 and XIAP (Fig. 1).^8^–^10^ Modification of this initial fragment hit (1) along the C(sp^3^)–H growth vectors resulted in a potent elaborated lead compound, AT-IAP (2). However, during the F2L process, methods for direct functionalization of the native fragment (and related intermediates) were not available. Consequently, at all stages of the F2L process, the heterocyclic cores had to be constructed individually using multistep routes, with the desired motifs already pre-installed.

**Fig. 2:**
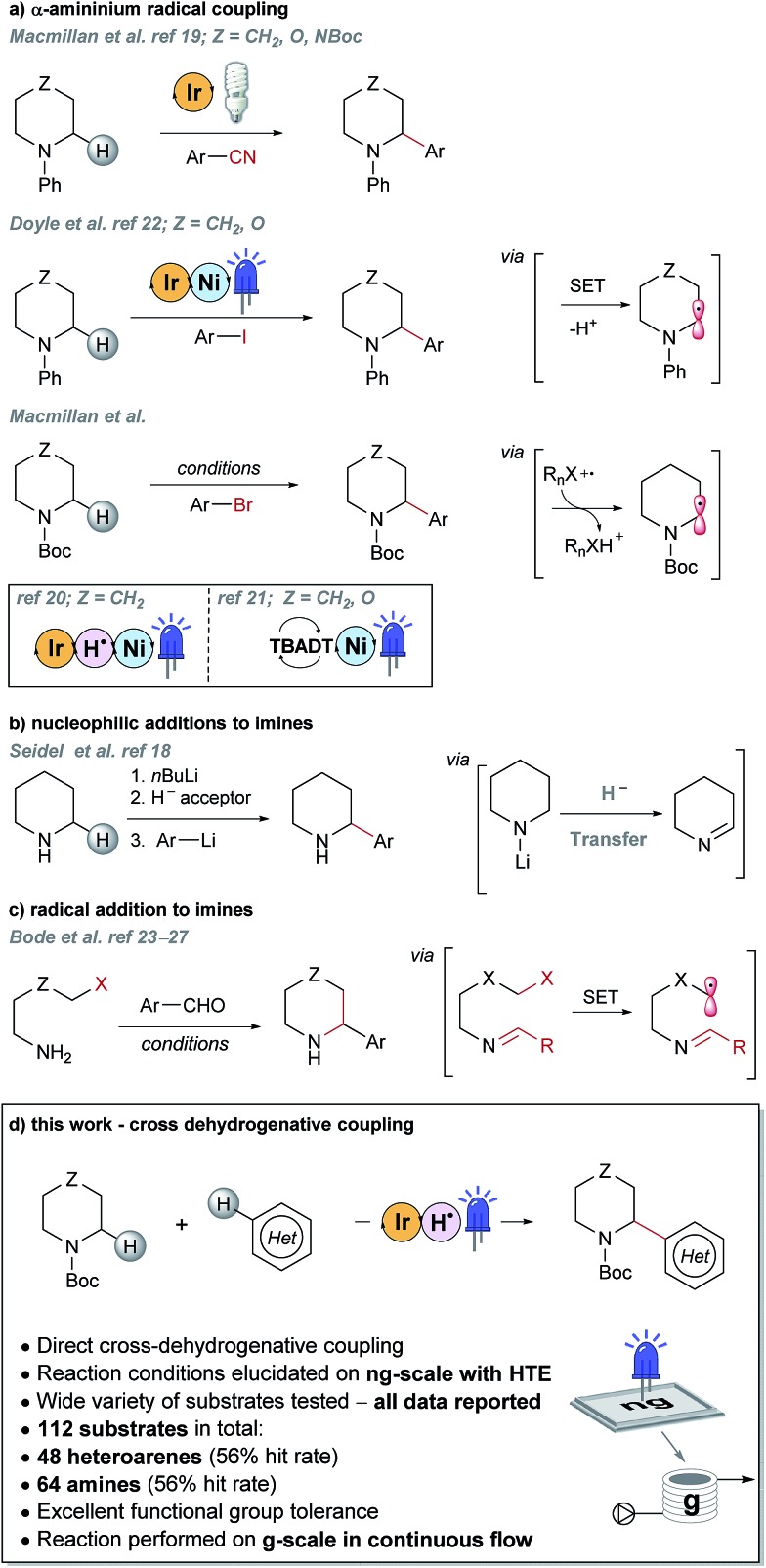
Selected examples of literature precedents for the formation of sp^2^–sp^3^ elaborated α-aryl cyclic amines and the new photoredox-mediated cross-dehydrogenative method reported herein.

Some recently reported methods allowing access to sp^2^–sp^3^ elaborated cyclic amine-containing systems are shown in Fig. 2.^15^,^19^–^27^ Noteworthy are metallophotoredox systems which permit direct C–H functionalization of cyclic amines under mild conditions (Fig. 2a).^19^–^21^ We were attracted by advances in photoredox catalysis^28^–^33^ and saw the opportunity to apply this technology to direct fragment elaboration. Unlike transition-metal catalyzed cross-couplings which are often impeded by the presence of Lewis basic nitrogen atoms, outer-sphere single electron transfer (SET) processes tolerate polar substrates and exhibit excellent functional group compatibility. Furthermore, photoredox reactions are often performed in polar solvents, a consideration important when developing chemistry on water-soluble polar molecules.^34^ Inspired by advances in α-amino radical coupling chemistry^19^–^21^,^33^ (Fig. 2a) and recent reports of homolytic C–H scission alpha to alcohols and ethers,^35^–^37^ we started by investigating the C–H functionalization of morpholines, where generation of a single electron species on C2 or C3 seemed feasible. With our interest in the elaboration of heteroarenes, a Minisci-type approach where the C-centered radical attacks an electron deficient arene was attractive.^35^,^38^–^42^ The reported metallophotoredox methods highlighted in Fig. 2a require pre-functionalization of the heteroaryl coupling partner, and a cross-dehydrogenative approach would allow us to simultaneously explore direct C–H functionalization of the cyclic amine (*c.f.* morpholine) and heteroaromatic fragments (*e.g.* isoquinoline) in tandem, without the need for pre-installed reactivity handles (Fig. 2d). To speed up the discovery of conditions to achieve this coupling, we used our in-house developed nanogram-to-gram workflow, involving nanoscale high-throughput experimentation (HTE) in 1536 well microtiter plates (MTPs), dosed by liquid handling robots. This nanoscale HTE front-end was inspired by the pioneering work from Merck but utilizes continuous flow technology to upscale hits identified on the nanogram-scale screen to generate useful quantities of the material.^43^,^44^

## Results and discussion

### Reaction conditions established with nanoscale high-throughput experimentation (HTE)

**Fig. 3:**
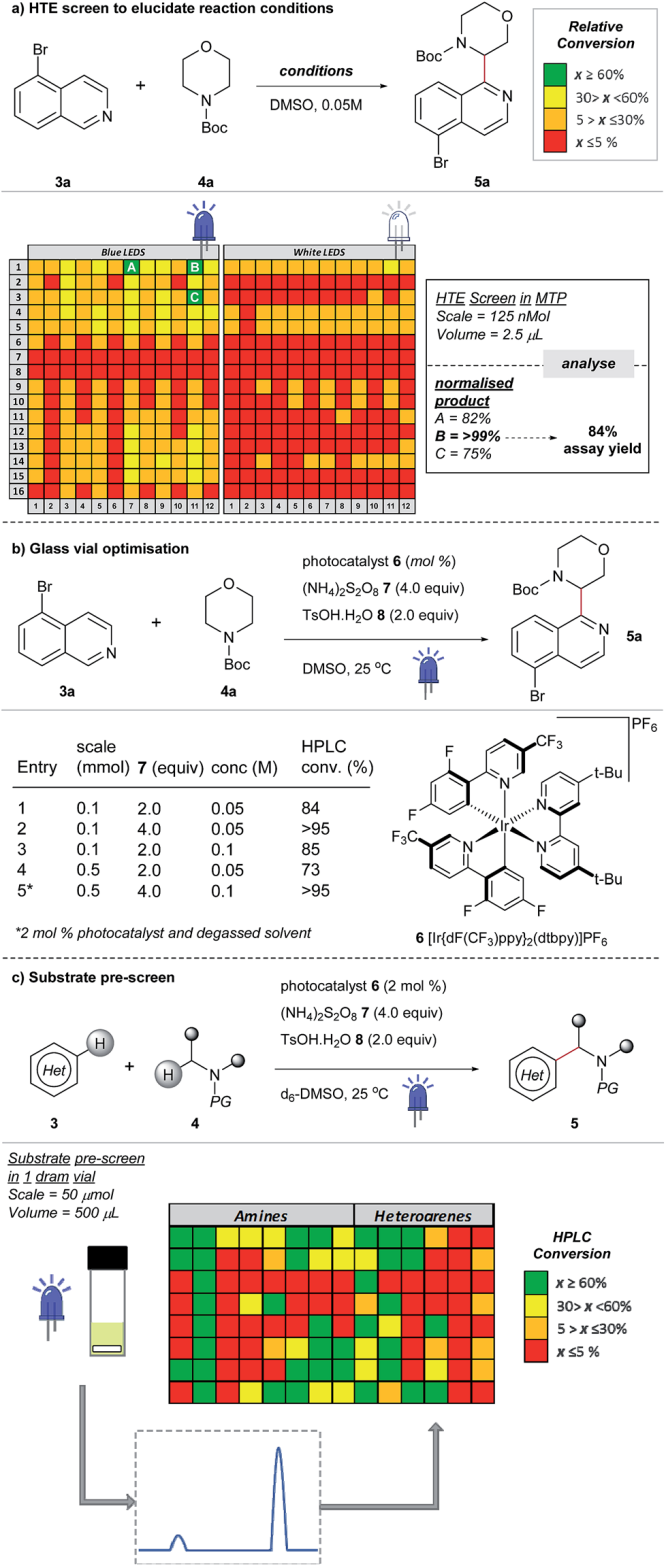
Development of a new cross-dehydrogenative coupling of heteroarenes and heterocycles using an initial nanomolar HTE screen in a 1536-well MTP.

Most examples of α-functionalization of morpholines and piperazines require *N*-arylated substrates to facilitate α-amino radical generation *via* initial oxidation to the iminium radical cation followed by deprotonation.^19^,^22^ We desired a reaction that was applicable to aliphatic amines; thus we chose a substrate with the readily removable *tert*-butyloxycarbonyl (Boc) group^20^,^21^,^33^ (for a recent example of direct sp^3^–sp^3^ functionalization of carbamyl protected amines reported by the Nicewicz group, see ref. 45). Moreover, we desired a reaction that would tolerate common synthetic handles and we first investigated the coupling reaction between 5-bromoisoquinoline **3a**, bearing a bromine functional handle for subsequent elaboration, and *N*-Boc morpholine **4a** (Fig. 3a). Using high-throughput (HTE) in 1536-well microtiter plates (MTPs), 768 reactions were screened on the nmol scale; two sets of 192 individual combinations were performed in duplicate, with one set irradiated with blue LEDs and the remainder with white LEDs (Fig. SI-2, SI-3 and SI-5; ESI†).^46^ The heatmap details the reaction outcome based on relative HPLC conversion (product/internal standard), from this initial screen only three combinations exhibited significant amounts of the product. Pleasingly, reaction conditions B (quantitative conversion in 1536-well MTPs on the nmol scale)^47^ translated to 84% conversion in a 5 mL crimp top glass vial on the 0.1 mmol scale, as determined by HPLC assay (Fig. 3a and b, entry 1). Like many photoredox catalyzed processes, the reaction was sensitive to the scale.^48^,^49^ Decreased conversion was observed on moving from MTPs (>99% conversion, 125 nmol, 2.5 μL) to the 5 mL glass vial (84% conversion, 0.1 mmol, 2 mL) with the effect more pronounced on scaling to a 30 mL glass crimp top vial (73% conversion, 0.5 mmol, 10 mL) (see Table SI-1, entries 1, 2 & 6; ESI).† 50 Consequently, secondary optimization in glass vials was performed before exploring the substrate scope. Quantitative conversion in the coupling of **3a** and **4a** was achieved by increasing the loading of photocatalyst **6** to 2 mol%, HAT catalyst 7 to 4.0 equivalents and excluding air from the reaction by nitrogen-sparging the solvent for 10 min prior to irradiation (Fig. 3b, entry 5). It should be noted that during the preparation of this manuscript, conditions that effect the cross-dehydrogenative coupling of carbamyl amines and heteroarenes were also disclosed by Berthelot *et al.* and Wang *et al.*^42^

### Reactivity mapping on the milligram scale

After optimizing conditions for the 0.5 mmol scale, a substrate pre-screen was performed on the 50 μmol scale in 0.5-dram glass vials to map out the structure–reactivity relationships (SRRs) of the methodology, with the output displayed as a heat map, based on HPLC conversion (Fig. 3c). Coupling of 64 amines was screened against substrate **3a**, and 48 heteroarenes were investigated using **4a** as the amine coupling partner. In total, 112 substrates were assessed with a hit rate of 56% (>5% conversion), and 33% of the substrates screened exhibited ≥ 60% conversion *via* HPLC analysis. To fully disclose the scope and limitations of the reaction, an exhaustive list of all substrates tested is included in the ESI (Tables SI-5 and SI-6†). Fragments and compounds with drug-like properties generally contain unprotected polar functionalities,^34^ and therefore it was important that such motifs were well represented in our study.

A selected scope of the cross-dehydrogenative coupling of **3a** and a variety of cyclic amines is shown in Table 1. The reaction tolerates a variety of protecting groups in addition to Boc (Ac **5j**, and Cbz **5f**, Table 1; CHO and Piv, Table SI-5, ESI†). For morpholines, it is noteworthy that no reaction was observed at C2 (Table 1, **5a** and **5m–w**) unlike previous reports of Minisci-type couplings of cyclic ethers,^35^ or at C2 in thiomorpholine^33^ (Table 1, **5i**). *N*-Substituents which are not tolerated include strongly deactivating groups such as tosyl- and trifluoroacetyl- (Table SI-5, ESI†). The reaction also fails in the presence of tertiary alkyl amines and *N*-aryl amines even if a suitable carbamyl-protected aliphatic amine is available for α-functionalization (Table SI-5, ESI†). We hypothesize that the presence of these moieties results in an undesirable reductive quenching of the catalyst.^32^

**Table 1:** Scope of cross dehydrogenative heteroarylation of cyclic amines^*a*^

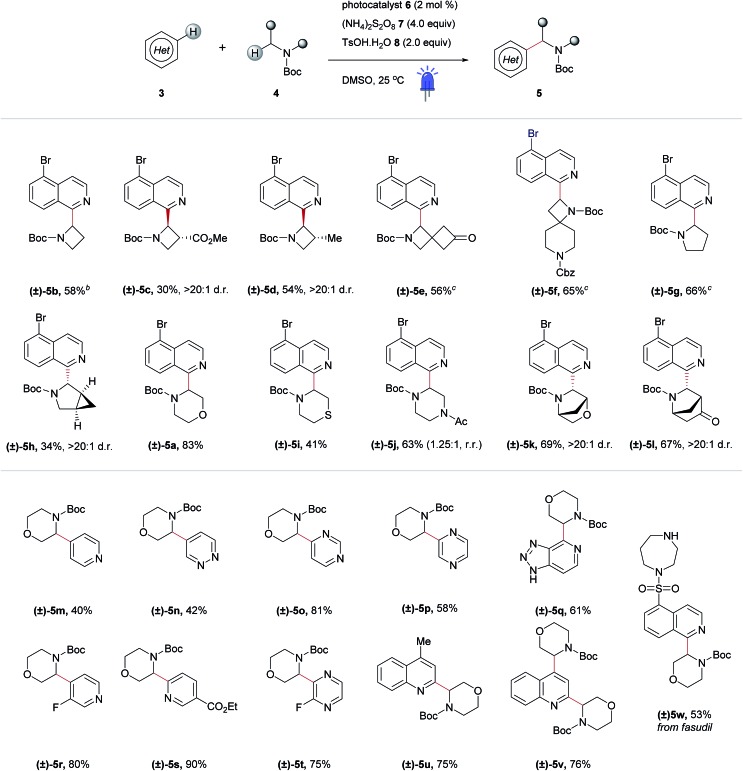

^*a*^Reactions were performed on the 0.5 mmol scale following the procedures outlined in the Experimental section and in the ESI. Reported yields are of the pure, isolated material. Photocatalyst **6**: [Ir{dF(CF_3_)ppy}_2_(dtbpy)]PF_6_.

^*b*^1.5 equivalents of the amine coupling partner.

^*c*^3.0 equivalents of the amine coupling partner. Ac = acetyl; Boc = *tert*-butyloxycarbonyl; Cbz = carboxybenzyl; Et = ethyl; Me = methyl; d.r. = diastereomeric ratio; r.r. = regioisomeric ratio.

A striking feature of this reaction is the ability to rapidly generate homologous series of 4-, 5- and 6-membered rings with a variety of endocyclic heteroatoms (N, O, and S) and privileged structures such as spirocycles (Table 1, **5e and f**) and bridged bicycles (Table 1, **5h**, **5k and l**). Moreover, SRRs are observed with a ring size: the 4- and 5-membered rings azetidine and pyrrolidine are more reactive than piperidine, as confirmed in the case of spirocyclic compound **5f** where the azetidine ring reacts preferentially. The reactivity of the amine coupling partner is not only attributable to the ring size but also stereoelectronic effects. For example, in the case of fasudil **5w**, the reaction occurs exclusively at isoquinoline C2 and morpholine C3, with no functionalization of the homo-piperazine ring observed (Table 1). Moreover, the α-heteroarylation of piperazine can be controlled with moderate selectivity using an orthogonal protecting group strategy (Table 1, **5j**). The reaction is slightly favored on the carbon adjacent to the carbamate-protected nitrogen over the acetylated nitrogen (1.25 : 1, respectively). This ratio could potentially be improved using a protecting group that tolerates the reaction conditions but does not promote α-amine functionalization (*e.g.* arylsulfonyl, *c.f.*Table 1, **5w** and Table SI-5, ESI†).

**Fig. 4:**
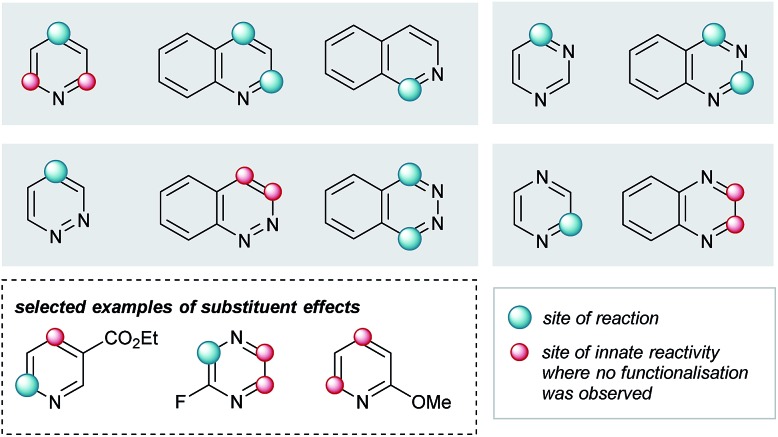
Observed substituent effects can permit reactivity modulation in diazines and naphthyridines.

Elaboration of **4a** was investigated using a range of heteroarenes (Table 1, **5a** and **5m–w**). With pyridine, the reaction occurs exclusively at C4 (Table 1, **5m** and Fig. 4), where the frontier orbital coefficient on the pyridinium cation is highest.^51^ Similarly, substitution occurs at the C4 of 3-fluoropyridine (Table 1, **5r**) in improved yields, attributable to the *ortho*-promoting effect of the fluorine.^52^ This effect is also observed with 2-fluoropyrazine (Table 1, **5t**), where alkylation was observed exclusively at C3, adjacent to the fluorine atom. In the Minisci-type addition of alkyl radicals to azines, an ester promotes reaction in the *para* position;^52^ accordingly, ethyl nicotinate reacts exclusively at C6 (Table 1, **5s** and Fig. 4). Thus, with a judicious substituent choice, other growth vectors on these ring systems can be accessed by biasing functionalization away from the sites of innate reactivity.

Coupling of **4a** was also performed with a number of 5,6- and 6,6-bicyclic nitrogenous heteroarenes. These compounds are ubiquitous within medicinal chemistry and are well-recognized fragments; a selection of successful substrates is reported in Table 1 (**5a**, **5q**, and **5u–w**) with the full range of heteroarenes tested detailed in Table SI-6 (ESI†). As shown in Fig. 4, interesting SRR effects were observed in the 6-membered monocyclic and the corresponding 6,6-fused bicyclic systems. In some cases, the fused bicyclic system is more reactive than the monocyclic heteroarene, with over-reaction observed in the case of quinoline (*c.f.* exclusively mono-alkylated regioisomer – pyridine **5m**, and bis-alkylated regioisomer quinoline **5v**; Table 1 and Fig. 4). And, in other cases the bicycle is completely unreactive under the reaction conditions (*c.f.* pyrazine and quinoxaline, Fig. 4).

The reported photoredox method exhibits good tolerance to a variety of functional enablers for further elaboration (including nitriles, esters, ketones, amides and halogens) and is compatible with unprotected polar functionalities (aliphatic and aromatic NH) and nitrogen-rich heterocycles.

### Upscaling to the gram scale using flow chemistry

**Fig. 5:**
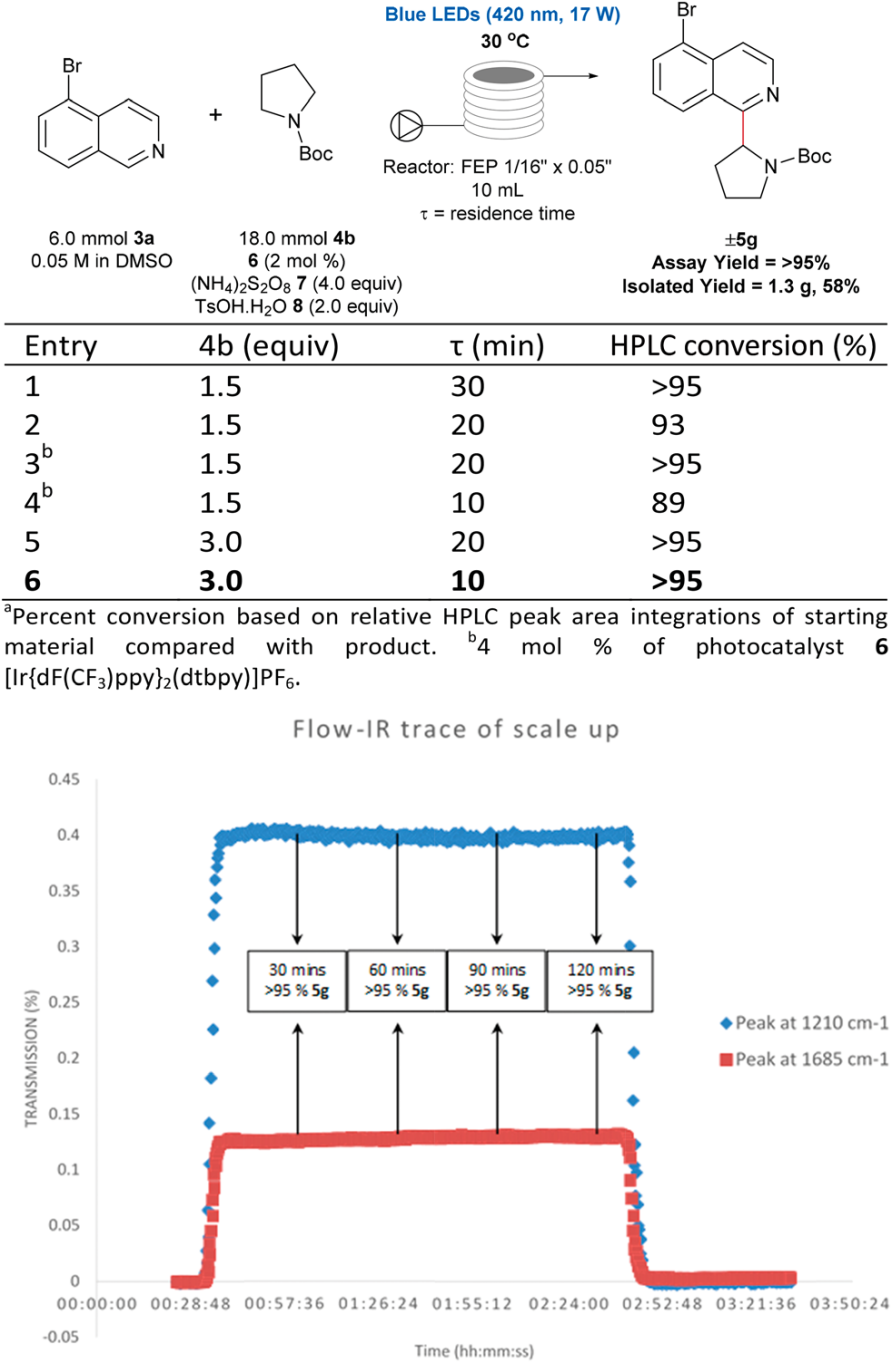
Flow scale-up of 5-Br isoquinoline **3a** and *N*-Boc pyrrolidine. Under optimized conditions, a flow rate of 1 mmol min^–1^ (*τ* = 10 min) was achieved. Using inline IR monitoring, the process was shown to be stable and can be operated at the steady state for prolonged periods, demonstrating the feasibility of the process for multi-gram production.

There are numerous successful reports of photoredox chemistry in flow,^53^–^60^ considering the previously mentioned scalability issues of our process in batch, continuous flow seemed the most effective approach to generate gram-quantities of heteroarylated cyclic amines. Pyrrolidine is one of the most abundant non-aromatic nitrogen heterocycles in pharmaceuticals,^13^,^14^ and hence, we chose to evaluate the coupling of *N*-Boc pyrrolidine **4b** and 5-bromoisoquinoline **3a** on the gram-scale. As shown in Fig. 5, coupling of *N*-Boc pyrrolidine **4b** and 5-bromoisoquinoline **3a** was investigated in flow to identify compatible conditions. Quantitative conversion is achieved at 30 °C with a 30 min residence time (*τ*) in a 10 mL photoreactor with a slight excess of amine (1.5 equivalents) and decreasing the concentration to 0.05 M; residence times can be shortened to 10 min by increasing the loading of the coupling partner (**4b**) to 3 equivalents. Thus, with a flow rate of 1 mL min^–1^ (*τ* = 10 min, 3 mmol **3a** can be processed per hour; after aqueous extraction and chromatographic purification, 1.3 g of pure material can be generated with a 2 hour run time (see ESI Table SI-4, Fig. SI-7 and SI-8† for more details). Furthermore, using in-line IR monitoring we could show that the process could be operated at the steady-state for an extended period of time, demonstrating the feasibility of this process for production of significant amounts of the material (Fig. 5).

## Conclusions

**Fig. 6:**
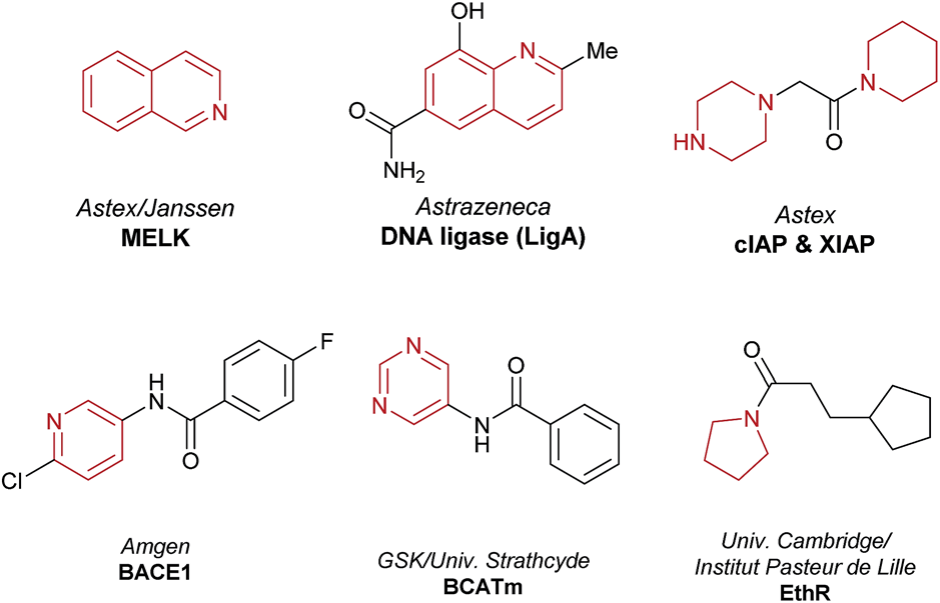
Fragment hits used as starting points for F2L campaigns reported in 2015 and 2016. All contain heterocycles within the reactivity scope of the C(sp)^2^–C(sp)^3^ cross-dehydrogenative coupling reported herein.

This study denotes the first report of a HTE-based workflow for the identification and development of a photoredox-mediated C–H functionalization reaction. The workflow involves (i) reaction discovery using HTE on the nanomolar scale in 1536-well MTPs, (ii) optimization on the milligram scale in glass vials, and finally, (iii) scale-up using continuous flow to afford gram-quantities of the material. We have shown that this nanogram-to-gram workflow is applicable to the *de novo* identification of a reaction compatible with medicinally relevant heterocyclic architectures. This photoredox-mediated C(sp)^2^–C(sp)^3^ method allows for the direct linkage of N-rich fragment-like molecules such as morpholines and isoquinolines without the need for pre-installed reactive functionalities. Moreover, a number of aromatic and aliphatic heterocycles are exemplified within the substrate scope, which are present in fragment hits, as highlighted in recent reviews (Fig. 6).^6^,^7^ This new methodology now expands the scope of the reactions possible on these architectures and offers potential for direct modification of native fragments *via* C–C bond formation along growth vectors that may be difficult to access by other means. Moreover, while investigating the scope of the methodology, the reactivity of a diverse array of heterocyclic molecules was mapped, with excellent functional group tolerance observed (see the ESI†). We believe that expanding and understanding SRRs for new methodologies that facilitate high value couplings *via* non-traditional disconnections (such as the C–H functionalization of complex molecules) are important and essential for removing synthetic bottlenecks from FBDD campaigns, in addition to promoting the uptake of new synthetic methods by medicinal chemists. Furthermore, this approach to substrate mapping under uniform conditions provides valuable information for the synthetic community about unreactive substrates, a vital consideration when populating data sets for machine learning algorithms and reaction prediction models.

## Experimental section

Herein, we describe a typical batch procedure for the 0.5 mmol scale photoredox-mediated sp^2^–sp^3^ cross-dehydrogenative heteroarylation of cyclic amines. Further details and information about nanomolar-scale batch and gram-scale continuous flow experiments can be found in the ESI.† Method: photocatalyst (0.02 equivalent, 0.01 mmol), heteroarene (0.5 mmol, 1.0 equivalent), amine (2.5 mmol, 5.0 equivalent), (NH_4_)_2_S_2_O_8_ (2.0 mmol, 4.0 equivalent) and TsOH·H_2_O (1.0 mmol, 2.0 equivalent) were weighed into a 30 mL crimp top glass vial equipped with a magnetic stir bar. The vial was sealed, degassed DMSO was added (5 mL) and the reaction vial was sonicated to ensure that all reagents were in solution. Following this, the solution was sparged with N_2_ for 10 min and then placed in a Evoluchem™ PhotoRedOx box (equipped with one Evoluchem™ 455 nm 18W LED) on a stirrer plate and irradiated for 16 hours with stirring at 500 rpm and the internal cooling fan switched on. After this time, the reaction mixture was basified with sat. NaHCO_3_ (20 mL) and iPrOAc (25 mL) was added, the phases were separated, the aqueous layer was extracted with iPrOAc (2 × 25 mL) and the combined organic layers were washed with a small amount of cold water (15 mL), dried (MgSO_4_), filtered and concentrated *in vacuo*. The resulting crude product was purified by flash column chromatography.

## Conflicts of interest

Dr Rachel Grainger, Dr Christopher N. Johnson and Dr Tom D. Heightman are employees of Astex Pharmaceuticals.

## Supplementary Material

Supplementary information
