# Supplementary material for: Enabling synthesis in fragment-based drug discovery by reactivity mapping: photoredox-mediated cross-dehydrogenative heteroarylation of cyclic amines
Source: Chem Sci. 2018 Dec 21;10(8):2264–71. doi: 10.1039/c8sc04789h (PMC6385880; doi:10.1039/c8sc04789h)
Supplement: Supplementary file 1 [file SC-010-C8SC04789H-s001.pdf]

Electronic Supplementary Material (ESI) for Chemical Science.
This journal is © The Royal Society of Chemistry 2019

Enabling Synthesis in Fragment-Based Drug Discovery by Reactivity Mapping: Photoredox-Mediated Cross-Dehydrogenative Heteroarylation of Cyclic Amines

Rachel Grainger[1]*, Tom D. Heightman[1], Steven V. Ley[2], Fabio Lima[2,3], Christopher N. Johnson[1]*

[1]*Astex Pharmaceuticals, 436 Cambridge Science Park, Milton Road, Cambridge, CB4 0QA.*

[2]*Department of Chemistry, University of Cambridge, Lensfield Road, Cambridge CB2 1EW, UK.*

[3]*Present address: Novartis Pharma AG, Novartis Campus, 4002 Basel, Switzerland.*

General Information S3
Nanomolar Scale Automated Chemistry Experiments in 1536 well Microtiter Plates (MTP) S4
Micro-/Millimolar Scale Chemistry Experiments in Batch S5
Screening/Optimization S5
Scale up for isolation S6
Continuous Flow Chemistry Experiments S6
Optimization S6
Gram scale reaction S6
Experimental Section S7
Initial Nanomolar Scale Screen — plate setup S7
Reaction Plate Setup S8
Analysis Plate Setup S10
Initial Nanomolar Scale Screen — plate analysis S11
Micromolar Scale Optimization S13
Photochemical reactions in flow S17
Proposed reaction mechanism S20
Reactivity Map – amine scope for coupling of 3a S21

Reactivity Map – heteroarene scope for coupling of 4a S22

Micromolar scale reactions in batch: General procedure S23

Characterization Data S24

UHPLC-MS spectra of purified compounds - method detailed on pages S3 & S4 .... S52

Exemplar spectra – Variable temperature experiments S63

Exemplar spectra – Full spectral elucidation S65

Liquid Handler Protocols S83

Andrew Alliance® S83

Source plate dosing S83

Mosquito TTP Labtech S88

Reaction plate dosing S88

Analysis plate dosing S110

References S123

General Information

Nanomolar scale reactions in 1536 well plates were performed without exclusion of air or moisture. Micro- and millimolar scale reactions carried out in glass vials were performed with exclusion of air. This was achieved by using solvent that had been sparged with N_2 for 15 mins and by purging the headspace of the reaction vial with a positive pressure of N_2 and an outlet needle. Commercial solvents and reagents were used without further purification. $\{Ir[dFCF_3(ppy)_2]dtbbpy\}PF_6$ was purchased from Strem and $(NH_4)_2S_2O_8$ was purchased from Sigma Aldrich, and both used without further purification. Reactions were performed in analytical reagent grade DMSO or d_6-DMSO (99.8%), as stated and used without purification.

Analytical TLC was performed on Macherey-Nagel Alugram® Sil G/UV_{254} TLC plates visualized using UV (254 nm) then basic $KMnO_4$ solution. Flash column chromatography was performed on a Biotage SP1 system; normal-phase chromatography performed with silica SNAP columns (32–63 µm particle size, KP-Sil, 60 Å pore size) and the stated solvent system (n.b. Petrol refers to Petroleum Ether bp 40–60°C) and reverse-phase chromatography performed with Biotage C18 Ultra cartridges with an MeCN in H_2O gradient, containing 0.1% HCO_2H.

Photochemical reactions performed in 1536 microtiter plates (MTP) were irradiated with a GLW 50W IP65 RGB LED floodlight set to blue at a distance of 5 cm from the top of the plate.

Photochemical batch reactions were performed in EvoluChem™ PhotoRedOx boxes (mono: HCK1006-01-016, duo: HCK1006-01-023) equipped with either one or two Evoluchem™ 455 nm 18W LED lamps (HCK1012-01-002).

Photochemical flow reactions were performed using a Vapourtec E-series platform equipped with the UV-150 module. This module consists of a temperature-controlled irradiation chamber where a transparent fluorinated ethylene polymer (FEP) reactor (1 mm i.d., 10 mL, S4 PN: 50-1287) is coiled around a blue LED assembly (emitting at 420 nm with a total output power of 17 W, PN: 50-4036). A 75 psi back pressure regulator was used (Kinesis, P-786) and reactions were monitored with in-line infrared (IR) monitoring using a Mettler Toledo ReactIR FD.

Micro/millimole scale reactions were monitored by LC-MS using an Agilent 1290 Infinity II series UHPLC coupled to an Agilent 6130 single quadrupole mass detector, eluting a gradient

of 3-95% (MeCN in H_2O, 0.1% HCO_2H modifier) over 0.93 minutes run on a YMC-Triart C18 50x2.0mm 1.9µm column controlled at 40°C, or a Shimadzu Nexera UHPLC coupled to a Shimadzu LCMS-2020 single quadrupole mass detector. Nano molar scale reactions were analyzed with an Agilent 1200 series LC-MS equipped with an Agilent 6140 single quadrupole mass detector, eluting a gradient of 3-97% (MeCN in H_2O, 0.1% HCO_2H modifier) over 0. .83 minutes run on a YMC-Triart C18 30x2.0mm 1.9µm column controlled at 45°C. High resolution mass spectrometry was performed on an Agilent 6550 QTOF mass spectrometer.

NMR spectra were recorded on a Bruker AV400 (Avance 400 MHz) spectrometer. Chemical shifts for ^{1}H and ^{13}C NMR spectra are reported as δ in units of parts per million (ppm) and quoted to the nearest 0.01 ppm relative to the residual protons in $CDCl_3$ (7.26 ppm, 77.16 ppm), d_6-DMSO (2.50 ppm, 39.52 ppm) or CD_3OD (3.31 ppm, 49.00 ppm). Coupling constants (*J*) are quoted in Hertz (Hz) and are reported to the nearest 0.1 Hz, with multiplicity reported according to the following convention: s = singlet, d = doublet, t = triplet, q = quartet, m = multiplet, br = broad and associated combinations e.g. dd = double of doublets. DEPT 135 and 2-dimensional experiments (COSY, HMBC, HSQC and ROESY) were used to support assignments and are reported where appropriate.

Nanomolar Scale Automated Chemistry Experiments in 1536 well Microtiter Plates (MTP)

Nanomolar scale screening reactions (125 nmol) were performed in Corning® 1536-well MTP (Corning 1536 COC White, Cat. No. 4570, Cyclic Olefin-Copolymer COC, 12.5 µL-wells, flat bottom, white). Greiner® 384-well MTPs (Cat No. 651201, Polypropylene, 120 µL, V-bottom, translucent) were used as the reagent source plate and the analysis plates. The source plate (384-well MTP) was dosed using an Andrew 1000G liquid handler (Andrew Alliance, Switzerland) equipped with Gilson Pipetman Pipettes (see: pages S8 & S83). Subsequently, this 384-well source MTP were used to dose the 1536-well reaction MTP using a Mosquito® HTS liquid handling robot (TTP Labtech, UK), using the appropriate Mosquito protocol (see: pages S8 & S88). On completion of dosing, the 1536-well reaction MTP were heat sealed with a 4titude Clear Heat Seal (4ti-0541) and irradiated with a GLW 50W IP65 LED floodlight set to blue light or white light at full power. At the end of the reaction time, the 1536-well reaction MTP were mirrored (2.0 µL) into pre-dosed 384-well analysis MTP (see: page S110). The 384-well analysis MTP were pre-dosed by hand using multichannel pipettes (E1 ClipTip Equalizer

384 12-channel); the wells dosed with a 0.510 mM solution of internal standard (**IS**) 4-bromobiphenyl in DMSO (0.050 µmol, 50 mol %, 98 µL per well). Dosing of the crude reaction mixture (2 µL) into the analysis plates was achieved using a mix aspirate, mix dispense cycle on the Mosquito® HTS liquid handling robot (see: Analysis plate dosing S110) and heat sealed with adhesive free aluminium foil seals, agitated on a plate shaker for 5 mins then subjected to LCMS analysis.

Micro-/Millimolar Scale Chemistry Experiments in Batch

Scale	50 µmol	0.1 mmol	0.5 mmol
Vial	0.5 dram flat bottomed vial (Scientific Glass Laboratories, T101/V1)	2-5 mL vial (Biotage, 354833)	10-20 mL vial (Biotage, 354833)
Stir Bar	VWR, 442-0364	VWR, 442-0401	VWR 442-0403
EvoluChem Vial Holder	2 mL vial holder (HCK1006-01-018)	8 mL vial holder (HCK1006-01-020)	20 mL vial holder (HCK1006-01-021)

Reactions were performed in glass vials equipped with stir bars (as detailed in the table above). Solid reagents were weighed by hand, liquid handling of stock solutions was performed by hand with Gilson Pipetman pipettes. Stock solutions were degassed for 10 minutes prior to dispensing into reagent vials equipped with septa and purged with nitrogen. Reaction vials were stirred and irradiated in an EvoluChem^{TM} PhotoRedOx box (mono: HCK1006-01-016, duo: HCK1006-01-023) equipped with either one or two Evoluchem^{TM} 455 nm 18W LEDs (HCK1012-01-002) as stated accordingly in the relevant optimization table, the appropriate vial holder (as detailed in the table above) and cooling by internal fan component.

Screening/Optimization

For 50 µmol and 0.1 mmol scale reactions, after the stated reaction time, a solution containing two internal standards [1,3,5-trimethoxybenzene (0.33 equiv, 66.7 mM) and 1,2,4,5-tetramethylbenzene (0.25 equiv, 50 mM) in d_6-DMSO:$CDCl_3$ (50:50)] was added to each reaction vial and the crude reaction mixture analyzed directly by ^{1}H NMR and LC-MS analysis. Assay yields are reported based on LC-MS conversion with product confirmation by ^{1}H NMR. Unfortunately yields could not be accurately determined by ^{1}H NMR as most products exhibit line broadening as a result of interconverting mixtures of rotamers.

Scale up for isolation

For 0.5 mmol scale up reactions, the crude reaction mixture was checked after 16 hours *via* LC-MS analysis, following this the product was isolated *via* aqueous extraction then subsequent purification according to the procedure detailed in the experimental section.

Continuous Flow Chemistry Experiments

Optimization

Solid reagents were weighed into a 10-20 mL vial (Biotage, 354833), degassed DMSO added and the reaction mixture sonicated until all solid had dissolved. The stock solution was degassed by N_2 sparging (10-15 mins) then volatile liquids (N-Boc pyrrolidine) added. The reaction mixture was pumped at the stated flow rate as a slug of reaction mixture pushed with DMSO through the reactor coil (10 mL reactor coil) irradiated by 420 nm LEDS (17 W total output power). The entirety of the crude reaction mixture was collected, and an aliquot analyzed directly by LC-MS to determine conversion based on coupled product and unreacted heteroarene.

Gram scale reaction

Solid reagents were weighed into a 250 mL pear shaped flask, degassed DMSO added and the reaction mixture sonicated until all solid had dissolved. The stock solution was degassed by N_2 sparging (10-15 mins) then volatile liquids (N-Boc pyrrolidine) added. The reaction mixture was pumped at 1.00 mL.min^{-1} as a slug of reaction mixture pushed with DMSO through the reactor coil (10 mL reactor coil) irradiated by 420 nm LEDS (17 W total output power). The reaction was monitored by inline IR monitoring and aliquots collected every 30 minutes and analyzed by LC-MS to monitor conversion once the system had reached steady-state. The crude reaction mixture was collected, and the product was isolated *via* aqueous extraction then subsequent purification according to the procedure detailed in the experimental section.

Experimental Section

Initial Nanomolar Scale Screen — plate setup

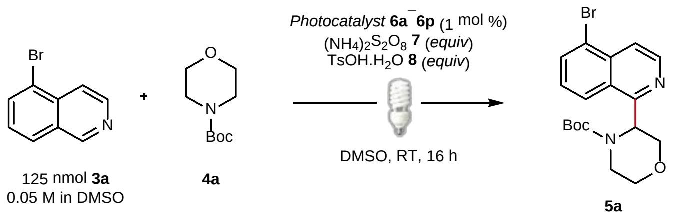

4a = *1.25, 3.0, 5.0 equiv*
Photocatalysts **6a**–**6p**
$(NH_4)_2S_2O_8$ **7** = *1.0, 2.0 equiv*
TsOH **8** = *0, 2.0 equiv*

Light source:
50W input *Blue* LED floodlight
50W input *White* LED floodlight

768 duplicate reactions
384 individual combinations

Photocatalysts

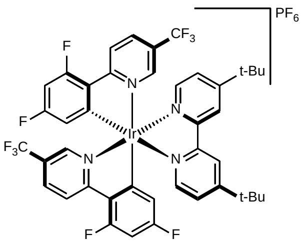

6a
[Ir(dF(CF3)ppy)2dtbpy]PF6

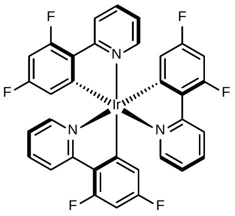

6b
fac-Ir(Fppy)

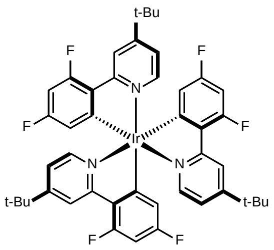

6c
fac-Ir(dF(tBu)ppy)

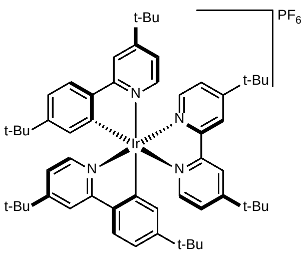

6d
[Ir(dtbppy)2dtbpy]PF6

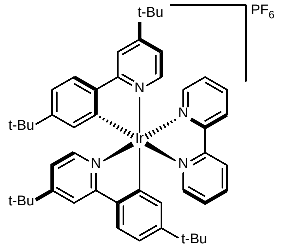

6e
[Ir(dtbppy)2bpy]PF6

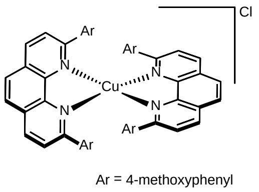

6f
[Cu(dap)2]Cl

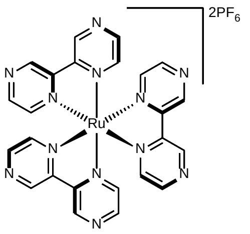

6g
[Ru(bpz)3][PF6]2

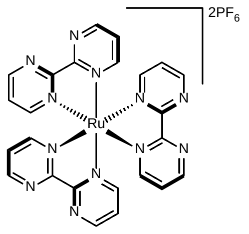

6h
[Ru(bpm)3][PF6]2

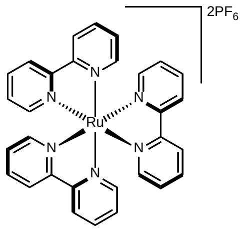

6i
[Ru(bpy)3][PF6]2

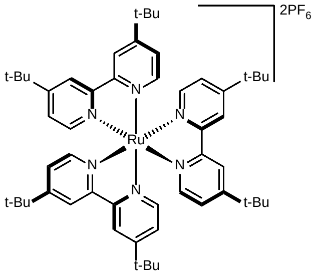

6j
[Ru(dtbpy)3][PF6]2

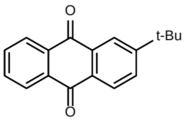

6k
tert-butyl anthraquinone

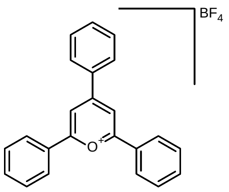

6l
Triphenylpyrylium

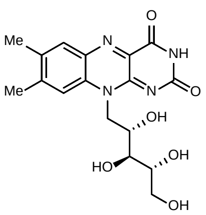

6m
Riboflavin

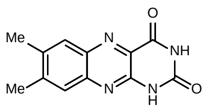

6n
Lumichrome

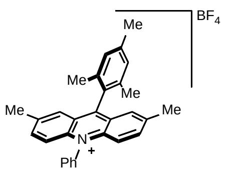

6o
[Mes-diMeAcr-Ph]BF4

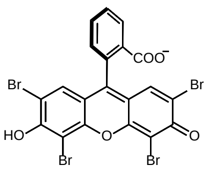

6p
Eosin Y

Reaction Plate Setup

A 384 MTP source plate was dosed in a matrix using Andrew Alliance® 1000G liquid handler (Figure SI-1), this source plate was then used to dose one quarter of two separate 1536 MTPs with TTP Labtech Mosquito® HTS to generate two identical reaction plates (layouts depicted in (Figure SI-2). In each reaction plate, 192 individual combinations were performed in duplicate; the individual combinations comprise a cross screen of 3 stoichiometries of amine coupling partner **3a**, 16 commercially available photocatalysts **6a–6p**, and a range of loadings of the hydrogen atom transfer (HAT) catalyst **7** and *p*-toluenesulfonic acid **8**. Each plate was heat sealed with 4titude Clear Heat Seal (4ti-0541) and irradiated with a GLW 50W IP65 LED floodlight set to cool white or blue light at full power.

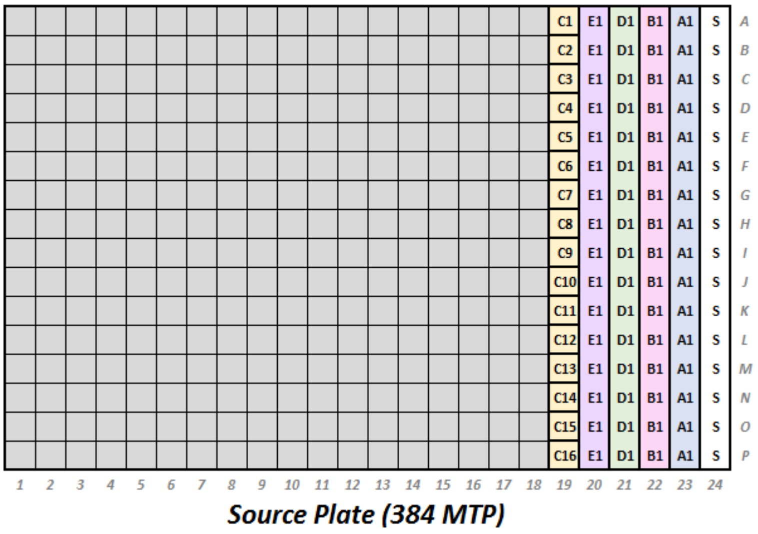

Andrew Alliance 1000G Dosed Protocol: *Source plate dosing*

S = DMSO
A1 = **3a**; 0.25 M
B1 = **4a**; 1.25 M
C1–**C16** = **6a–6p**; 2.5 x 10^{-3} M
D1 = **7**; 0.5 M
E1 = **8**; 0.5 M
All stock solutions in DMSO

Figure SI-1: Source plate layout, reagents dosed using Andrew Alliance® 1000G liquid handler

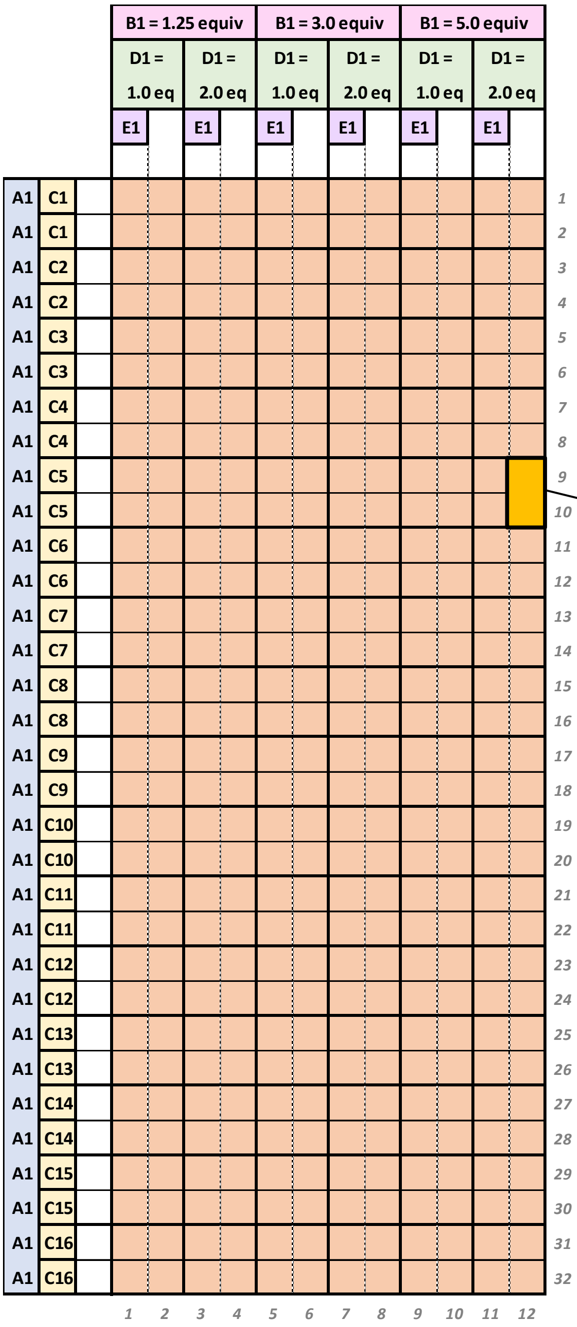

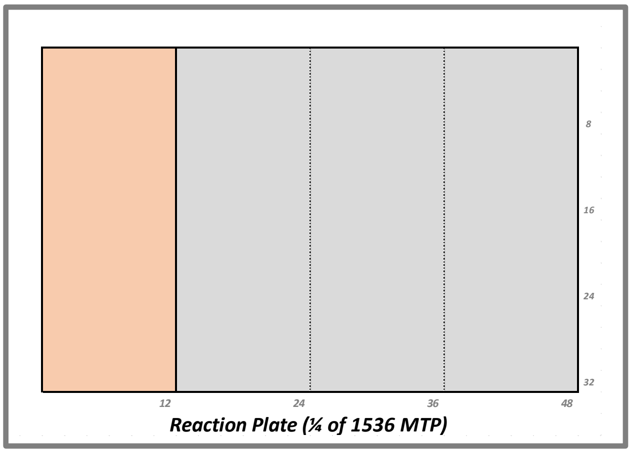

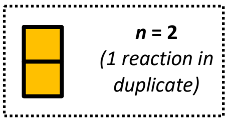

TTPLabtech Mosquito®
***Protocol**: Reaction plate dosing*

<u>Dose Order and Amounts</u>
1st: **A1** = **3a**; 500 nL
2st: **B1** = **4a**; 125, 300, 500 nL
3rd: **S** = DMSO, 0–1125 nL
4th: **D1** = **7**; 250, 500 nL
5th: **E1** = **8**; 500 nL
6th: **C1-C16** = **6a–6p**; 500 nL
(mix dispense)

Final well volume = 2.5 µL
Final well conc. = 0.05 M

Figure SI-2: Reaction plate layout. Reactions dosed using Mosquito HTS TTPLabtech® liquid handler

Analysis Plate Setup

After 16 hours, the 1536-well reaction plates were mirrored into two 384 well MTPs (Figure SI-3), each well primed with 98 µL of internal standard **IS** (4-bromo biphenyl, (50 mol %) in DMSO, 0.510 mM). The internal standard solution was pipetted by hand using a multi-channel pipette and 2 µL of the crude reaction mixture added using a mix-aspirate-mix-dispense function (TTPLabtech Mosquito® HTS). Due to the tip spacing on the Mosquito, the duplicate reactions which are positioned above and below each other in one column of the reaction plate (Figure SI-2) now appear side-by-side in two separate columns in the analysis plate (Figure SI-3). Details of the contents of each well can be found in workbook included the Supporting Information section on the journal website.

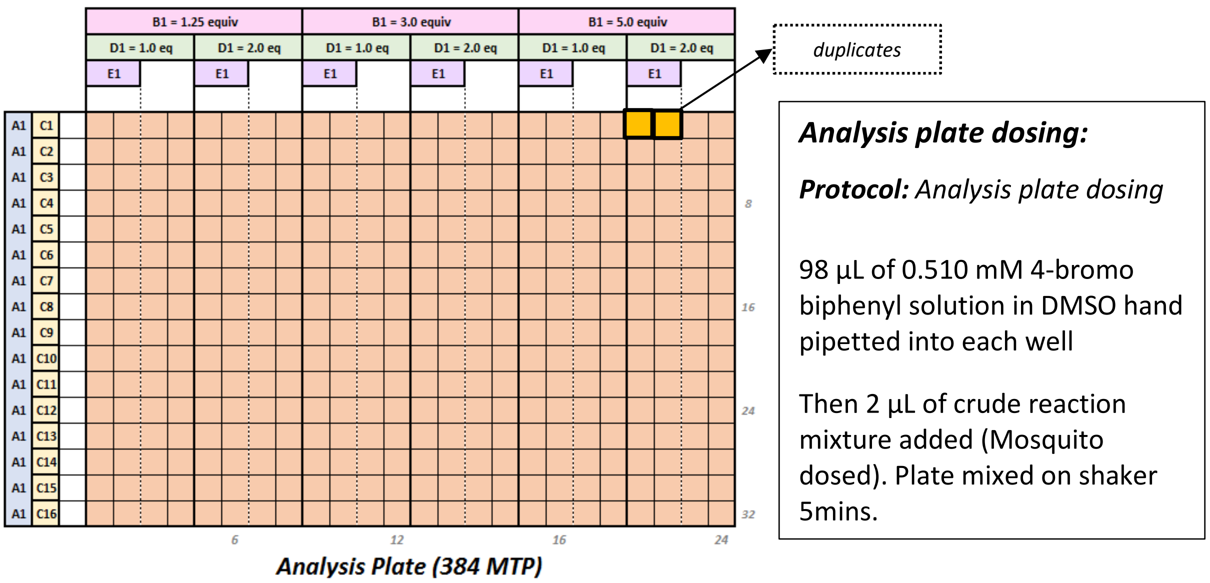

Figure SI-3: Analysis plate layout. Predosed wells charged with crude reaction mixture using Mosquito HTS TTPLabtech® liquid handler

Initial Nanomolar Scale Screen — plate analysis

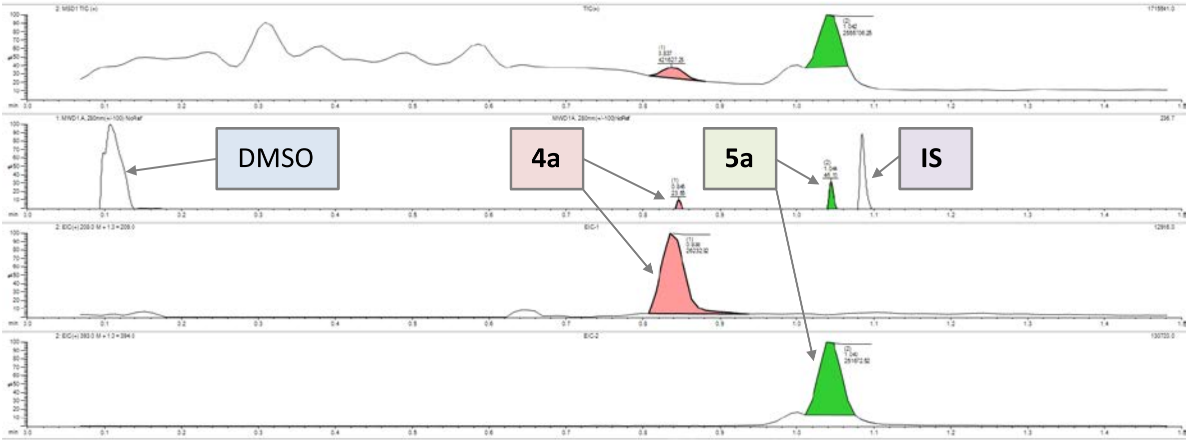

Figure SI-4: Exemplar LC-MS spectra of crude reaction showing retention times of 5-Br isoquinoline starting material **4a**, coupled product **5a** and 4-bromo biphenyl internal standard **IS.**

The crude reactions were analyzed by LC-MS (see General Information section for equipment and gradient specifics) the product **5a** and internal standard peaks on the HPLC trace were integrated, mean average taken and the resulting ratio of **5a**:**IS** calculated, the data is normalized to the largest value in the set plotted in a heat map.[1]

[1] Individual reaction well combinations and corresponding LC-MS data available in Supporting Information section on the journal website.

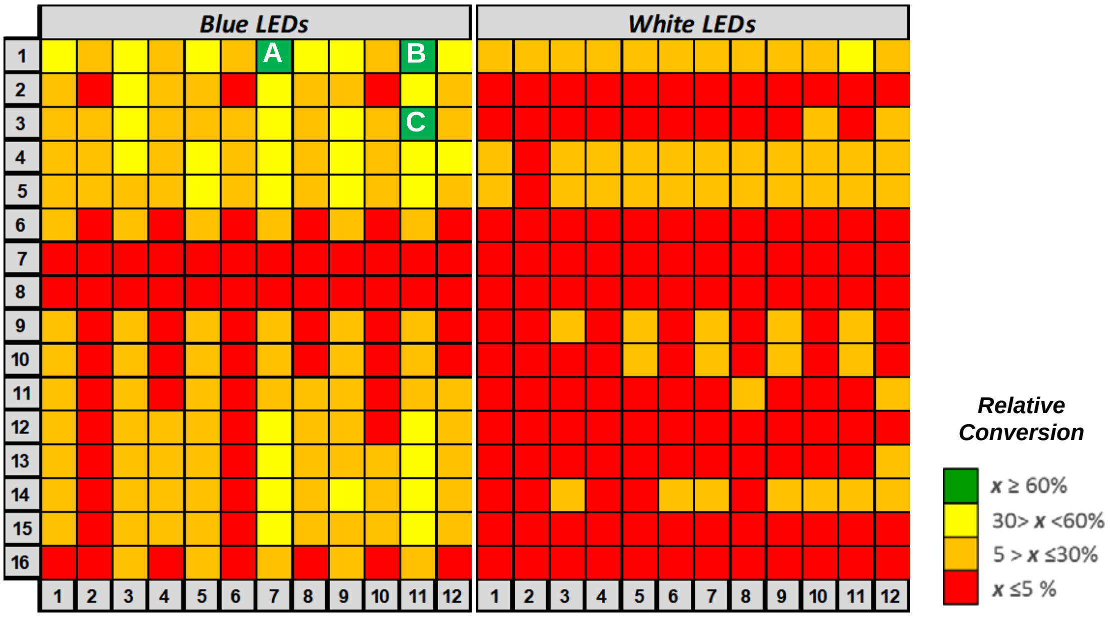

	Product/IS (mean)	Product/IS % (normalized to largest value in set)	Optimal Conditions = B
A	0.91	82	**Blue Light**<br>**3a** = 125 nmol, 0.05 M in DMSO<br>**4a** = 5.0 equiv<br>**6a** = [Ir(dF(CF$_3$)ppy)$_2$dtbpy]PF$_6$<br>**7** = 2.0 equiv<br>**8** = 2.0 equiv
B	1.11	>99	
C	0.83	75	

Figure SI-5: Heatmap of nanomolar screen and corresponding hit conditions

Micromolar Scale Optimization

Table SI-1: Coupling of **3a** and **4a** – Optimization of stoichiometry and concentration

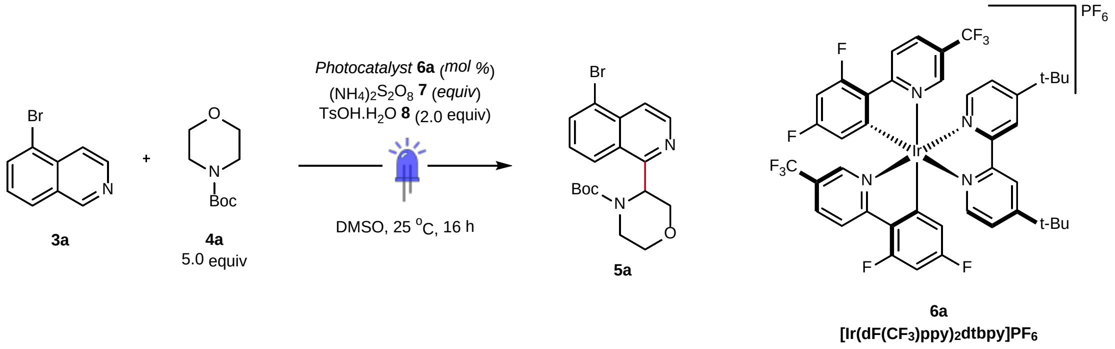

Entry	Scale (mmol)	**6a** (mol %)	**7** (equiv)	conc (M)	Conversion (%)[a]
1[b]	1.25 x10^{-4}	1	2.0	0.05	>95
2	0.1	1	2.0	0.05	84
3	0.1	1	4.0	0.05	>95
4	0.1	1	2.0	0.1	85
5	0.1	1	2.0	0.2	89
6	0.5	1	2.0	0.05	73
**7[c]**	**0.5**	**2**	**4.0**	**0.1**	**>95 (83)**
8	0.5	1	4.0	0.2	54
9	2.0	1	2.0	0.2	42
10[d]	2.0	1	2.0	0.2	40
11[c]	2.0	1	4.0	0.2	76
**12[c]**	**2.0**	**2**	**4.0**	**0.2**	**>95**

Unless otherwise stated, reactions are performed in a crimp cap glass vials with no exclusion of air and irradiated in a EvoluChem PhotoRedox mono box equipped with one 18W 450-455 nm LED lamp for 16 hours. [a] Percent conversion approximation based on relative HPLC peak area integrations of starting material compared with product. [b] Reaction performed in 1536 well MTP and irradiated with a GLW 50W IP65 blue LED floodlight for 16 hours; [c] Reaction mixture sparged with N_2 prior to illumination; [d] 36 hour reaction. Yields in parentheses indicate isolated yields.

Table SI-2: Coupling of **3a** and **4a** – Solvent screen

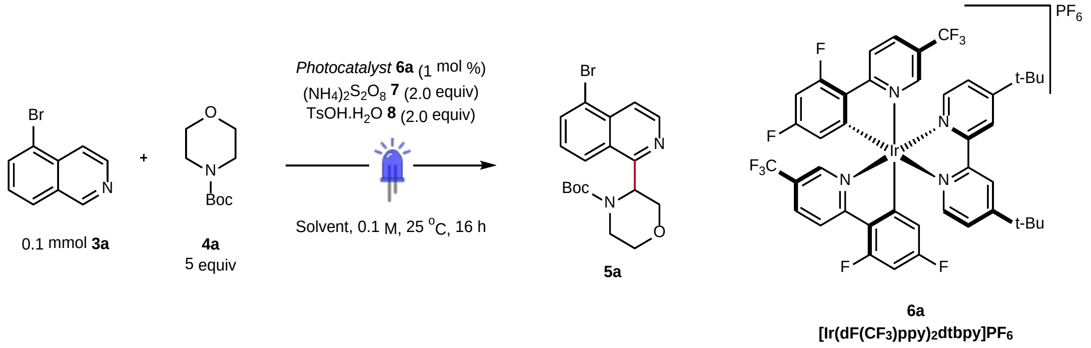

After 16 hours			*After 1 hour*		
Entry	Solvent	Conversion (%)	Entry	Solvent	Conversion (%)[a]
1	DMSO	85	11	DMSO	32
2	DMSO:H_2O (50:50 v/v)	trace	12	MeCN	N.D.
3	DMA	N.D.	13	MeCN:H_2O (90:10 v/v)	N.D.
4	DMF	N.D.	14	Acetone:H_2O (90:10 v/v)	Trace
5	NMP	N.D.	15	TFE:H_2O (90:10 v/v)	N.D.
6	tetra ethylene glycol	N.D.	16	AcOH:H_2O (90:10 v/v)	N.D.
7	TPGS-750M (2% in H_2O)	N.D.	17	PC:H_2O (90:10 v/v)	N.D.
8	MeCN:H_2O (50:50 v/v)	N.D.			
9	2-Butanone	22			
10	n-butyl acetate	N.D.			

Unless otherwise stated, reactions are performed in a crimp cap glass vials with no exclusion of air and irradiated in a EvoluChem PhotoRedox mono box equipped with one 18W 450-455 nm LED lamp for 16 hours. [a] Percent conversion approximation based on relative HPLC peak area integrations of starting material compared with product. TFE = Trifluoroethanol; PC = Propylene Carbonate

Table SI-3: Optimizing coupling **3a** and **4a** *in batch*

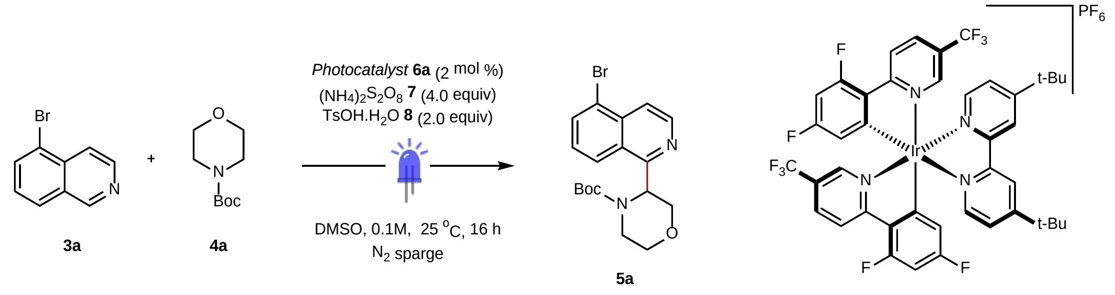

6a
[Ir(dF(CF3)ppy)2dtbbpy]PF6

Entry	**Scale** (mmol)	**4a** (equiv)	time	Conversion (%)[a]
1	0.1	5.0	16 h	>95
2	0.1	3.0	16 h	>95
3	0.1	1.5	16 h	>95
4	0.1	1.5	60 min	>95
**5**	**0.1**	**1.5**	**30 min**	**>95**
**6**	**0.5**	**5.0**	**16 h**	**>95**
7	0.5	3.0	16 h	72
8	0.5	1.5	16 h	40
9	0.5	5.0	60 min	54
10	0.5	5.0	120 min	60
11[b]	0.5	5.0	60 min	77
**12**[b]	**0.5**	**5.0**	**120 min**	**83**

Unless otherwise stated, reactions are performed in a crimp cap glass vials with no exclusion of air and irradiated in a EvoluChem PhotoRedox mono box equipped with one 18W 450-455 nm LED lamp for 16 hours. [a] Percent conversion approximation based on relative HPLC peak area integrations of starting material compared with product. [b] 2x18W 450-455nm LED lights in HepatoChem duo box.

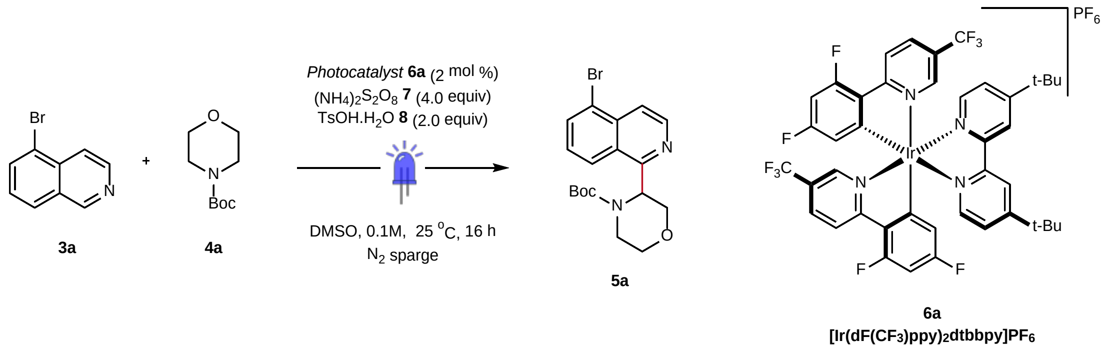

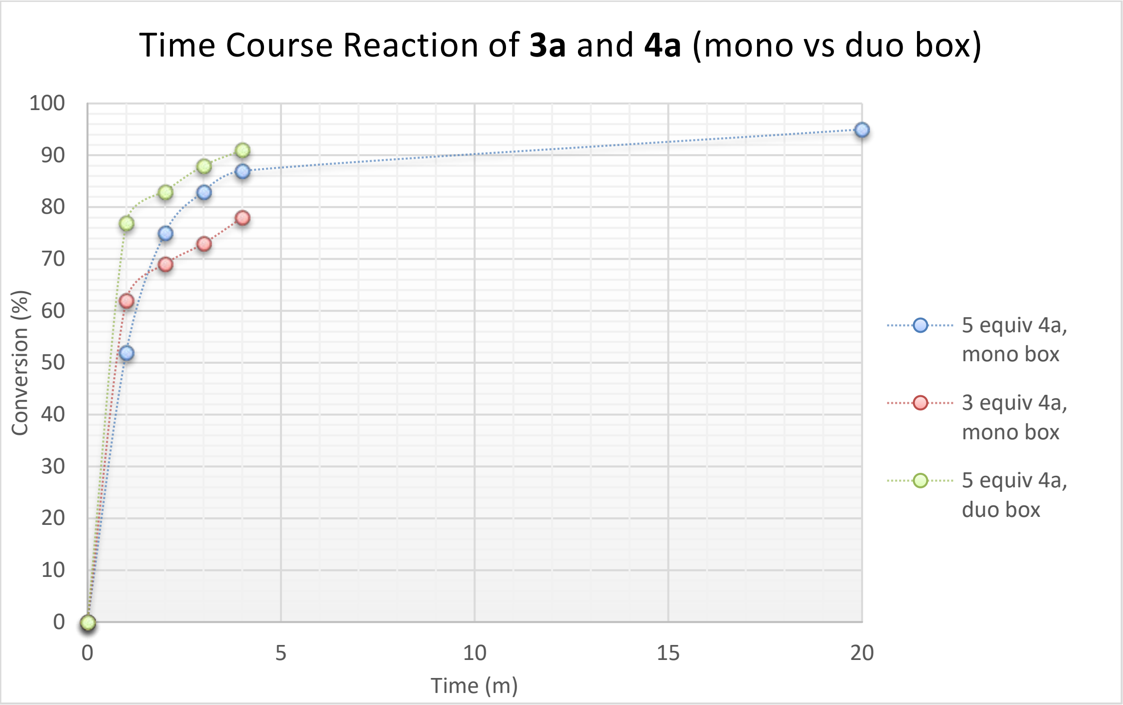

Figure SI-6: Time course of coupling of **3a** and **4a** *in batch.* Initial rate of reaction is improved on increasing the stoichiometry of the amine coupling partner and illuminating the reaction vessel with two lights.

Photochemical reactions in flow

Optimization protocol: {Ir[dFCF3(ppy)2]dtbbpy}PF_6 **6a** (0.02 equiv, 0.01 mmol), 5-bromoisoquinoline **3a** (0.5 mmol, 1.0 equiv), $(NH_4)_2S_2O_8$ **7** (2.0 mmol, 4.0 equiv) and TsOH.H_2O **8** (1.0 mmol, 2.0 equiv) were weighed into a 5 mL crimp top glass vial. The vial was sealed, degassed DMSO added (5 mL) and the reaction vial was sonicated to ensure all reagents were in solution. Following this, the solution was sparged with N_2 for 10 mins then *N*-Boc pyrrolidine **4b** added (1.5 equiv or 3.0 equiv). The clear, yellow solution was then pumped at the stated flow rate as a slug of reaction mixture pushed with DMSO through the reactor coil (10 mL reactor coil) irradiated by 420 nm LEDs (17 W total output power). Once the system had reached steady-state (as determined by in-line IR monitoring)[2] an aliquot was taken and analyzed by LC-MS to determine conversion.

Table SI-4: Optimizing coupling **3a** and **4b** *in continuous flow*

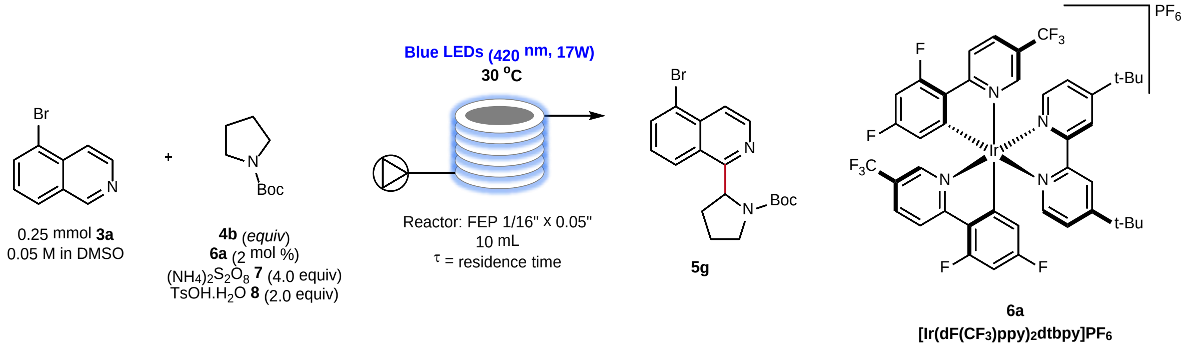

Entry	**4b** (equiv)	τ (min)	Flow rate (ml min^{-1})	Conversion (%)[a]
1	1.5	30	0.33	>95
2	1.5	20	0.50	93
3[b]	1.5	20	0.50	>95
4[b]	1.5	10	1.00	89
5	3.0	20	0.50	>95
**6**	**3.0**	**10**	**1.00**	**>95**

[a] Percent conversion approximation based on relative HPLC peak area integrations of starting material compared with product. [b] 4 mol % of photocatalyst **6a**.

[2]These peaks were chosen as they were strong signals observed in the IR spectra of the crude reaction mixture. These peaks have not been unequivocally assigned, but we hypothesize that they correspond to the ν(C=O) stretch of the Boc group on the starting material or product (1685 cm^{-1}) and a ν(S-O-H) bend of the bisulfate by-product from decomposition of **8** (1210 cm^{-1}).

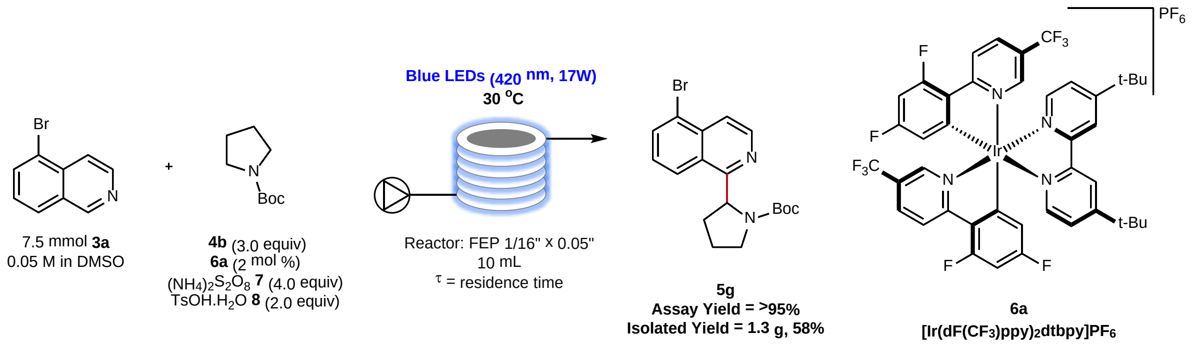

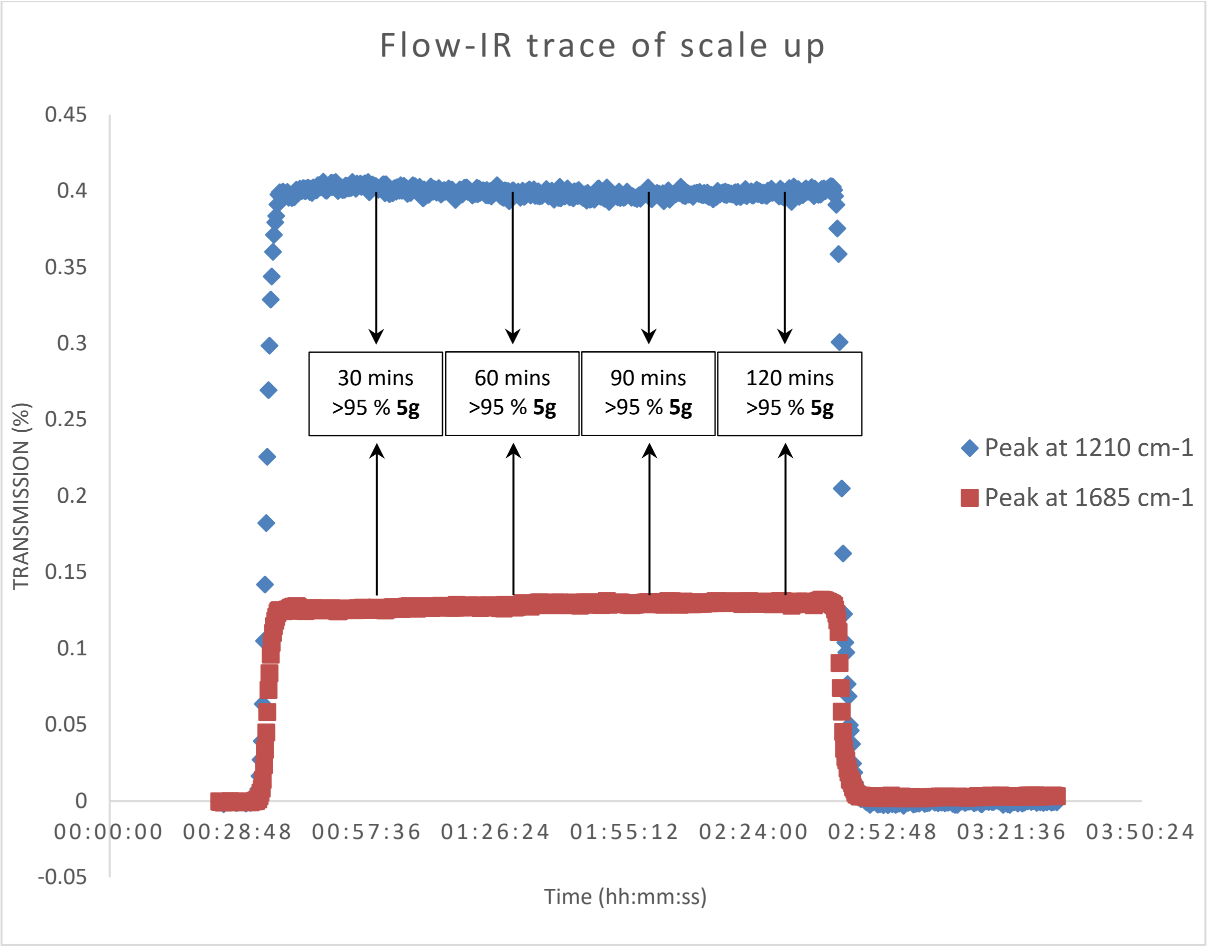

Figure SI-7: Scale up coupling **3a** and **4b** *in continuous flow*. The reaction was followed using in-line IR monitoring of signals at 1685 cm^{-1} and 1210 cm^{-1}. The IR trace shows that the steady-state process is stable over an extended period of time with no fluctuations in pressure or flow rate.

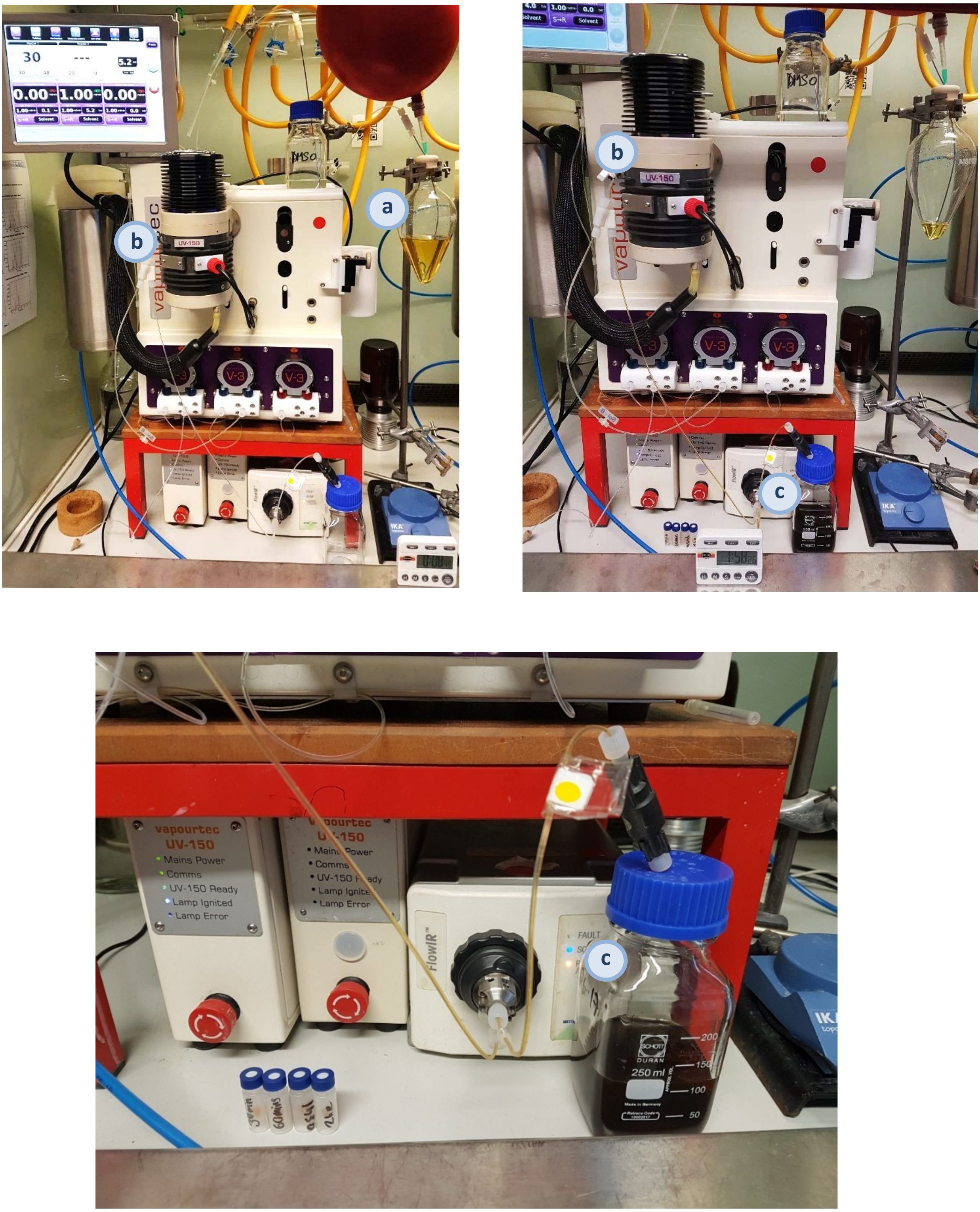

Figure SI-8: Vapourtec E-series UV-150 setup; a) Stock solution of reactants in DMSO, b) UV-150 reactor containing 10mL coil and 420 nm 17W LED lamp; c) crude reaction mixture following irradiation.

Proposed reaction mechanism

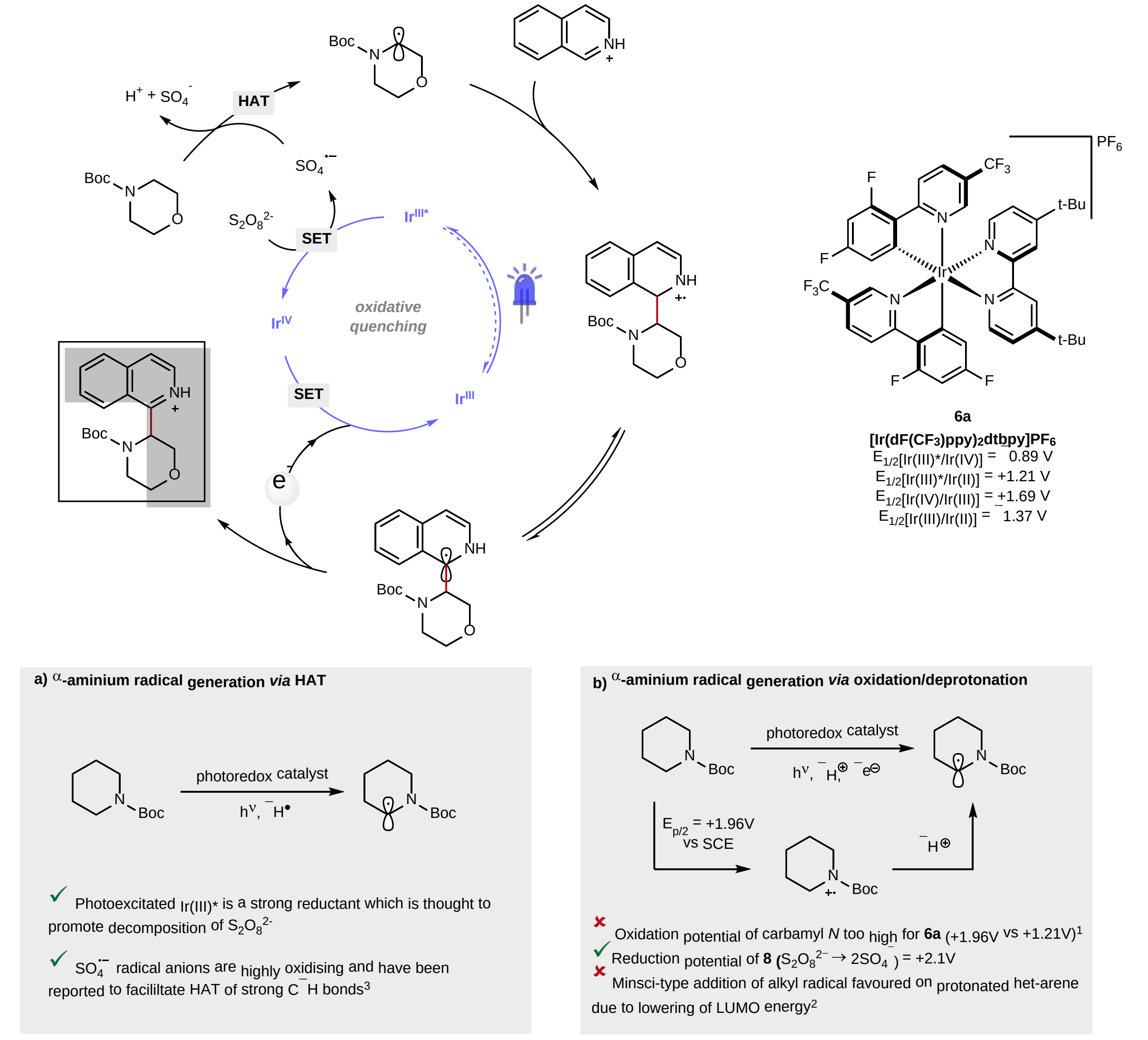

Figure SI-9: Proposed reaction mechanism.[i,ii,iii]

We hypothesize that the reaction proceeds *via* Minisci-type addition of an α-amino radical into a protonated heteroarene. Generation of the α-amino radical could occur through hydrogen atom transfer (HAT) from a reactive oxygen species generated from decomposition of the persulfate **7** (Figure SI-9a). However, and alternative mechanism could exist where the α-amino radical is generated *via* oxidation of the amine lone pair by the Ir catalyst **6a** or persulfate **7** followed by deprotonation (Figure SI-9b).

Reactivity Map – amine scope for coupling of 3a

Table SI-5: Heat map for coupling of **3a** with a variety of cyclic amines[3,4,5]

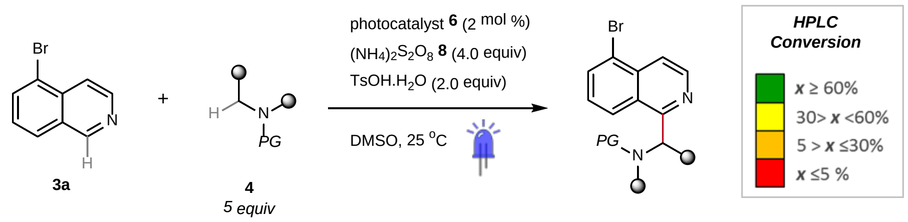

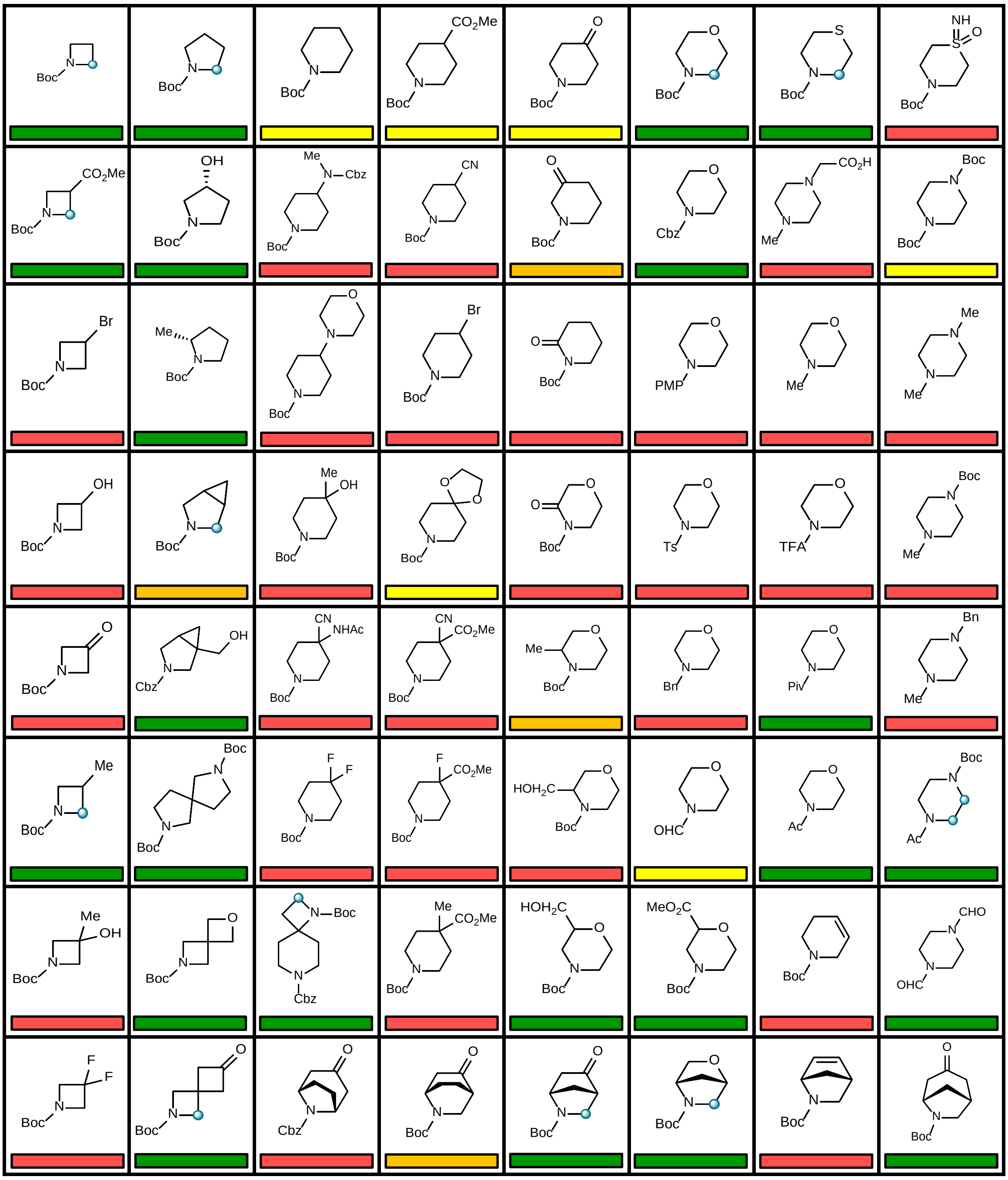

[3] Assay yield determined through HPLC conversion of starting material to product.
[4] Site of reaction observed on fully characterized product denoted by (●).
[5] In the cases of unsymmetrical, substituted 5- and 6-membered amines (*), there is evidence of more than one product with the same desired mass, as observed by LC-MS. However, as these compounds were not isolated and fully characterized, we cannot confidently determine whether this additional product is a diastereomer or regioisomer, although the former is likely.

Reactivity Map – heteroarene scope for coupling of 4a

Table SI-6: Heat map for coupling of **4a** with a variety of heteroarenes[6,7]

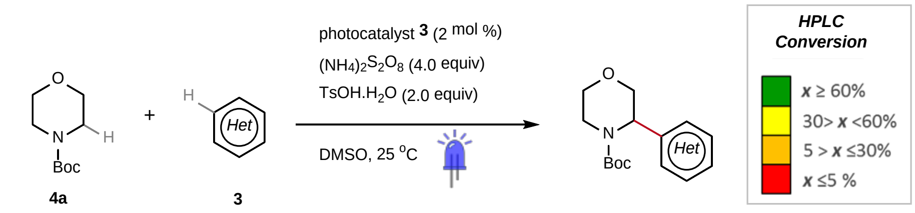

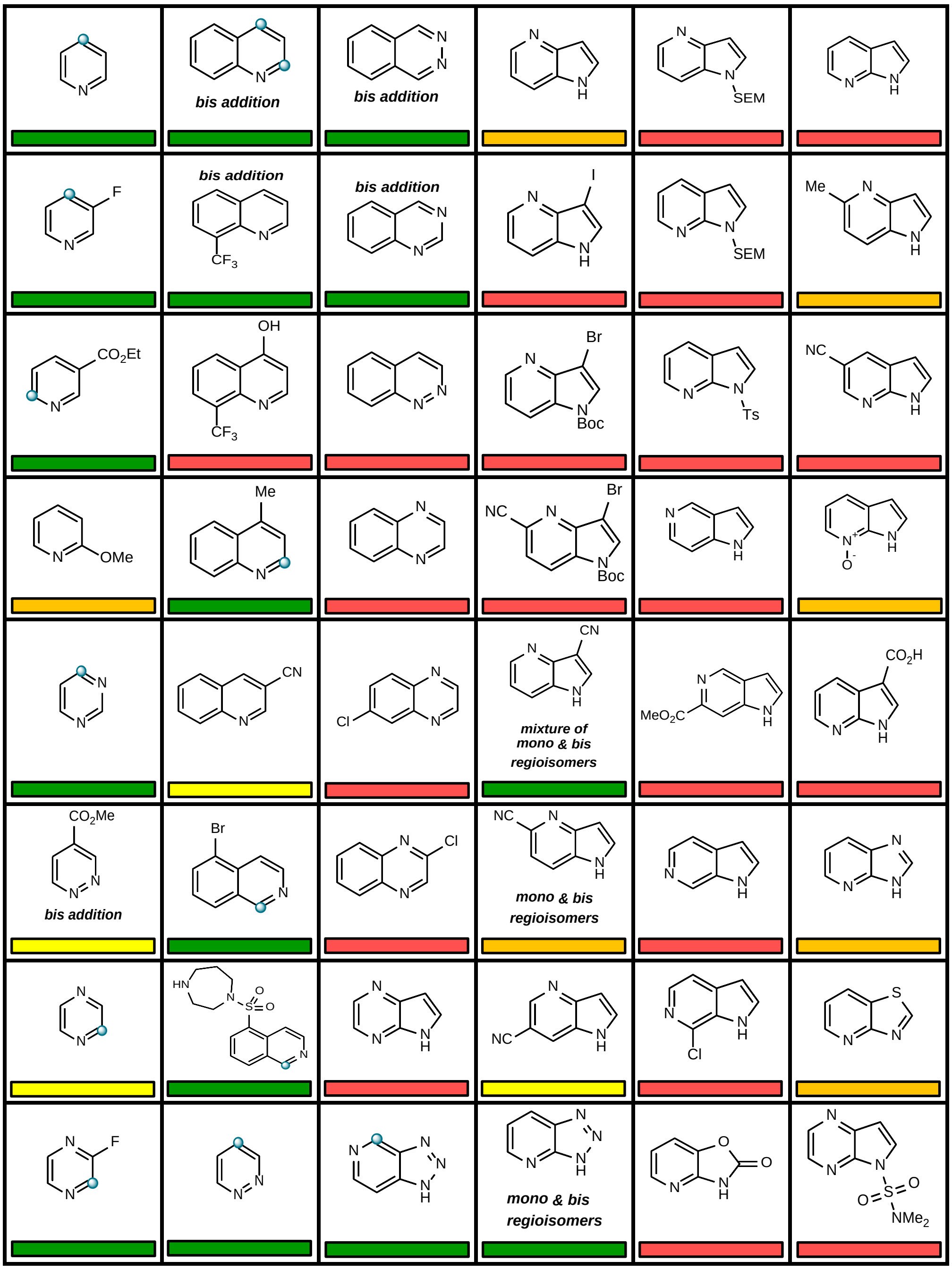

[6] Assay yield determined through HPLC conversion of starting material to product.
[7] Site of reaction observed on fully characterized product denoted by ( ).

Micromolar scale reactions in batch: General procedure

For solid substrates

{Ir[dFCF$_3$(ppy)$_2$]dtbbpy}PF$_6$ (0.02 equiv, 0.01 mmol), heteroarene (0.5 mmol, 1.0 equiv), amine (*stated amount*), $(NH_4)_2S_2O_8$ (2.0 mmol, 4.0 equiv) and TsOH.H_2O (1.0 mmol, 2.0 equiv) were weighed into a 30 mL crimp top glass vial equipped with a magnetic stir bar. The vial was sealed, degassed DMSO added (5 mL) and the headspace of the vial purged with a positive flow of nitrogen. Following this, the reaction vial was sonicated to ensure all reagents were in solution. Following this, the solution was sparged with N_2 for 10 mins then placed in a Evoluchem^{TM} PhotoRedOx box (equipped with one Evoluchem^{TM} 455 nm 18W LED) on a stirrer plate and irradiated for 16 hours with stirring at 500 rpm and internal fan cooling switched on.

For liquid or volatile substrates

{Ir[dFCF$_3$(ppy)$_2$]dtbbpy}PF$_6$ (0.02 equiv, 0.01 mmol), $(NH_4)_2S_2O_8$ (2.0 mmol, 4.0 equiv) and TsOH.H_2O (1.0 mmol, 2.0 equiv) were weighed into a 30 mL crimp top glass vial equipped with a magnetic stir bar. The vial was sealed, degassed DMSO added (5 mL) and the reaction vial was sonicated to ensure all reagents were in solution. Following this, the solution was sparged with N_2 for 10 mins and amine (2.5 mmol, 5.0 equiv) and heteroarene (0.5 mmol, 1.0 equiv) added as a solution in degassed DMSO. The vial was then placed in a Hepatochem PhotoRedOx box on a stirrer plate and irradiated for 16 hours with stirring at 500 rpm.

Workup and Purification Procedure

The reaction mixture was basified with sat. $NaHCO_3$ (20 mL) and iPrOAc (25 mL) added, the phases were separated, the aqueous layer extracted with iPrOAc (2 x 25 mL) and the combined organic layers were washed with a small amount of cold water (15 mL), dried ($MgSO_4$), filtered and concentrated *in vacuo*. The resulting crude product was purified by automated flash column chromatography on a Biotage Isolera system using the stated eluent systems.

N.B. Reported yields of isolated compounds are unoptimized, with the same conditions used for every compound. The exception being for amines which were found to have increased reactivity, consequently the stoichiometry was lowered to 1.5 or 3.0 equivalents.

Characterization Data

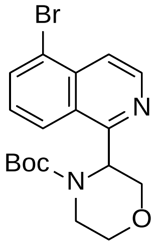

Chemical Formula: $C_{18}H_{21}BrN_2O_3$
Molecular Weight: 393.28
SMILES: CC(C)(C)OC(=O)N1CCOCC1c2nccc3c(Br)cccc23

(±)-tert-Butyl 3-(5-bromoisoquinolin-1-yl)morpholine-4-carboxylate(±)-5a: Prepared according to the general procedure using 5-bromoisoquinoline (104 mg, 1.0 equiv, 0.5 mmol), tert-Butyl morpholine-4-carboxylate (468 mg, 5.0 equiv, 2.5 mmol), {Ir[dFCF$_3$(ppy)$_2$]dtbbpy}PF$_6$ (11.2 mg, 2 mol %, 0.01 mmol), $(NH_4)_2S_2O_8$ (456 mg, 4 equiv, 2.0 mmol), TsOH.H_2O (190 mg, 2.0 equiv, 1.0 mmol). The crude product was purified by column chromatography (25g SNAP cartridge, 5–25–100% EtOAc/Petrol v/v) to afford **(±)-5a** as an off-white solid (163 mg, 83%).

^{1}H NMR (400 MHz, Chloroform-*d*) δ 8.59 (d, *J* = 5.9 Hz, 1H), 8.08 (d, *J* = 8.5 Hz, 1H), 7.94 (td, *J* = 7.5, 0.8 Hz, 2H), 7.43 (dd, *J* = 8.6, 7.5 Hz, 1H), 5.81 (br s, 1H), 4.33 (d, *J* = 11.7 Hz, 1H), 4.10 – 3.96 (m, 3H), 3.81 (ddd, *J* = 12.8, 3.5, 1.4 Hz, 1H), 3.65 (td, *J* = 11.5, 3.3 Hz, 1H), 1.29 (s, 9H);

^{13}C NMR (101 MHz, Chloroform-*d*) δ 159.54, 156.00, 142.92, 135.71, 133.65, 127.60, 127.04, 123.66, 123.01, 119.01, 80.29, 69.43, 67.17, 53.12, 42.39, 28.34 (3C).

HRMS (ESI-QTOF): m/z $[M+H]^+$ Calcd for $C_{18}H_{21}BrN_2O_3$ 393.0808; Found 393.0808. Δ = 0.02 ppm.

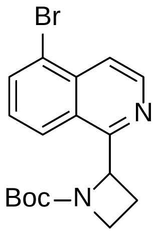

Chemical Formula: $C_{17}H_{19}BrN_2O_2$
Molecular Weight: 363.26
SMILES: CC(C)(C)OC(=O)N1CCC1c2nccc3c(Br)cccc23

(±)-tert-Butyl 2-(5-bromoisoquinolin-1-yl)azetidine-1-carboxylate (±)-5b: Prepared following the general procedure outlined above using 5-bromoisoquinoline (104 mg , 1.0 equiv, 0.5 mmol), tert-Butyl azetidine-1-carboxylate (118 mg, 1.5 equiv, 0.75 mmol), {Ir[dFCF$_3$(ppy)$_2$]dtbbpy}PF$_6$ (11.2 mg, 2 mol %, 0.01 mmol), $(NH_4)_2S_2O_8$ (456 mg, 4 equiv, 2.0 mmol), TsOH.H_2O (190 mg, 2.0 equiv, 1.0 mmol). The crude product was purified by column chromatography (25g SNAP cartridge, 0–30% EtOAc/Petrol v/v) to afford **(±)-5b** as a pale-yellow oil (106 mg, 58%).

^{1}H NMR (400 MHz, Chloroform-*d*): δ 8.68 (d, *J* = 5.9 Hz, 1H), 8.19 (dt, *J* = 8.7, 1.1 Hz, 1H), 7.97 (dd, *J* = 2.2, 1.0 Hz, 1H), 7.95 (dd, *J* = 3.8, 1.0 Hz, 1H), 7.43 (dd, *J* = 8.5, 7.5 Hz, 1H), 6.07 (dd, *J* = 8.8, 5.7 Hz, 1H), 4.23 (td, *J* = 8.7, 5.8 Hz, 1H), 4.10 – 4.04 (m, 1H), 2.72 (dtd, *J* = 11.0, 8.9, 5.8 Hz, 1H), 2.42 (ddt, *J* = 11.0, 9.1, 6.0 Hz, 1H), 1.19 (s, 9H).

^{13}C NMR (101 MHz, Chloroform-*d*): δ 159.43, 156.47, 143.70, 135.62, 133.83, 127.57, 127.16, 123.96, 122.59, 119.33, 79.62, 61.33, 47.44, 28.33 (3C), 23.75.

HRMS (ESI-QTOF): m/z $[M+H]^+$ Calcd for $C_{17}H_{19}BrN_2O_2$ 363.0703; Found 363.0702. Δ = -0.26 ppm.

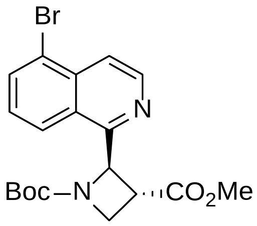

Chemical Formula: $C_{19}H_{21}BrN_2O_4$
Molecular Weight: 421.29
SMILES: COC(=O)C1CN(C1c2nccc3c(Br)cccc23)C(=O)OC(C)(C)C

(±)-*trans*-1-tert-Butyl 3-methyl 2-(5-bromoisoquinolin-1-yl)azetidine-1,3-dicarboxylate (±)-5c: Prepared following the general procedure outlined above using 5-bromoisoquinoline (104 mg, 1.0 equiv, 0.5 mmol), 1-tert-Butyl 3-methyl azetidine-1,3-dicarboxylate (538 mg, 5.0 equiv, 2.5 mmol), {Ir[dFCF$_3$(ppy)$_2$]dtbbpy}PF$_6$ (11.2 mg, 2 mol %, 0.01 mmol), $(NH_4)_2S_2O_8$ (456 mg, 4 equiv, 2.0 mmol), TsOH.H_2O (190 mg, 2.0 equiv, 1.0 mmol). The crude product was purified by column chromatography (50g SNAP cartridge, 0–40% EtOAc/Petrol v/v) to afford **(±)-5c** as a pale-yellow oil (63.5 mg, 30%, >20:1 dr).

^{1}H NMR (400 MHz, Chloroform-d): δ 8.70 (d, *J* = 5.9 Hz, 1H), 8.36 (d, *J* = 8.6 Hz, 1H), 8.0 (dd, *J* = 5.9, 1.0 Hz, 1H), 7.97 (dd, *J* = 7.5, 0.9 Hz, 1H), 7.47 (dd, *J* = 8.6, 7.5 Hz, 1H), 6.27 (d, *J* = 5.5 Hz, 1H), 4.43 (t, *J* = 8.7 Hz, 1H), 4.20 (dd, *J* = 8.3, 5.9 Hz, 1H), 3.77 (s, 3H), 3.73 (dt, *J* = 9.2, 5.7 Hz, 1H) 1.43 – 0.99 (m, 9H).

^{13}C NMR (101 MHz, Chloroform-*d*): δ 172.74, 157.46, 156.10, 143.72, 135.66, 134.04, 127.88, 127.74, 124.29, 122.45, 119.98, 80.15, 62.89, 52.56, 50.14, 39.57, 28.27 (3C).

HRMS (ESI-QTOF): m/z $[M+H]^+$ Calcd for $C_{19}H_{21}BrN_2O_4$ 421.0757; Found 421.0757. Δ = -0.21 ppm.

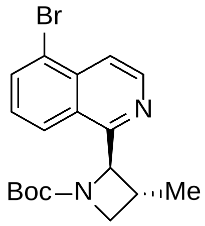

Chemical Formula: $C_{18}H_{21}BrN_2O_2$
Molecular Weight: 377.28
SMILES: CC1CN(C1c2nccc3c(Br)cccc23)C(=O)OC(C)(C)C

(±)-*trans*-tert-Butyl 2-(5-bromoisoquinolin-1-yl)-3-methylazetidine-1-carboxylate (±)-5d: Prepared following the general procedure outlined above using 5-bromoisoquinoline (104 mg, 1.0 equiv, 0.5 mmol), tert-Butyl 3-methylazetidine-1-carboxylate (538 mg, 5.0 equiv, 2.5 mmol), {Ir[dFCF$_3$(ppy)$_2$]dtbbpy}PF$_6$ (11.2 mg, 2 mol %, 0.01 mmol), $(NH_4)_2S_2O_8$ (456 mg, 4 equiv, 2.0 mmol), TsOH.H_2O (190 mg, 2.0 equiv, 1.0 mmol). The crude product was purified by reverse phase column chromatography (50g C18 Ultra cartridge, 5–50–95% MeCN/H_2O v/v with 0.1% HCO_2H modifier) to afford **(±)-5d** as a pale-yellow oil (101.6 mg, 54%, >20:1 dr).

^{1}H NMR (400 MHz, Chloroform-d): δ 8.70 (d, *J* = 5.9 Hz, 1H), 8.27 (d, *J* = 8.5 Hz, 1H), 8.04-7.94 (m, 2H), 7.47 (dd, *J* = 8.6, 7.4 Hz, 1H), 5.63 (d, *J* = 5.6 Hz, 1H), 4.37 (t, *J* = 8.1 Hz, 1H), 3.68 (dd, J = 8.1, 5.5 Hz, 1H) 1.46 (d, *J* = 7.1 Hz, 3H), 1.22 (br s, 9H).

^{13}C NMR (101 MHz, Chloroform-d): δ159.08, 156.59, 143.76, 135.68, 133.89, 127.55, 127.52, 123.98, 122.63, 119.24, 79.56, 68.67, 54.16, 32.82, 28.34 (3C), 19.29.

HRMS (ESI-QTOF): m/z $[M+H]^+$ Calcd for $C_{18}H_{21}BrN_2O_2$ 377.0859; Found 377.0858. Δ = -0.25 ppm.

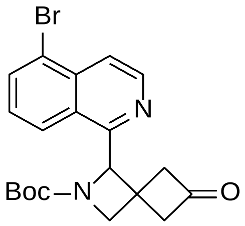

Chemical Formula: $C_{20}H_{21}BrN_2O_3$
Molecular Weight: 417.30
SMILES: CC(C)(C)OC(=O)N2CC1(CC(=O)C1)C2c3nccc4c(Br)cccc34

(±)-tert-Butyl 1-(5-bromoisoquinolin-1-yl)-6-oxo-2-azaspiro[3.3]heptane-2-carboxylate (±)-5e: Prepared following the general procedure outlined above using 5-bromoisoquinoline (104 mg, 1.0 equiv, 0.5 mmol), tert-Butyl 6-oxo-2-azaspiro[3.3]heptane-2-carboxylate (317 mg, 3.0 equiv, 1.5 mmol), {Ir[dFCF$_3$(ppy)$_2$]dtbbpy}PF$_6$ (11.2 mg, 2 mol %, 0.01 mmol), $(NH_4)_2S_2O_8$ (456 mg, 4 equiv, 2.0 mmol), TsOH.H_2O (190 mg, 2.0 equiv, 1.0 mmol). The crude product was purified by reverse phase column chromatography (50g C18 Ultra cartridge, 5–50–95% MeCN/H_2O v/v with 0.1% HCO_2H modifier) to afford **(±)-5e** as an off-white solid (117 mg, 56%).

^{1}H NMR (400 MHz, Chloroform-*d*) δ 8.70 (d, *J* = 5.9 Hz, 1H), 8.09 (dt, *J* = 8.6, 1.1 Hz, 1H), 8.01 (dd, *J* = 2.6, 1.0 Hz, 1H), 7.99 (dd, *J* = 4.2, 1.0 Hz, 1H), 7.46 (dd, *J* = 8.6, 7.5 Hz, 1H), 6.16 (s, 1H), 4.49 (d, *J* = 8.3 Hz, 1H), 4.26 (d, *J* = 8.2 Hz, 1H), 3.60 (ddd, *J* = 18.3, 4.6, 2.4 Hz, 1H), 3.47 (ddd, *J* = 18.3, 5.2, 2.4 Hz, 1H), 2.88 (ddd, *J* = 18.8, 5.2, 2.4 Hz, 1H), 2.36 (ddd, *J* = 18.7, 4.7, 2.4 Hz, 1H), 1.32 – 0.96 (m, 9H).

^{13}C NMR (101 MHz, Chloroform-*d*) δ 203.98, 156.57, 155.86, 143.72, 135.59, 134.08, 128.08, 127.93, 122.93, 122.93, 122.91, 119.55, 79.98, 69.44, 60.08, 54.33, 33.79, 28.16 (3C).

HRMS (ESI-QTOF): m/z $[M+H]^+$ Calcd for $C_{20}H_{21}BrN_2O_3$ 417.0809; Found 417.0809. Δ = -0.22 ppm.

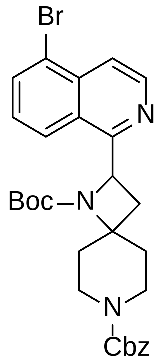

Chemical Formula: $C_{29}H_{32}BrN_3O_4$
Molecular Weight: 566.50
SMILES: C(C)(C)OC(=O)N1C(CC21CCN(CC2)C(=O)OCc3ccccc3)c4nccc5c(Br)cccc45

(±)-7-benzyl 1-tert-Butyl 2-(5-bromoisoquinolin-1-yl)-1,7-diazaspiro[3.5]nonane-1,7-dicarboxylate (±)-5f: Prepared following the general procedure outlined above using 5-bromoisoquinoline (104 mg, 1.0 equiv, 0.5 mmol), 7-benzyl 1-tert-Butyl 1,7-diazaspiro[3.5]nonane-1,7-dicarboxylate (541 mg, 3.0 equiv, 1.5 mmol), {Ir[dFCF$_3$(ppy)$_2$]dtbbpy}PF$_6$ (11.2 mg, 2 mol %, 0.01 mmol), $(NH_4)_2S_2O_8$ (456 mg, 4 equiv, 2.0 mmol), TsOH.H_2O (190 mg, 2.0 equiv, 1.0 mmol). The crude product was purified by reverse phase column chromatography (25g SNAP cartridge, 15–40% EtOAc/Petrol v/v) to afford **(±)-5f** as a yellow oil (152 mg, 65%).

^{1}H NMR (400 MHz, Chloroform-*d*): (mixture of rotamers): δ 8.66 (d, *J* = 6.7 Hz, 1H), 8.22 – 8.13 (m, 1H), 7.95 (s, 2H), 7.43 (dd, *J* = 8.5, 7.5 Hz, 1H), 7.39 – 7.27 (m, 5H), 6.04 (dd, *J* = 8.8, 5.9 Hz, 1H), 5.15 (s, 2H), 4.23 (br s, 2H), 2.89 (br s, 2H), 2.60 – 2.42 (m, 2H), 2.40 – 2.20 (m, 3H), 1.93 – 1.79 (m, 1H), 1.48 – 1.37 (m, 5H, C(C*H*$_3$)$_3$), 1.04 (s, 4H, C(C*H*$_3$)$_3$).

^{13}C NMR (101 MHz, Chloroform-*d*) (mixture of rotamers): δ 159.34, 158.96, 155.43, 155.29, 143.56, 136.88, 135.56, 133.78, 128.57 (2C), 128.07, 127.91 (2C), 127.55, 127.21, 123.90, 122.56, 119.34, 119.33, 80.21, 79.39, 67.23, 65.40, 64.97, 55.99, 54.96, 40.95 (2C), 35.66, 35.25, 34.75, 34.64, 28.68 (rotamer singlet, 3C), 28.24 (rotamer singlet, 3C).

HRMS (ESI-QTOF): m/z $[M+H]^+$ Calcd for $C_{29}H_{32}BrN_3O_4$ 566.1649; Found 566.1649. Δ = -0.02 ppm.

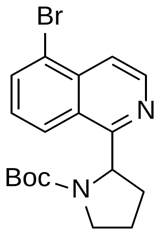

Chemical Formula: $C_{18}H_{21}BrN_2O_2$
Molecular Weight: 377.28
SMILES: CC(C)(C)OC(=O)N1CCCC1c2nccc3c(Br)cccc23

(±)-tert-Butyl 2-(5-bromoisoquinolin-1-yl)pyrrolidine-1-carboxylate (±)-5g: Prepared following the general procedure outlined above using 5-bromoisoquinoline (104 mg, 1.0 equiv, 0.5 mmol), tert-Butyl pyrrolidine-1-carboxylate (256.9 mg, 3.0 equiv, 1.5 mmol), {Ir[dFCF$_3$(ppy)$_2$]dtbbpy}PF$_6$ (11.2 mg, 2 mol %, 0.01 mmol), $(NH_4)_2S_2O_8$ (456 mg, 4 equiv, 2.0 mmol), TsOH.H_2O (190 mg, 2.0 equiv, 1.0 mmol). The crude product was purified by column chromatography (25g SNAP cartridge, 5–30% EtOAc/Petrol v/v) to afford **(±)-5g** as an off-white solid (124.4 mg, 66%).

^{1}H NMR (400 MHz, Chloroform-*d*): (mixture of rotamers) δ 8.49 (dd, *J* = 9.6, 5.8 Hz, 1H), 8.13 (dd, *J* = 13.5, 8.4 Hz, 1H), 7.83 (td, *J* = 16.8, 15.7, 6.6 Hz, 2H), 7.35 (q, *J* = 8.5 Hz, 1H), 5.77 (dd, *J* = 8.7, 3.0 Hz, 0.40H), 5.60 (dd, *J* = 8.1, 4.5 Hz, 0.60H), 3.86 – 3.72 (m, 1H), 3.69 – 3.50 (m, 1H), 2.40 (ddd, *J* = 21.7, 10.7, 6.4 Hz, 1H), 2.09 – 1.81 (m, 3H), 1.38 (s, 3.5H, C(CH_3)$_3$), 0.89 (s, 5.5H, C(CH_3)$_3$).

^{13}C NMR (101 MHz, Chloroform-*d*): (mixture of rotamers) δ 162.57, 161.62, 154.47, 154.10, 143.11, 143.03, 135.41, 135.21, 133.46, 133.34, 127.20, 126.69, 126.55, 123.91, 123.65, 122.41, 118.47, 118.27, 79.11, 78.68, 59.06, 58.47, 47.18, 46.94, 33.84, 32.82, 28.42 (rotamer singlet, 3C), 27.91 (rotamer singlet, 3C), 23.94, 23.51.

HRMS (ESI-QTOF): m/z [M+H]$^+$ Calcd for $C_{18}H_{21}BrN_2O_2$ 377.0859; Found 377.0857. Δ = -0.55 ppm.

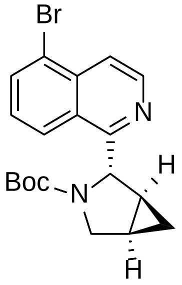

Chemical Formula: $C_{19}H_{21}BrN_2O_2$
Molecular Weight: 389.29
SMILES: CC(C)(C)OC(=O)N1CC2CC2C1c3nccc4c(Br)cccc34

(±)-*trans*-tert-Butyl 2-(5-bromoisoquinolin-1-yl)-3-azabicyclo[3.1.0]hexane-3-carboxylate (±)-5h: Prepared following the general procedure outlined above using 5-bromoisoquinoline (104 mg, 1.0 equiv, 0.5 mmol), tert-Butyl 3-azabicyclo[3.1.0]hexane-3-carboxylate (194.6 mg, 5.0 equiv, 2.5 mmol), {Ir[dFCF$_3$(ppy)$_2$]dtbbpy}PF$_6$ (11.2 mg, 2 mol %, 0.01 mmol), $(NH_4)_2S_2O_8$ (456 mg, 4 equiv, 2.0 mmol), TsOH.H_2O (190 mg, 2.0 equiv, 1.0 mmol). The crude product was purified (25g SNAP cartridge, 0–30% EtOAc/Petrol v/v) to afford **(±)-5h** as a pale-yellow oil (66 mg, 34%, >20:1 dr).

^{1}H NMR (400 MHz, Chloroform-*d*): (mixture of rotamers) δ 8.58 (dd, *J* = 9.6, 5.9 Hz, 1H), 8.34 (dd, *J* = 34.9, 8.5 Hz, 1H), 8.03 – 7.88 (m, 2H), 7.48 (dt, *J* = 8.5, 7.1 Hz, 1H), 5.83 (s, 0.40H), 5.67 (s, 0.60H), 4.03 (dd, *J* = 10.0, 4.2 Hz, 0.60H), 3.96 (dd, *J* = 10.1, 4.1 Hz, 0.40H), 3.77 (dd, *J* = 12.8, 10.1 Hz, 1H), 1.71 – 1.49 (m, 2H), 1.43 (s, 4H, C(C*H*$_3$)$_3$), 1.05 (s, 5H, C(C*H*$_3$)$_3$), 0.87 – 0.78 (m, 1H), 0.60 (dq, J = 24.7, 4.3 Hz, 1H).

^{13}C NMR (101 MHz, Chloroform-*d*): (mixture of rotamers) δ 161.87, 161.17, 155.52, 155.30, 143.62, 143.41, 135.62, 135.39, 133.71, 133.66, 127.52, 127.47, 127.25, 126.84, 124.43, 124.02, 122.63, 122.50, 118.95, 118.71, 79.66, 79.29, 61.18, 60.74, 49.53, 49.47, 28.56 (rotamer singlet, 3C), 28.21 (rotamer singlet, 3C), 23.02, 22.16, 16.55, 16.08, 10.34, 9.87.

HRMS (ESI-QTOF): m/z $[M+H]^+$ Calcd for $C_{19}H_{21}BrN_2O_2$ 389.0859; Found 389.0858. Δ = -0.41 ppm.

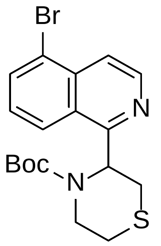

Chemical Formula: $C_{18}H_{21}BrN_2O_2S$
Molecular Weight: 409.34
SMILES: CC(C)(C)OC(=O)N1CCSCC1c2nccc3c(Br)cccc23

(±)-tert-Butyl 3-(5-bromoisoquinolin-1-yl)thiomorpholine-4-carboxylate (±)-5i: Prepared following the general procedure outlined above using 5-bromoisoquinoline (104 mg, 1.0 equiv, 0.5 mmol), tert-Butyl thiomorpholine-4-carboxylate (508 mg, 2.5 mmol), {Ir[dFCF$_3$(ppy)$_2$]dtbbpy}PF$_6$ (11.2 mg, 2 mol %, 0.01 mmol), $(NH_4)_2S_2O_8$ (456 mg, 4 equiv, 2.0 mmol), TsOH.H_2O (190 mg, 2.0 equiv, 1.0 mmol). The crude product was purified by column chromatography (25g SNAP cartridge, 0–30% EtOAc/Petrol v/v) to afford **(±)-5i** as a pale yellow solid (83 mg, 41%).

^{1}H NMR (400 MHz, Chloroform-*d*): δ 8.60 (d, *J* = 5.9 Hz, 1H), 8.33 (d, *J* = 8.5 Hz, 1H), 8.00 – 7.93 (m, 2H), 7.49 (dd, *J* = 8.5, 7.5 Hz, 1H), 4.91 – 4.37 (m, 3H), 3.73 – 3.45 (m, 1H), 3.20 (td, *J* = 12.4, 11.8, 6.6 Hz, 1H), 3.10 – 2.98 (m, 1H), 2.70 (d, *J* = 13.3 Hz, 1H), 1.47 (s, 9H).

^{13}C NMR (101 MHz, Chloroform-*d*): δ 158.11, 154.91, 143.37, 135.65, 134.04, 127.91, 127.49, 124.51, 122.77, 119.55, 80.42, 51.69, 45.55, 43.27, 28.93, 28.60 (3C).

HRMS (ESI-QTOF): m/z $[M+H]^+$ Calcd for $C_{18}H_{21}BrN_2O_2S$ 409.0580; Found 409.0579. Δ = -0.42 ppm.

N.B. This reaction is low-moderate yielding due to the turbidity of the reaction mixture. The amine is poorly soluble in DMSO, even following prolonged sonication. The addition of $CHCl_3$ and CH_2Cl_2 as co-solvents improved solubility of the N-Boc thiomorpholine, resulting in clear solutions, however a detrimental effect on yield was observed with the addition of these apolar solvents. Furthermore, decreasing the concentration of the reaction to 0.05 M (DMSO) did not afford an improved yield.

(±)-tert-Butyl 4-acetyl-2-(5-bromoisoquinolin-1-yl)piperazine-1-carboxylate (±)-5j-major & (±)-tert-Butyl 4-acetyl-3-(5-bromoisoquinolin-1-yl)piperazine-1-carboxylate (±)-5j-minor.

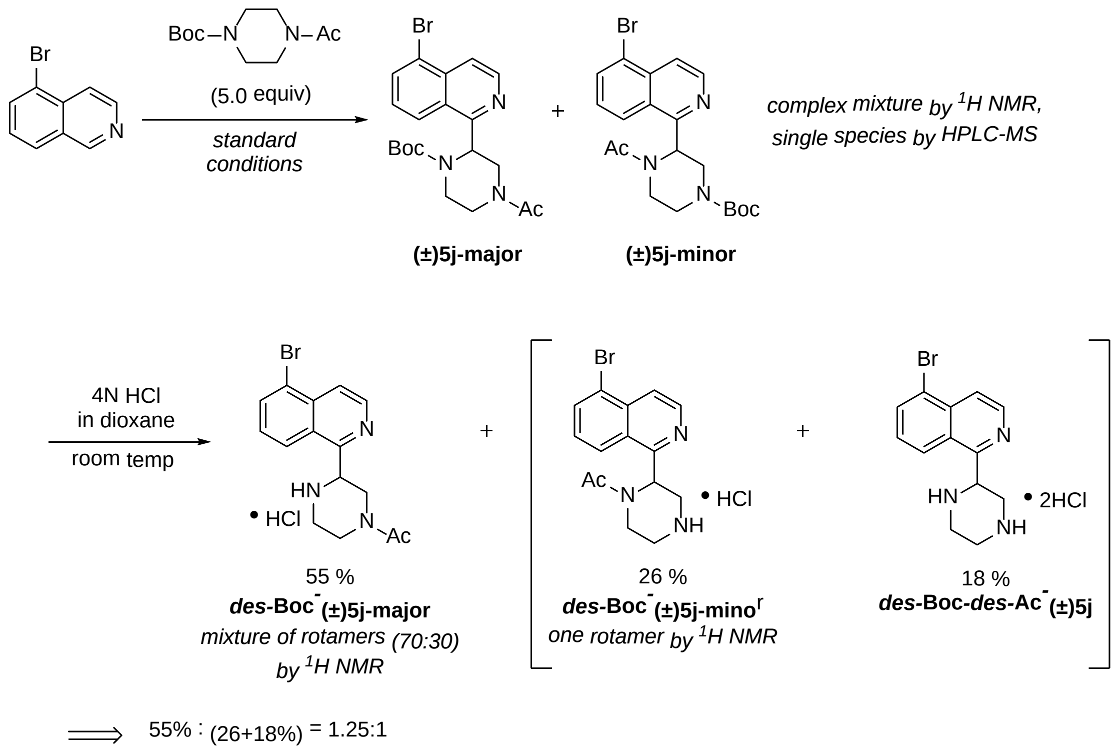

Prepared following the general procedure outlined above using 5-bromoisoquinoline (104 mg, 1.0 equiv, 0.5 mmol), tert-Butyl 4-acetylpiperazine-1-carboxylate (571 mg, 5.0 equiv, 2.5 mmol), {Ir[dFCF$_3$(ppy)$_2$]dtbbpy}PF$_6$ (11.2 mg, 2 mol %, 0.01 mmol), $(NH_4)_2S_2O_8$ (456 mg, 4 equiv, 2.0 mmol), TsOH.H_2O (190 mg, 2.0 equiv, 1.0 mmol). The crude product was purified (50g C18 Ultra cartridge, 5–50–95% MeCN/H_2O v/v with 0.1% HCO_2H modifier) to afford a yellow solid (137 mg, 63%) which showed 1 species by HPLC-MS corresponding to the mass of **(±)-5j**, however a complex mixture was observed by ^{1}H NMR (Figure SI-10). The yellow solid was dissolved in dioxane (2 mL) and 4N HCl in dioxane (4 mL) added and the resulting yellow solution was stirred at room temperature for 1 hour. After this time the solution was concentrated *in vacuo* and analyzed by HPLC-MS to show two products with masses corresponding to ***des*-Boc-(±)5j** regioisomers (Figure SI-11). However, after ^{1}H and ^{13}C NMR analysis with 2D correlation experiments a mixture of three compounds were detected, two *des*-Boc regioisomers (55% and 26%) and a des-Boc-des-Ac compound (18%) which was confirmed by NMR analysis (see below) and detected by HRMS.

Observation of the fully deprotected ***des*-Boc-des-Ac-(±)5j** (18%) was surprising as the Boc deprotection was performed with 4N HCl at room temperature and to the best of our knowledge, there are no reports of acetamide deprotection under these conditions. We hypothesize that ***des*-Boc-des-Ac-(±)5j** arose from deprotection of ***des*-Boc-(±)5j-minor** as only one amide tautomer was observed by ^{1}H & ^{13}C NMR it seems feasible that the other ***des*-Boc-(±)5j-minor** tautomer was unstable under the Boc deprotection conditions likely caused by neighbouring group participation of the adjacent isoquinoline nitrogen.

After photochemical coupling reaction

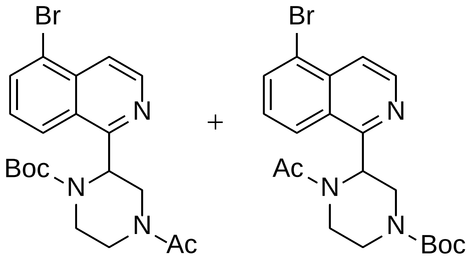

(±)5j-major **(±)5j-minor**

(±)-tert-Butyl 4-acetyl-2-(5-bromoisoquinolin-1-yl)piperazine-1-carboxylate (±)-5j-major & (±)-tert-Butyl 4-acetyl-3-(5-bromoisoquinolin-1-yl)piperazine-1-carboxylate (±)-5j-minor.

LC-MS $[M+H]^+$ = 434

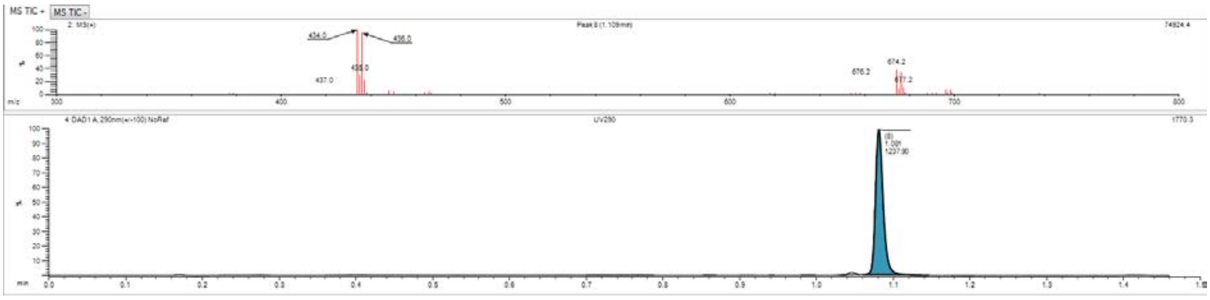

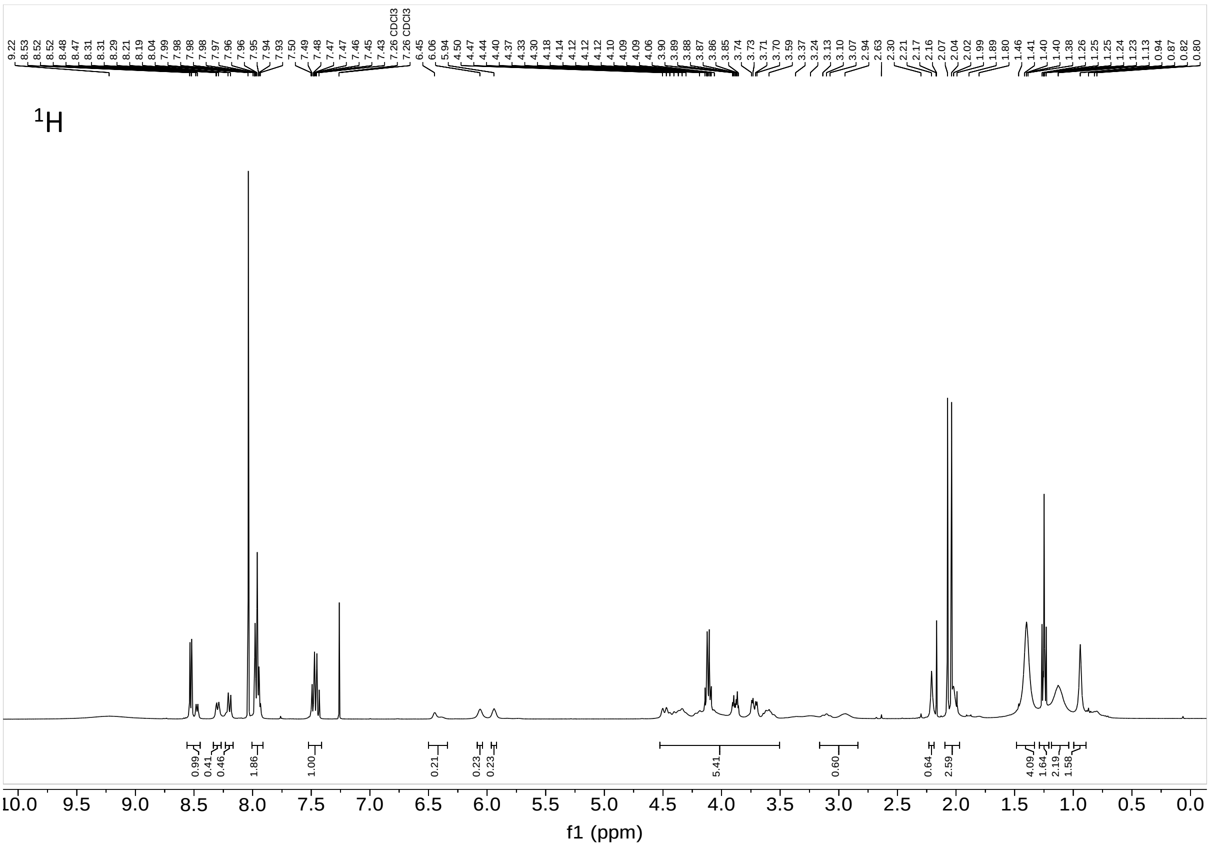

Figure SI-10: LCMS trace of crude reaction mixture of **(±)-5j** after photochemical coupling, showing one compound, however a complex mixture was observed by ^{1}H NMR.

<u>After Boc deprotection</u>

(±)-1-[3-(5-bromoisoquinolin-1-yl)piperazin-1-yl]ethan-1-one [*des*-Boc (±)-5j-major] & 1-[2-(5-bromoisoquinolin-1-yl)piperazin-1-yl]ethan-1-one [*des*-Boc (±)-5j-minor]

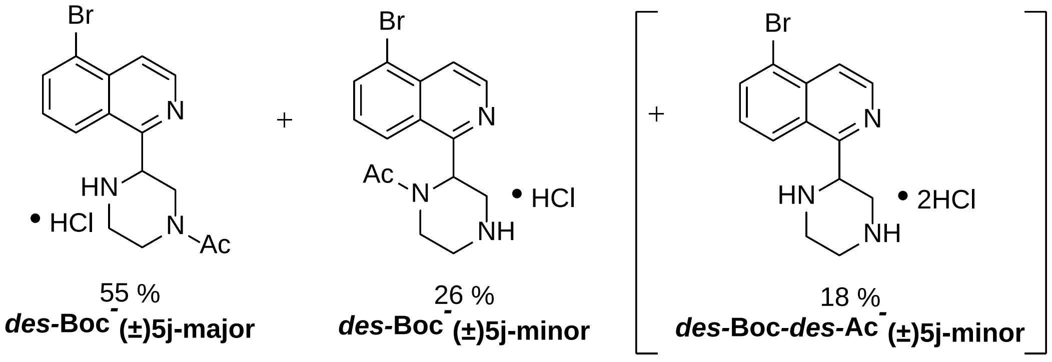

LC-MS $[M+H]+$ = 334 LC-MS $[M+H]+$ = 291

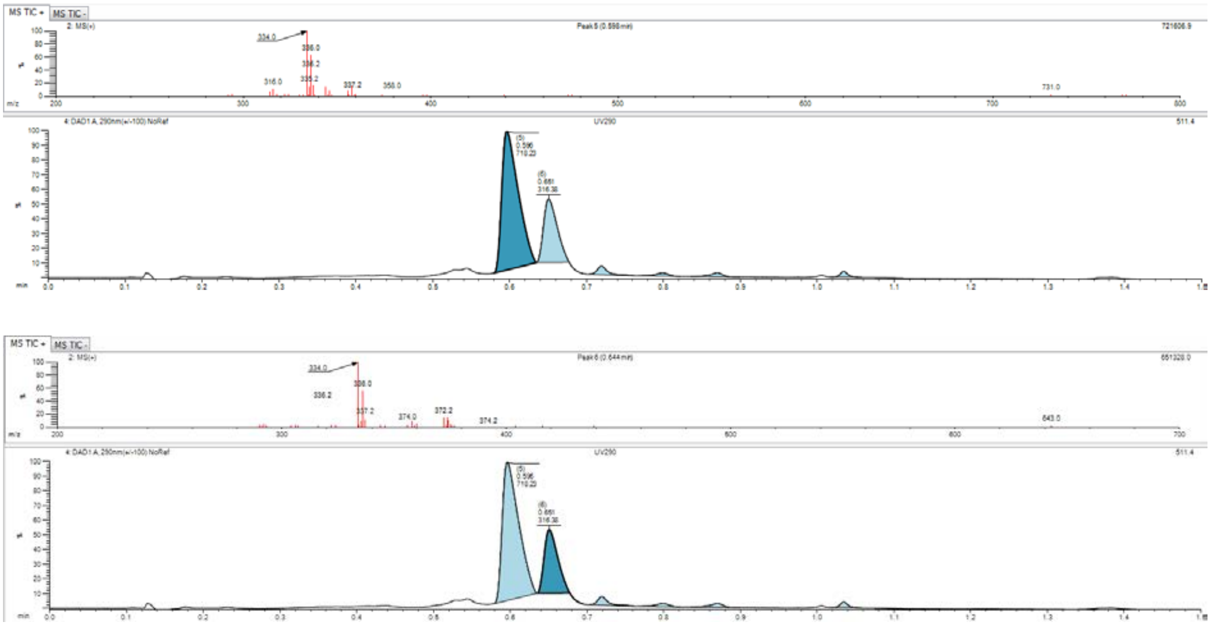

Figure SI-11: LCMS trace of crude reaction mixture of **(±)-5j** after Boc deprotection with 4N HCl at room temperature, showing two compounds, both with m/z 334.

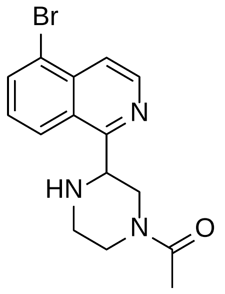

***des*-Boc^{-}(±)5j-major**
major tautomer (70%)

^{1}H NMR (400 MHz, Chloroform-*d*)/Methanol-*d*$_4$): δ 8.60 (d, *J* = 5.8 Hz, 1H), 8.16 (d, *J* = 8.3 Hz, 1H), 8.06 (d, *J* = 6.2 Hz, 1H), 8.03 (m, 1H), 7.57 (m, 1H), 5.25 (m, 1H), 5.06 (m, 1H), 4.14 (m, 1H), 3.79 (m, 1H), 3.71 (m, 1H), 3.45 (m, 1H), 2.94 (dd, *J* =14.3, 11.1 Hz, 1H), 2.23 (s, 3H).

^{13}C NMR (101 MHz, Chloroform-*d*)/Methanol-*d*$_4$): 170.1, 151.6, 142.5, 135.9, 134.9, 129.2, 125.7, 122.9, 122.3, 121.0, 55.8, 44.4, 43.5, 42.8, 21.0.

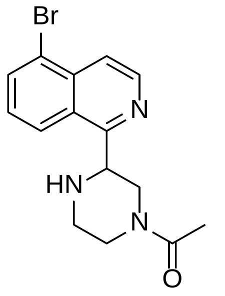

***des*-Boc^{-}(±)5j-major**
minor tautomer (30%)

^{1}H NMR (400 MHz, Chloroform-*d*)/Methanol-*d*$_4$): δ 8.62 (dd, *J* = 5.9, 1.3 Hz, 1H), 8.30 (d, *J* = 8.2 Hz, 1H), 8.09 (d, *J* = 5.9 Hz, 1H), 8.04 (m, 1H), 7.58 (m, 1H), 5.67 (d, *J* = 10.2 Hz, 1H), 4.71 (d, *J* = 14.1 Hz, 1H), 4.20 (d, *J* = 14.3 Hz, 1H), 3.66 (m, 1H), 3.52 (m, 1H), 3.42 (m, 1H), 3.29 (m, 1H), 2.14 (s, 3H).

^{13}C NMR (101 MHz, Chloroform-*d*)/Methanol-*d*$_4$): 169.5, 151.6, 142.6, 135.2, 134.9, 129.2, 125.7, 122.6, 122.4, 122.6, 121.5, 55.0, 49.0, 43.0, 37.7, 21.1.

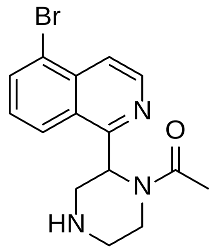

***des*-Boc-(±)5j-minor**
single tautomer observed (>99%)

^{1}H NMR (400 MHz, Chloroform-*d*)/Methanol-*d$_4$*): δ 8.58 (m, 1H), 8.41 (m, 1H), 8.14 (d, *J* = 5.8 Hz, 1H), 8.06 (m, 1H), 7.54 (m, 1H), 6.78 (d, *J* = 3.9 Hz, 1H), 3.93 (d, *J* = 12.9 Hz, 1H), 3.87 (m, 1H), 3.86 (m, 1H), 3.60 (m, 1H), 3.51 (m, 1H), 3.44 (m, 1H), 2.19 (s, 3H).

^{13}C NMR (101 MHz, Chloroform-*d*)/Methanol-*d$_4$*): 169.5, 155.9, 140.4, 135.4 (2C), 129.2, 126.8, 124.7, 122.1, 121.4, 45.6, 45.1, 39.9, 39.4, 21.3.

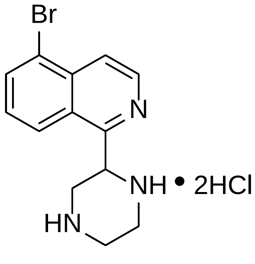

18 %
***des*-Boc-*des*-Ac-(±)5j**

from:

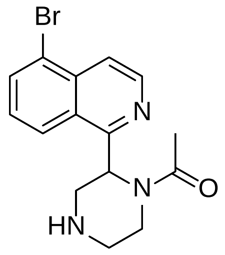

***des*-Boc-(±)5j-minor**
tautomer not observed

^{1}H NMR (400 MHz, Chloroform-*d*)/Methanol-*d$_4$*): δ 8.62 (m, 1H), 8.39 (m, 1H), 8.07 (m, 1H), 8.04 (m, 1H), 7.58 (m, 1H), 6.18 (dd, *J* = 11.9, 3.0 Hz, 1H), 3.96 (m, 2H), 3.88 (m, 1H), 3.85 (m, 1H), 3.78 (m, 1H), 3.47 (m, 1H).

^{13}C NMR (101 MHz, Chloroform-*d*)/Methanol-*d$_4$*): 150.4, 142.6, 134.9 (2C), 129.2, 125.8, 123.1, 122.4, 121.0, 52.8, 44.8, 40.3, 39.6.

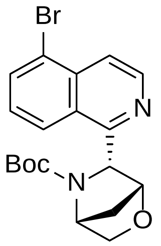

Chemical Formula: $C_{19}H_{21}BrN_2O_3$
Molecular Weight: 405.29
SMILES: CC(C)(C)OC(=O)N1C(C2CC1CO2)c3nccc4c(Br)cccc34

(±)-*trans*-tert-Butyl 6-(5-bromoisoquinolin-1-yl)-2-oxa-5-azabicyclo[2.2.1]heptane-5-carboxylate (±)-5k: Prepared following the general procedure outlined above using 5-bromoisoquinoline (104 mg, 1.0 equiv, 0.5 mmol), tert-Butyl 2-oxa-5-azabicyclo[2.2.1]heptane-5-carboxylate (194.6 mg, 5.0 equiv, 2.5 mmol), {Ir[dFCF$_3$(ppy)$_2$]dtbbpy}PF$_6$ (11.2 mg, 2 mol %, 0.01 mmol), $(NH_4)_2S_2O_8$ (456 mg, 4 equiv, 2.0 mmol), TsOH.H_2O (190 mg, 2.0 equiv, 1.0 mmol). The crude product was purified by column chromatography (25g SNAP cartridge, 0–50% EtOAc/Petrol v/v) to afford **(±)-5k** as an off-white solid (139 mg, 69%, >20:1 dr).

^{1}H NMR (400 MHz, Chloroform-*d*): (mixture of rotamers) δ 8.60 (t, *J* = 5.5 Hz, 1H), 8.29 (dd, *J* = 13.7, 8.5 Hz, 1H), 8.00 – 7.88 (m, 2H), 7.47 (q, *J* = 8.0 Hz, 1H), 5.59 (d, *J* = 46.5 Hz, 1H), 4.76 (d, *J* = 49.5 Hz, 1H), 4.58 – 4.52 (m, 1H), 4.02 (dd, *J* = 34.4, 7.4 Hz, 1H), 3.89 (td, *J* = 7.4, 1.4 Hz, 1H), 2.53 (ddd, *J* = 12.4, 9.9, 2.5 Hz, 1H), 1.68 (dd, *J* = 10.1, 3.0 Hz, 1H), 1.45 (s, 4H, C(CH_3)$_3$), 1.03 (s, 5H, C(CH_3)$_3$).

^{13}C NMR (101 MHz, Chloroform-*d*): (mixture of rotamers) δ 156.20, 155.79, 154.76, 153.77, 143.37, 143.26, 135.61, 135.37, 133.89, 127.88, 127.23, 127.14, 123.78, 123.47, 122.72, 122.64, 119.38, 119.08, 81.27, 80.78, 80.41, 79.65, 74.53, 74.44, 66.51, 65.99, 57.84, 56.37, 33.58, 32.83, 28.55 (rotamer singlet, 3C), 28.11 (rotamer singlet, 3C).

HRMS (ESI-QTOF): m/z $[M+H]^+$ Calcd for $C_{19}H_{21}BrN_2O_3$ 405.0809; Found 405.0807. Δ = -0.53 ppm.

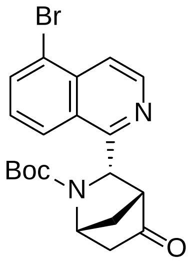

Chemical Formula: $C_{20}H_{21}BrN_2O_3$
Molecular Weight: 417.30
SMILES: CC(C)(C)OC(=O)N1C(C2CC1CC2=O)c3nccc4c(Br)cccc34

(±)-*trans*-tert-Butyl 3-(5-bromoisoquinolin-1-yl)-5-oxo-2-azabicyclo[2.2.1]heptane-2-carboxylate (±)-5l: Prepared according to the general procedure using pyridine 5-bromoisoquinoline (104 mg, 1.0 equiv, 0.5 mmol), tert-butyl 5-oxo-2-azabicyclo[2.2.1]heptane-2-carboxylate (528 mg, 5.0 equiv, 2.5 mmol), {Ir[dFCF$_3$(ppy)$_2$]dtbbpy}PF$_6$ (11.2 mg, 2 mol %, 0.01 mmol), $(NH_4)_2S_2O_8$ (456 mg, 4 equiv, 2.0 mmol), TsOH.H_2O (190 mg, 2.0 equiv, 1.0 mmol). The crude product was purified by column chromatography (25g SNAP cartridge, 0–60% EtOAc/Petrol v/v) to afford **(±)-5l** as a white solid (139 mg, 67%, >20:1 dr).

^{1}H NMR (400 MHz, Chloroform-d): δ 8.58 (t, *J* = 5.5 Hz, 1H), 8.12 (t, *J* = 9.0 Hz, 1H), 8.00 – 7.90 (m, 2H), 7.46 (q, *J* = 8.3 Hz, 1H), 5.55 (d, *J* = 50.8 Hz, 1H), 4.98 – 4.80 (d, *J* = 52.0 Hz, 1H), 2.95 – 2.90 (m, 1H), 2.71 (ddt, *J* = 11.1, 7.2, 3.5 Hz, 1H), 2.58 – 2.40 (m, 1H), 2.34 – 2.25 (m, 1H), 1.78 (dt, *J* = 10.7, 3.0 Hz, 1H), 1.44 (s, 5H), 1.01 (s, 4H).

^{13}C NMR (101 MHz, Chloroform-d): (mixture of rotamers) δ 213.58, 212.76, 156.83, 156.37, 154.34, 153.46, 143.03, 142.94, 135.73, 135.47, 133.97, 128.13, 128.09, 126.66, 126.52, 123.54, 123.19, 122.81, 122.71, 119.70, 119.41, 80.68, 79.98, 58.40, 58.09, 57.53, 57.20, 56.81, 56.01, 46.29, 46.17, 34.29, 33.65, 28.49, 28.04.

HRMS (ESI-QTOF): m/z [M+H]$^+$ Calcd for $C_{20}H_{21}BrN_2O_3$ 417.0808; Found 417.0806. Δ = -0.58 ppm.

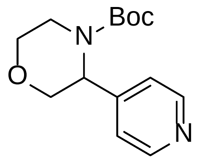

Chemical Formula: $C_{14}H_{20}N_2O_3$
Molecular Weight: 264.33
SMILES: CC(C)(C)OC(=O)N1CCOCC1c2ccncn2

(±)-tert-Butyl 3-(pyridin-4-yl)morpholine-4-carboxylate (±)-5m: Prepared according to the general procedure using pyridine (39.0 mg, 1.0 equiv, 0.5 mmol) and tert-Butyl morpholine-4-carboxylate (468 mg, 5.0 equiv, 2.5 mmol), {Ir[dFCF$_3$(ppy)$_2$]dtbbpy}PF$_6$ (11.2 mg, 2 mol %, 0.01 mmol), $(NH_4)_2S_2O_8$ (456 mg, 4 equiv, 2.0 mmol), TsOH.H_2O (190 mg, 2.0 equiv, 1.0 mmol). The crude product was purified by column chromatography (25g SNAP cartridge, 15–100% EtOAc/Petrol v/v) to afford **(±)-5m** pale-yellow oil (139 mg, 69%, >20:1 dr).

^{1}H NMR (400 MHz, Chloroform-*d*): δ 8.60 – 8.55 (m, 2H), 7.33 (ddd, *J* = 4.5, 1.7, 0.8 Hz, 2H), 5.05 (s, 1H), 4.32 (d, *J* = 12.1 Hz, 1H), 3.89 – 3.81 (m, 3H), 3.58 (ddd, *J* = 12.0, 11.3, 3.0 Hz, 1H), 3.07 (ddd, *J* = 13.7, 12.1, 3.7 Hz, 1H), 1.47 (s, 9H).

^{13}C NMR (101 MHz, Chloroform-*d*): δ 154.90, 150.14 (2C), 148.47, 122.74 (2C), 80.96, 68.58, 67.07, 52.87, 40.22, 28.47 (3C).

HRMS (ESI-QTOF): m/z $[M+H]^+$ Calcd for $C_{14}H_{20}N_2O_3$ 265.1547; Found 265.1547. Δ = 0.27 ppm.

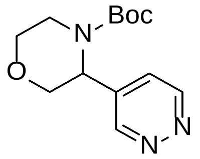

Chemical Formula: $C_{13}H_{19}N_3O_3$
Molecular Weight: 265.31
SMILES: CC(C)(C)OC(=O)N1CCOCC1c2ccncc2

(±)-tert-Butyl 3-(pyridazin-4-yl)morpholine-4-carboxylate (±)-5n: Prepared according to the general procedure using pyridazine (40.1 mg, 1.0 equiv, 0.5 mmol), tert-Butyl morpholine-4-carboxylate (468 mg, 5.0 equiv, 2.5 mmol), {Ir[dFCF$_3$(ppy)$_2$]dtbbpy}PF$_6$ (11.2 mg, 2 mol %, 0.01 mmol), $(NH_4)_2S_2O_8$ (456 mg, 4 equiv, 2.0 mmol), TsOH.H_2O (190 mg, 2.0 equiv, 1.0 mmol). The crude product was purified by column chromatography (25g SNAP cartridge, 15–80% EtOAc/Petrol v/v) to afford **(±)-5n** as a pale yellow solid (56 mg, 42%).

^{1}H NMR (400 MHz, Chloroform-*d*) δ 9.26 (dd, *J* = 2.8, 1.3 Hz, 1H), 9.13 (dd, *J* = 5.4, 1.2 Hz, 1H), 7.56 (ddd, *J* = 5.4, 2.5, 0.9 Hz, 1H), 5.07 (s, 1H), 4.28 (d, *J* = 12.3 Hz, 1H), 3.92 – 3.84 (m, 2H), 3.84 – 3.76 (m, 1H), 3.56 (dd, *J* = 11.7, 3.0 Hz, 1H), 2.97 (ddd, *J* = 13.8, 12.1, 3.8 Hz, 1H), 1.45 (s, 9H).

^{13}C NMR (101 MHz, Chloroform-d) δ 154.55, 151.53, 151.20, 138.90, 125.34, 81.47, 68.00, 66.98, 50.92, 40.07, 28.39 (3C).

HRMS (ESI-QTOF): m/z $[M+H]^+$ Calcd for $C_{13}H_{19}N_3O_3$ 266.1499; Found 265.1502. Δ = 1.07 ppm.

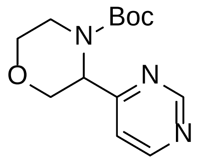

Chemical Formula: $C_{13}H_{19}N_3O_3$
Molecular Weight: 265.31
SMILES: CC(C)(C)OC(=O)N1CCOCC1c2ccncc2

(±)-tert-Butyl 3-(pyrimidin-4-yl)morpholine-4-carboxylate (±)-5o: Prepared according to the general procedure using pyrimidine (40.1 mg, 1.0 equiv, 0.5 mmol), tert-Butyl morpholine-4-carboxylate (468 mg, 5.0 equiv, 2.5 mmol), {Ir[dFCF$_3$(ppy)$_2$]dtbbpy}PF$_6$ (11.2 mg, 2 mol %, 0.01 mmol), $(NH_4)_2S_2O_8$ (456 mg, 4 equiv, 2.0 mmol), TsOH.H_2O (190 mg, 2.0 equiv, 1.0 mmol). The crude product was purified by column chromatography (25g SNAP cartridge, 15–80% EtOAc/Petrol v/v) to afford **(±)-5o** as a pale yellow solid (107 mg, 81%)

^{1}H NMR (400 MHz, Chloroform-*d*) δ 9.21 (d, *J* = 1.4 Hz, 1H), 8.70 (d, *J* = 5.3 Hz, 1H), 7.19 (ddd, *J* = 5.3, 1.5, 0.9 Hz, 1H), 5.03 (br s, 1H), 4.71 (d, *J* = 11.7 Hz, 1H), 3.94 – 3.76 (m, 3H), 3.57 (td, *J* = 11.7, 3.1 Hz, 1H), 3.23 (m, 1H), 1.45 (s, 9H).

^{13}C NMR (101 MHz, Chloroform-*d*) δ 167.74, 159.02, 157.36, 155.38, 118.67, 81.04, 68.37, 66.79, 55.63, 40.92, 28.41 (3C).

HRMS (ESI-QTOF): m/z $[M+H]^+$ Calcd for $C_{13}H_{19}N_3O_3$ 266.1499; Found 265.1501. Δ = 0.51 ppm.

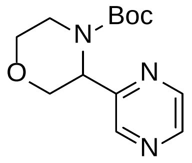

Chemical Formula: $C_{13}H_{19}N_3O_3$
Molecular Weight: 265.31
SMILES: CC(OC(N1CCOCC1C2=NC=CN=C2)=O)(C)C

(±)-tert-Butyl 3-(pyrazin-2-yl)morpholine-4-carboxylate (±)-5p: Prepared according to the general procedure using pyrazine (40.1 mg, 1.0 equiv, 0.5 mmol), tert-Butyl morpholine-4-carboxylate (468 mg, 5.0 equiv, 2.5 mmol), {Ir[dFCF$_3$(ppy)$_2$]dtbbpy}PF$_6$ (11.2 mg, 2 mol %, 0.01 mmol), $(NH_4)_2S_2O_8$ (456 mg, 4 equiv, 2.0 mmol), TsOH.H_2O (190 mg, 2.0 equiv, 1.0 mmol). The crude product was purified by column chromatography (25g SNAP cartridge, 15–100% EtOAc/Petrol v/v) to afford **(±)-5p** as a pale yellow solid (77 mg, 58%).

^{1}H NMR (400 MHz, Chloroform-*d*) δ 8.54 (dd, *J* = 2.5, 1.5 Hz, 1H), 8.48 (dd, *J* = 4.3, 2.5 Hz, 1H), 8.43 (d, *J* = 2.4 Hz, 1H), 5.13 (br s, 1H), 4.62 (d, *J* = 11.7 Hz, 1H), 3.91 – 3.76 (m, 3H), 3.56 (td, *J* = 11.6, 3.0 Hz, 1H), 3.25 (ddd, *J* = 13.4, 12.2, 3.8 Hz, 1H), 1.42 (s, 9H).

^{13}C NMR (101 MHz, Chloroform-*d*) δ 155.24, 154.44, 144.14, 143.23, 142.97, 80.87, 68.43, 66.78, 53.49, 40.90, 28.35 (3C).

HRMS (ESI-QTOF): m/z $[M+H]^+$ Calcd for $C_{13}H_{19}N_3O_3$ 266.1499; Found 266.1499. Δ = -0.03 ppm.

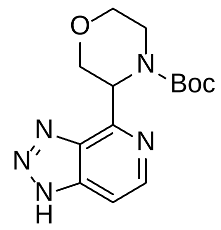

Chemical Formula: $C_{14}H_{19}N_5O_3$
Molecular Weight: 305.34
SMILES: CC(C)(C)OC(=O)N1CCOCC1c2nccc3[nH]nnc23

(±)-tert-Butyl 3-{1H-[1,2,3]triazolo[4,5-c]pyridin-4-yl}morpholine-4-carboxylate (±)-5q: Prepared according to the general procedure using 1H-[1,2,3]triazolo[4,5-c]pyridine (60 mg, 1.0 equiv, 0.5 mmol), tert-butyl morpholine-4-carboxylate (468 mg, 5.0 equiv, 2.5 mmol), {Ir[dFCF$_3$(ppy)$_2$]dtbbpy}PF$_6$ (11.2 mg, 2 mol %, 0.01 mmol), $(NH_4)_2S_2O_8$ (456 mg, 4 equiv, 2.0 mmol), TsOH.H_2O (190 mg, 2.0 equiv, 1.0 mmol). The crude product was purified by column chromatography (25g SNAP cartridge, 15–100% EtOAc/Petrol v/v) to afford **(±)-5q** (93 mg, 61%).

^{1}H NMR (400 MHz, 333K, DMSO-*d$_6$*): 8.41 (dd, J = 5.8, 0.3 Hz, 1H), 7.71 (dd, J = 5.8, 0.7 Hz, 1H), 5.56 – 5.52 (m, 1H), 4.76 (d, J = 11.7 Hz, 1H), 4.01 (dd, J = 11.8, 4.1 Hz, 1H), 3.86 – 3.81 (m, 1H), 3.79 – 3.65 (m, 2H), 3.55 (td, J = 11.0, 4.4 Hz, 1H), 1.37 – 1.18 (m, 9H).

^{13}C NMR (101 MHz, 333K, DMSO-*d$_6$*): δ 170.64, 155.70, 153.12, 143.19, 138.78, 106.10, 79.44, 69.41, 66.52, 60.12, 54.75, 28.40 (3C).

HRMS (ESI-QTOF): m/z $[M+H]^+$ Calcd for $C_{14}H_{19}N_5O_3$ 306.1561; Found 306.1562. Δ = 0.37 ppm.

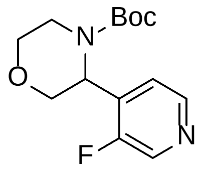

Chemical Formula: $C_{14}H_{19}FN_2O_3$
Molecular Weight: 282.32
SMILES: FC1=C(C2COCCN2C(OC(C)(C)C)=O)C=CN=C1

(±)-tert-Butyl 3-(3-fluoropyridin-4-yl)morpholine-4-carboxylate (±)-5r: Prepared according to the general procedure using 3-fluoropyridine (49.0 mg, 1.0 equiv, 0.5 mmol), tert-Butyl morpholine-4-carboxylate (468 mg, 5.0 equiv, 2.5 mmol), {Ir[dFCF$_3$(ppy)$_2$]dtbbpy}PF$_6$ (11.2 mg, 2 mol %, 0.01 mmol), $(NH_4)_2S_2O_8$ (456 mg, 4 equiv, 2.0 mmol), TsOH.H_2O (190 mg, 2.0 equiv, 1.0 mmol). The crude product was purified by column chromatography (25g SNAP cartridge, 15–100% EtOAc/Petrol v/v) to afford **(±)-5r** as a pale yellow solid (113 mg, 80%).

^{1}H NMR (400 MHz, Chloroform-*d*) δ 8.40 (d, *J* = 2.3 Hz, 1H), 8.34 (dd, *J* = 5.0, 0.9 Hz, 1H), 7.31 (dd, *J* = 6.6, 5.0 Hz, 1H), 5.24 (d, *J* = 4.1 Hz, 1H), 4.18 (d, *J* = 12.1 Hz, 1H), 3.95 – 3.74 (m, 3H), 3.57 (td, *J* = 11.8, 3.6 Hz, 1H), 3.28 (ddd, *J* = 13.5, 11.9, 4.3 Hz, 1H), 1.37 (s, 9H).

^{13}C NMR (101 MHz, Chloroform-*d*) δ 157.50 (d, *J* = 256.0 Hz), 154.61, 145.70 (d, *J* = 5.1 Hz), 138.44 (d, *J* = 25.0 Hz), 136.04 (d, *J* = 11.0 Hz), 123.04, 81.01, 68.98 (d, *J* = 3.6 Hz), 66.87, 48.82, 40.69, 28.22 (3C), 14.31.

HRMS (ESI-QTOF): m/z $[M+H]^+$ Calcd for $C_{14}H_{19}FN_2O_3$ 283.1452; Found 283.1455. Δ = 0 85 ppm.

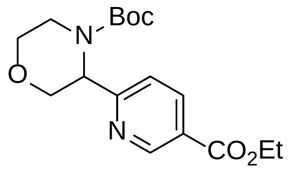

Chemical Formula: $C_{17}H_{24}N_2O_5$
Molecular Weight: 336.39
SMILES: CC(OC(N1CCOCC1C2=CC=C(C(OCC)=O)C=N2)=O)(C)C

(±)-tert-Butyl 3-[5-(ethoxycarbonyl)pyridin-2-yl]morpholine-4-carboxylate (±)-5s: Prepared according to the general procedure using ethyl pyridine-3-carboxylate (75.6 mg, 1.0 equiv, 0.5 mmol), tert-Butyl morpholine-4-carboxylate (468 mg, 5.0 equiv, 2.5 mmol), {Ir[dFCF$_3$(ppy)$_2$]dtbbpy}PF$_6$ (11.2 mg, 2 mol %, 0.01 mmol), $(NH_4)_2S_2O_8$ (456 mg, 4 equiv, 2.0 mmol), TsOH.H_2O (190 mg, 2.0 equiv, 1.0 mmol). The crude product was purified by column chromatography (25g SNAP cartridge, 10–100% EtOAc/Petrol v/v) to afford **(±)-5s** as a pale yellow solid (152 mg, 90%).

^{1}H NMR (400 MHz, Chloroform-*d*) δ 9.17 (dd, *J* = 2.2, 0.9 Hz, 1H), 8.24 (dd, *J* = 8.2, 2.2 Hz, 1H), 7.22 (dt, *J* = 8.3, 0.9 Hz, 1H), 5.08 (s, 1H), 4.71 (d, *J* = 11.6 Hz, 1H), 4.37 (q, *J* = 7.1 Hz, 2H), 3.91 – 3.75 (m, 3H), 3.54 (td, *J* = 11.7, 3.1 Hz, 1H), 3.23 (td, *J* = 13.1, 3.9 Hz, 1H), 1.43 – 1.33 (m, 12H).

^{13}C NMR (101 MHz, Chloroform-*d*) δ 165.24, 163.20, 155.40, 150.76, 137.68, 124.66, 120.40, 80.64, 68.81, 66.73, 61.34, 56.07, 40.76, 28.32 (3C).

HRMS (ESI-QTOF): m/z $[M+H]^+$ Calcd for $C_{17}H_{24}N_2O_5$ 337.1758; Found 337.1759. Δ = 0 34 ppm.

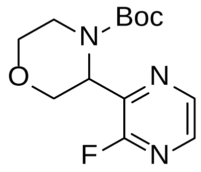

Chemical Formula: $C_{13}H_{18}FN_3O_3$
Molecular Weight: 283.30
SMILES: FC1=NC=CN=C1C2COCCN2C(OC(C)(C)C)=O

(±)-tert-butyl 3-(3-fluoropyrazin-2-yl)morpholine-4-carboxylate (±)-5t: Prepared according to the general procedure using fluoropyrazine (49.0 mg, 1.0 equiv, 0.5 mmol), tert-Butyl morpholine-4-carboxylate (468 mg, 5.0 equiv, 2.5 mmol), {Ir[dFCF$_3$(ppy)$_2$]dtbbpy}PF$_6$ (11.2 mg, 2 mol %, 0.01 mmol), $(NH_4)_2S_2O_8$ (456 mg, 4 equiv, 2.0 mmol), TsOH.H_2O (190 mg, 2.0 equiv, 1.0 mmol). The crude product was purified by reverse phase column chromatography (50g C18 Ultra cartridge, 5–50–95% MeCN/H_2O v/v with 0.1% HCO_2H modifier) to afford **(±)-5t** as an off-white solid (106 mg, 75%)

^{1}H NMR (400 MHz, Methanol-d$_4$) δ 8.49 (dd, *J* = 4.4, 2.6 Hz, 1H), 8.18 (t, *J* = 2.3 Hz, 1H), 5.21 (dd, *J* = 4.0, 1.8 Hz, 1H), 4.29 (d, *J* = 12.2 Hz, 1H), 3.92 (dt, *J* = 11.4, 3.5 Hz, 2H), 3.82 – 3.68 (m, 2H), 3.59 (td, *J* = 11.1, 4.8 Hz, 1H), 1.36 (s, 9H).

^{13}C NMR (101 MHz, Methanol-d$_4$) δ 157.85 (d, J = 253.4 Hz), 156.07, 143.99 (d, J = 24.3 Hz), 141.06 (d, J = 4.8 Hz), 140.17 (d, J = 8.4 Hz), 80.44, 67.76, 66.32, 51.74, 41.67, 27.06 (3C).

HRMS (ESI-QTOF): A mass could not be observed for this compound with the applied ionisation technique. For full characterization by NMR see p SI-75.

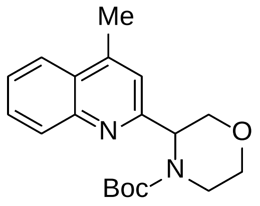

Chemical Formula: $C_{19}H_{24}N_2O_3$
Molecular Weight: 328.41
SMILES: Cc2cc(C1COCCN1C(=O)OC(C)(C)C)nc3ccccc23

(±)-tert-Butyl 3-(4-methylquinolin-2-yl)morpholine-4-carboxylate (±)-5u: Prepared according to the general procedure using 4-methylquinoline (71.6 mg, 0.5 mmol) and tert-Butyl morpholine-4-carboxylate (468 mg, 2.5 mmol). The crude product was purified by column chromatography (25g SNAP cartridge, 5–40% EtOAc/Petrol v/v) to afford **(±)-5u** as an off-white solid (123.5 mg, 75%).

^{1}H NMR (400 MHz, Chloroform-*d*): δ 8.04 (ddd, *J* = 8.6, 1.4, 0.6 Hz, 1H), 7.95 (ddd, *J* = 8.4, 1.5, 0.6 Hz, 1H), 7.66 (ddd, *J* = 8.4, 6.8, 1.5 Hz, 1H), 7.51 (ddd, *J* = 8.2, 6.9, 1.3 Hz, 1H), 7.13 (s, 1H), 5.18 (br s, 1H), 4.91 (d, *J* = 11.5 Hz, 1H), 3.94 – 3.80 (m, 3H), 3.61 (ddd, *J* = 12.1, 11.2, 2.9 Hz, 1H), 3.40 (ddt, *J* = 13.5, 9.0, 3.8 Hz, 1H), 2.69 (d, *J* = 1.0 Hz, 3H), 1.46 (s, 9H).

^{13}C NMR (101 MHz, Chloroform-*d*): δ 158.49, 155.96, 147.87, 144.95, 130.08, 129.18, 127.11, 126.08, 123.63, 119.19, 80.99, 68.93, 66.83, 56.50, 41.19, 28.42, 19.09 (3C).

HRMS (ESI-QTOF): m/z $[M+H]^+$ Calcd for $C_{19}H_{24}N_2O_3$ 329.1860; Found 329.1860. Δ = 0 02 ppm.

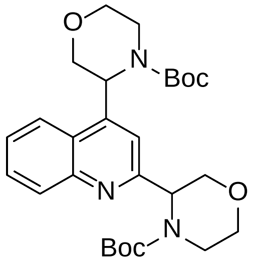

Chemical Formula: $C_{27}H_{37}N_3O_6$
Molecular Weight: 499.61
SMILES:
C(C)(C)OC(=O)N1CCOCC1c2cc(C3COCCN3C(=O)OC(C)(C)C)c4ccccc4n2

tert-Butyl 3-(4-{4-[(tert-butoxy)carbonyl]morpholin-3-yl}quinolin-2-yl)morpholine-4-carboxylate (±)-5v: Prepared according to the general procedure using quinoline (65 mg, 1.0 equiv, 0.5 mmol), tert-Butyl morpholine-4-carboxylate (468 mg, 5.0 equiv, 2.5 mmol), {Ir[dFCF$_3$(ppy)$_2$]dtbbpy}PF$_6$ (11.2 mg, 2 mol %, 0.01 mmol), $(NH_4)_2S_2O_8$ (456 mg, 4 equiv, 2.0 mmol), TsOH.H_2O (190 mg, 2.0 equiv, 1.0 mmol). The crude product was purified (25g SNAP cartridge, 0–40% EtOAc/Petrol v/v) to afford **(±)-5v** as a yellow solid (189 mg, 76%).

^{1}H NMR (400 MHz, Chloroform-*d*): (mixture of rotamers) δ 8.18 – 8.06 (m, 2H), 7.73 – 7.61 (m, 2H), 7.51 (ddd, *J* = 8.2, 6.8, 1.4 Hz, 1H), 5.72 (t, *J* = 5.8 Hz, 1H), 5.24 (s, 1H), 4.96 – 4.82 (m, 1H), 4.28 (d, *J* = 12.0 Hz, 1H), 4.02 (dd, J = 12.0, 4.2 Hz, 1H), 3.98 – 3.76 (m, 5H), 3.62 (dddd, J = 16.8, 14.0, 11.5, 3.0 Hz, 2H), 3.49 – 3.16 (m, 2H), 2.00 – 0.78 (m, 18H).

^{13}C NMR (101 MHz, Chloroform-*d*): (mixture of rotamers) δ 158.39, 155.79, 155.74, 154.69, 154.60, 148.55, 148.47, 146.11, 130.50, 129.19, 126.47, 125.50, 125.37, 123.49, 123.35, 118.16, 117.69, 80.95, 80.93, 80.43, 80.39, 69.19, 69.17, 69.08, 67.16, 67.07, 66.83, 66.78, 56.51, 50.62, 41.35, 41.25, 28.46 (rotamer singlet, 3C), 28.42 (rotamer singlet, 3C), 28.38 (rotamer singlet, 3C), 28.36 (rotamer singlet, 3C).

HRMS (ESI-QTOF): m/z $[M+H]^+$ Calcd for $C_{27}H_{37}N_3O_6$ 500.2755; Found 500.2754. Δ = -0 23 ppm.

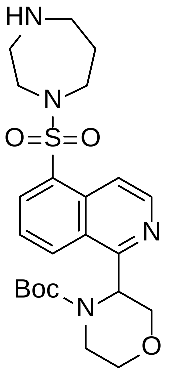

Chemical Formula: $C_{23}H_{32}N_4O_5S$
Molecular Weight: 476.59
SMILES: CC(C)(C)OC(=O)N1CCOCC1c2nccc3c(cccc23)S(=O)(=O)N4CCCNCC4

(±)-tert-Butyl 3-[5-(1,4-diazepane-1-sulfonyl)isoquinolin-1-yl]morpholine-4-carboxylate (±)-5y: Prepared according to the general procedure using fausdil monohydrochloride (163.9 mg, 1.0 equiv, 0.5 mmol), tert-Butyl morpholine-4-carboxylate (468 mg, 5.0 equiv, 2.5 mmol), {Ir[dFCF$_3$(ppy)$_2$]dtbbpy}PF$_6$ (11.2 mg, 2 mol %, 0.01 mmol), $(NH_4)_2S_2O_8$ (456 mg, 4 equiv, 2.0 mmol), TsOH.H_2O (190 mg, 2.0 equiv, 1.0 mmol). The crude product was purified (25g SNAP cartridge, 0–20% MeOH/CH_2Cl_2 v/v) to afford **(±)-5y** as a yellow oil (189 mg, 76%).

^{1}H NMR (400 MHz, Chloroform-d): δ 8.63 (d, *J* = 6.1 Hz, 1H), 8.39 – 8.29 (m, 3H), 7.64 (dd, *J* = 8.6, 7.4 Hz, 1H), 5.84 (s, 1H), 4.33 (d, *J* = 11.7 Hz, 1H), 4.09 – 3.94 (m, 3H), 3.84 – 3.75 (m, 1H), 3.65 (td, *J* = 11.6, 11.0, 3.1 Hz, 1H), 3.55 – 3.44 (m, 4H), 3.13 (br s, 1H), 3.09 – 2.98 (m, 4H), 1.97 – 1.85 (m, 2H), 1.34 – 1.29 (br s, 9H).

^{13}C NMR (101 MHz, Chloroform-d): δ 160.05, 155.94, 143.42, 135.56, 132.50, 132.46, 129.54, 126.62, 125.72, 116.78, 80.48, 69.42, 67.12, 53.22, 50.18 (2C), 47.36 (2C), 42.40, 30.32, 28.35 (3C).

HRMS (ESI-QTOF): m/z $[M+H]^+$ Calcd for $C_{23}H_{32}N_4O_5S$ 477.2166; Found 477.2164. Δ = -0 47 ppm.

UHPLC-MS spectra of purified compounds - *method detailed on pages SI-3 & 4*

(±)-tert-Butyl 3-(5-bromoisoquinolin-1-yl)morpholine-4-carboxylate(±)-5a

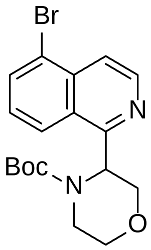

LC-MS $[M+H]^+$ = 393

Purity = 98.8%

Group	Rt	Area	A%Total	%Max Peak
2	0.652	0.88	0.1	0.1
3	0.796	2.04	0.3	0.3
4	0.955	1.64	0.2	0.2
5	1.186	660.28	98.8	100.0
6	1.212	1.30	0.2	0.2
7	1.230	2.10	0.3	0.3

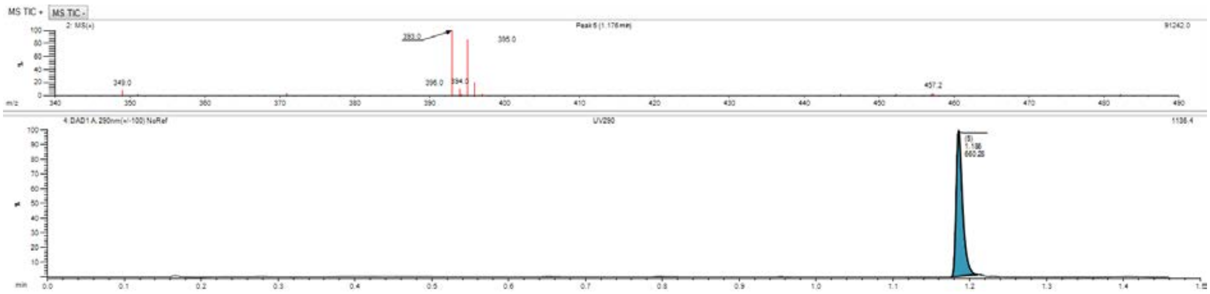

(±)-tert-Butyl 2-(5-bromoisoquinolin-1-yl)azetidine-1-carboxylate (±)-5b

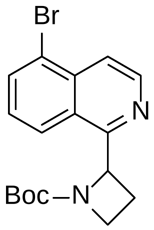

LC-MS $[M+H]^+$ = 363

Purity = 96.7%

MS TIC + | MS TIC - | UV290 | ELSD

Group	Rt	Area	A%Total	%Max Peak
4	0.751	1.55	0.3	0.4
6	0.849	0.59	0.1	0.1
7	0.882	1.22	0.3	0.3
10	1.078	429.36	96.7	100.0
12	1.162	0.74	0.2	0.2
13	1.194	0.32	0.1	0.1
15	1.258	10.34	2.3	2.4

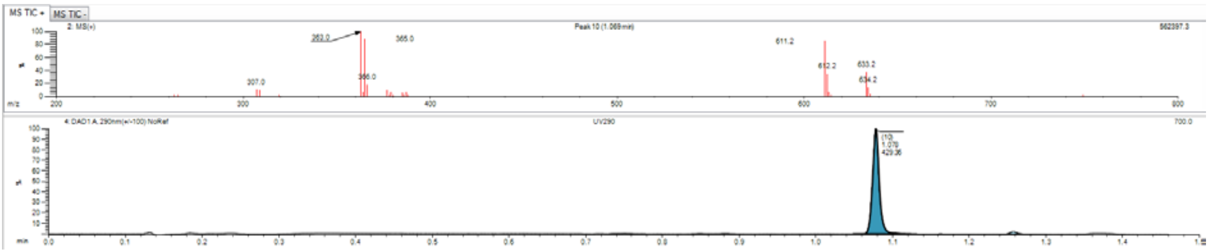

(±)-*trans*-1-tert-butyl 3-methyl 2-(5-bromoisoquinolin-1-yl)azetidine-1,3-dicarboxylate (±)-5c

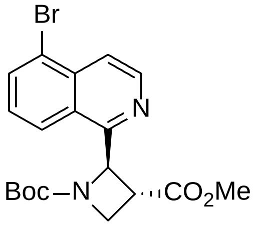

LC-MS $[M+H]^+$ = 421

Purity = >99.9%

MS TIC + | MS TIC - | UV290 | ELSD

Group	Rt	Area	A%Total	%Max Peak
4	1.096	41.13	100.0	100.0

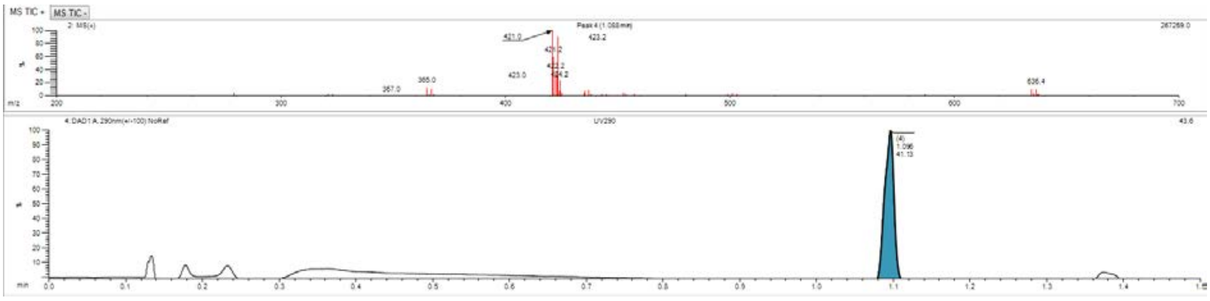

(±)-*trans*-tert-Butyl 2-(5-bromoisoquinolin-1-yl)-3-methylazetidine-1-carboxylate (±)-5d

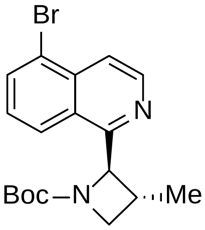

LC-MS $[M+H]^+$ = 377

Purity = 99.0%

Group	Rt	Area	A%Total	%Max Peak
1	1.145	67.39	99.0	100.0
2	1.183	0.66	1.0	1.0

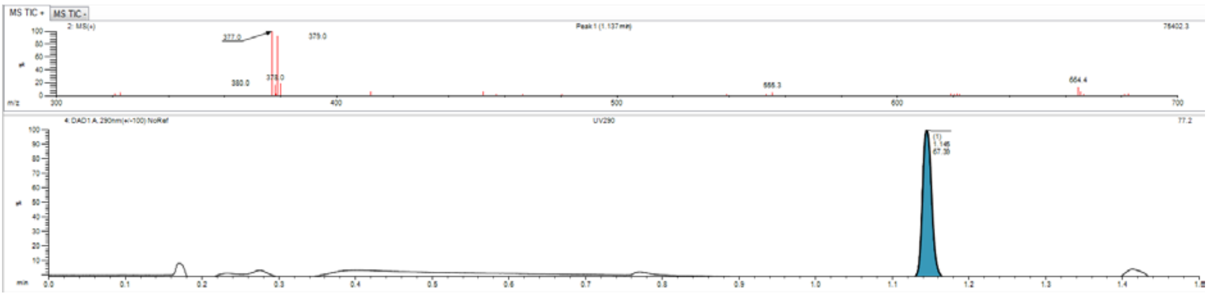

(±)-tert-Butyl 1-(5-bromoisoquinolin-1-yl)-6-oxo-2-azaspiro[3.3]heptane-2-carboxylate (±)-5e

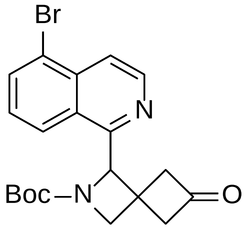

LC-MS $[M+H]^+$ = 417

Purity = 97.9%

Group	Rt	Area	A%Total	%Max Peak
7	0.982	18.19	0.5	0.5
8	1.044	3516.36	97.9	100.0
11	1.157	56.23	1.6	1.6

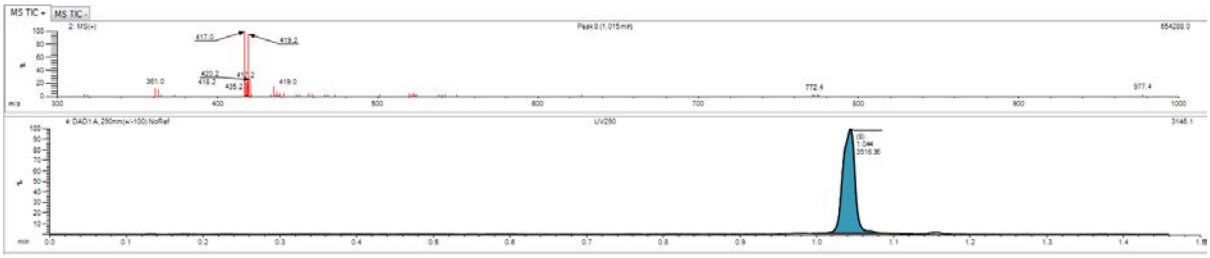

(±)-7-benzyl 1-tert-butyl 2-(5-bromoisoquinolin-1-yl)-1,7-diazaspiro[3.5]nonane-1,7-dicarboxylate (±)-5f

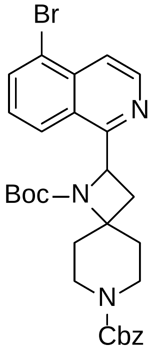

LC-MS $[M+H]^+$ = 566

Purity = 96.0%

Group	Rt	Area	A%Total	%Max Peak
3	0.782	2.12	2.0	2.1
4	1.093	0.82	0.8	0.8
5	1.158	0.69	0.6	0.7
6	1.185	0.69	0.6	0.7
7	1.226	102.73	96.0	100.0

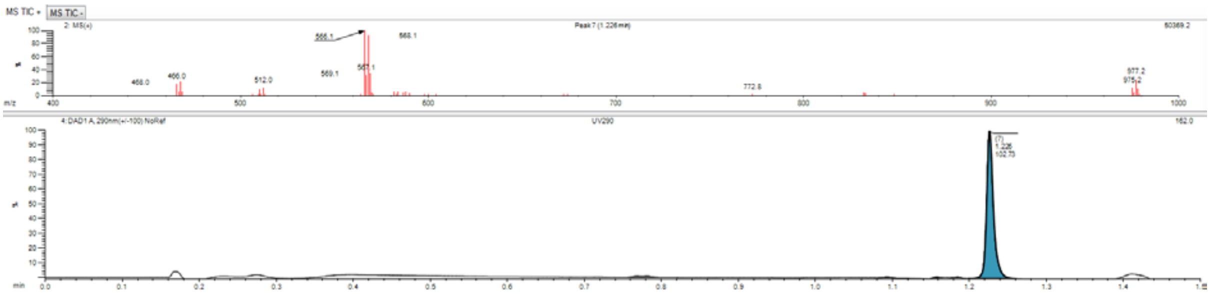

(±)-tert-Butyl 2-(5-bromoisoquinolin-1-yl)pyrrolidine-1-carboxylate (±)-5g

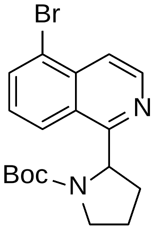

LC-MS $[M+H]^+$ = 377

Purity = 98.1%

Group	Rt	Area	A%Total	%Max Peak
2	0.645	7.19	0.5	0.5
3	0.771	1.36	0.1	0.1
6	1.136	1511.41	98.1	100.0
7	1.221	1.77	0.1	0.1
8	1.289	19.04	1.2	1.3

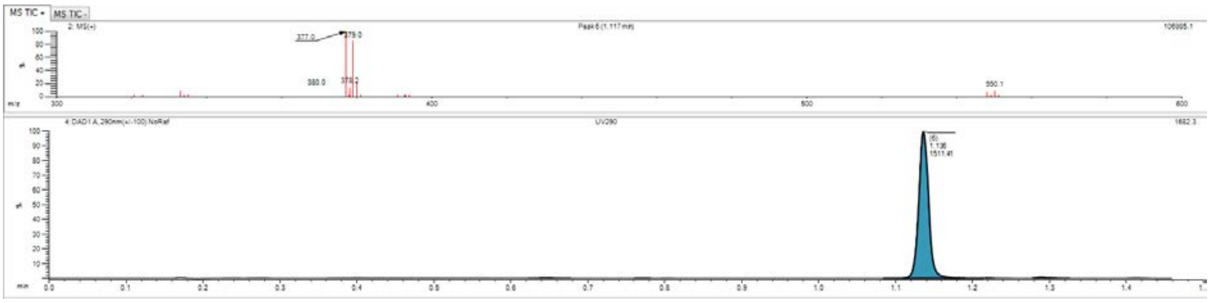

(±)-*trans*-tert-Butyl 2-(5-bromoisoquinolin-1-yl)-3-azabicyclo[3.1.0]hexane-3-carboxylate (±)-5h

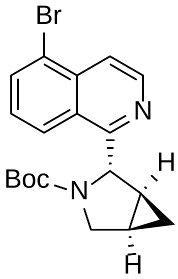

LC-MS $[M+H]^+$ = 389

Purity = >99.9%

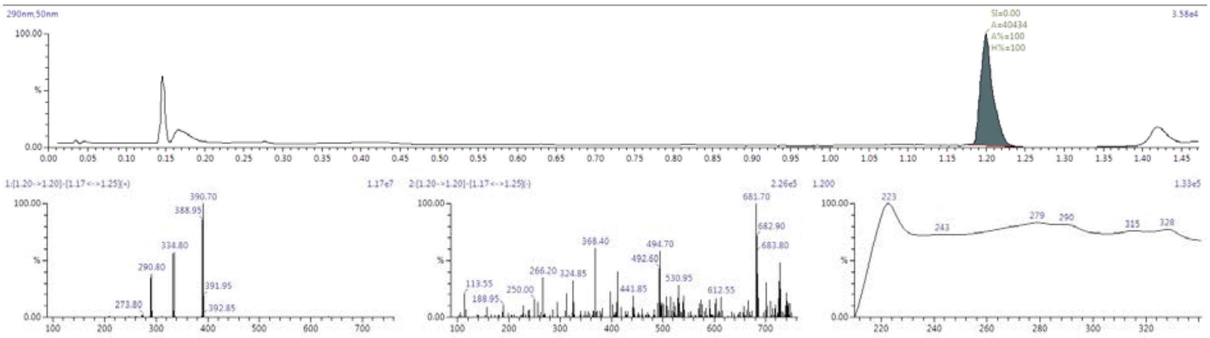

(±)-tert-Butyl 3-(5-bromoisoquinolin-1-yl)thiomorpholine-4-carboxylate (±)-5i

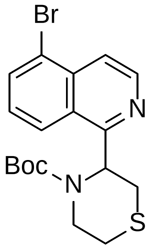

LC-MS $[M+H]^+$ = 409

Purity = 93.0%

Group	Rt	Area	A%Total	%Max Peak
1	0.784	3.17	0.1	0.2
2	0.826	1.66	0.1	0.1
3	0.859	1.32	0.1	0.1
4	0.889	9.73	0.4	0.5
5	0.930	1.18	0.1	0.1
6	1.076	126.14	5.6	6.0
7	1.103	0.89	0.0	0.0
8	1.150	0.68	0.0	0.0
9	1.185	0.63	0.0	0.0
10	1.214	2093.44	93.0	100.0
11	1.258	7.23	0.3	0.3
12	1.280	2.32	0.1	0.1
13	1.307	1.53	0.1	0.1

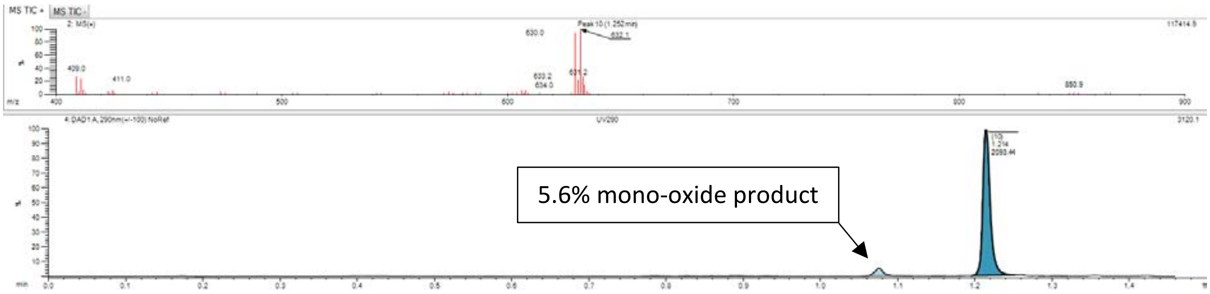

(±)-tert-Butyl 4-acetyl-2-(5-bromoisoquinolin-1-yl)piperazine-1-carboxylate (±)-5j-major & (±)-tert-Butyl 4-acetyl-3-(5-bromoisoquinolin-1-yl)piperazine-1-carboxylate (±)-5j-minor.

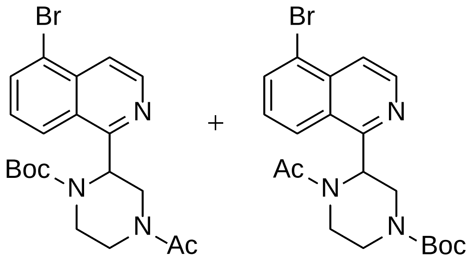

(±)5j-major **(±)5j**-minor

LC-MS $[M+H]^+$ = 434

Purity = 97.5%

(Overlapping regio-isomers on LCMS chromatogram)

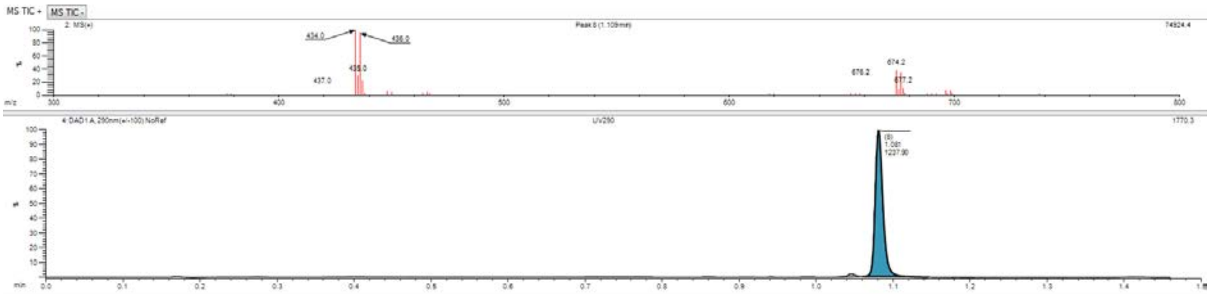

(±)-*trans*-tert-Butyl 6-(5-bromoisoquinolin-1-yl)-2-oxa-5-azabicyclo[2.2.1]heptane-5-carboxylate (±)-5k

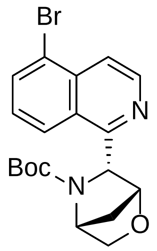

LC-MS $[M+H]^+$ = 405

Purity = 95.3%

Group	Rt	Area	A%Total	%Max Peak
5	0.564	0.85	1.2	1.3
12	1.064	1.85	2.6	2.8
13	1.080	66.63	95.3	100.0
14	1.168	0.57	0.8	0.9

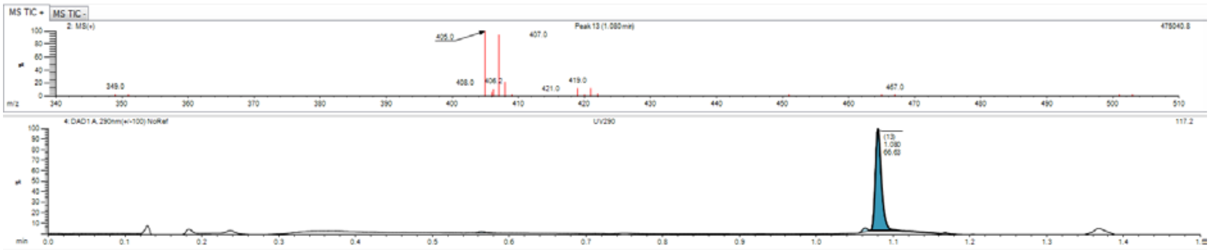

(±)-*trans*-tert-Butyl 3-(5-bromoisoquinolin-1-yl)-5-oxo-2-azabicyclo[2.2.1]heptane-2-carboxylate (±)-5l

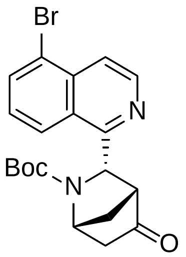

LC-MS $[M+H]^+$ = 417

Purity = 96.5%

Group	Rt	Area	A%Total	%Max Peak
1	0.878	1.28	1.2	1.3
2	1.018	1.32	1.3	1.3
3	1.106	98.84	96.5	100.0
4	1.185	1.00	1.0	1.0

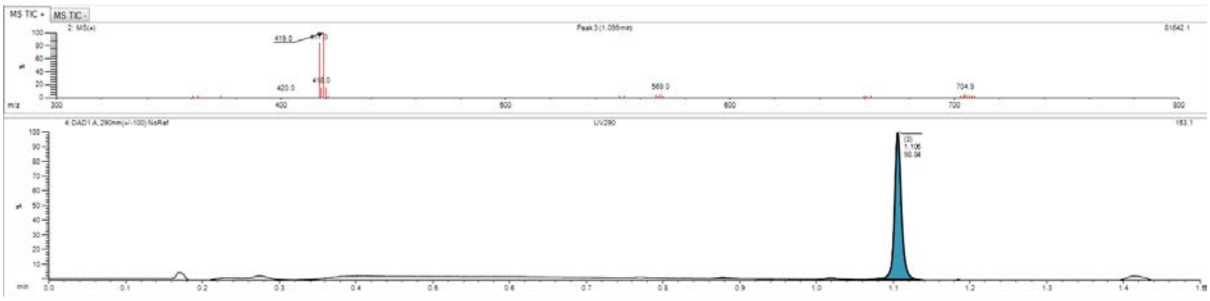

(±)-tert-Butyl 3-(pyridin-4-yl)morpholine-4-carboxylate (±)-5m

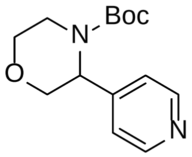

LC-MS $[M+H]^+$ = 265

Purity = 95.3%

Group	Rt	Area	A%Total	%Max Peak
1	0.608	771.40	95.3	100.0
2	0.709	11.68	1.4	1.5
3	0.768	1.27	0.2	0.2
4	0.901	3.80	0.5	0.5
5	0.953	14.17	1.8	1.8
6	1.015	1.96	0.2	0.3
7	1.065	1.78	0.2	0.2
8	1.094	1.41	0.2	0.2
9	1.185	2.06	0.3	0.3

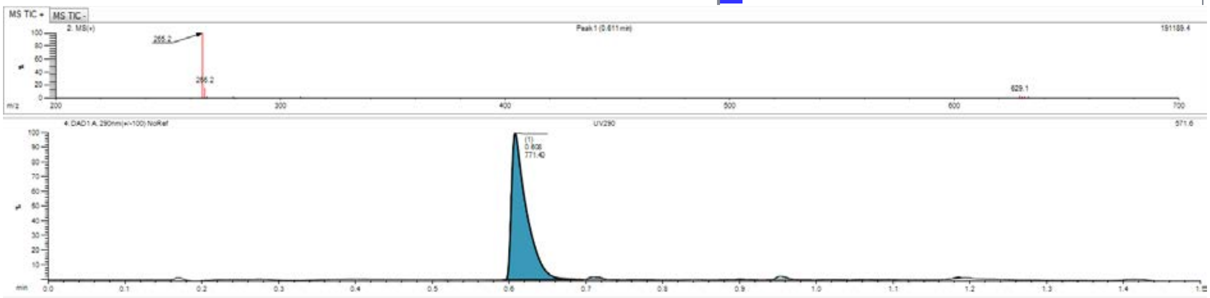

(±)-tert-Butyl 3-(pyridazin-4-yl)morpholine-4-carboxylate (±)-5n

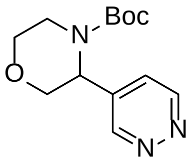

LC-MS $[M+H]^+$ = 266

Purity = 96.4%

	Group	Rt	Area	A%Total	%Max Peak
	2	0.539	1.26	0.2	0.2
	3	0.664	0.86	0.1	0.1
	4	0.677	0.34	0.1	0.1
	5	0.732	9.58	1.5	1.6
	6	0.768	608.64	96.4	100.0
	7	0.833	8.89	1.4	1.5
	8	1.001	1.11	0.2	0.2
	10	1.184	0.60	0.1	0.1

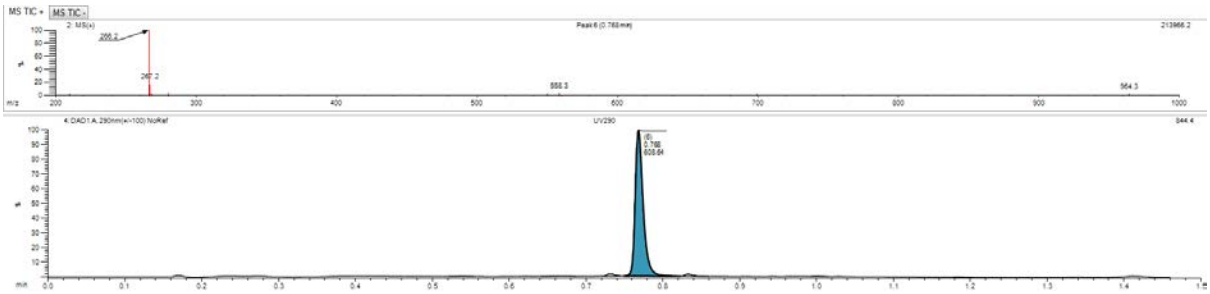

(±)-tert-Butyl 3-(pyrimidin-4-yl)morpholine-4-carboxylate (±)-5o

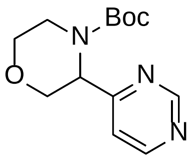

LC-MS $[M+H]^+$ = 266

Purity >99.9%

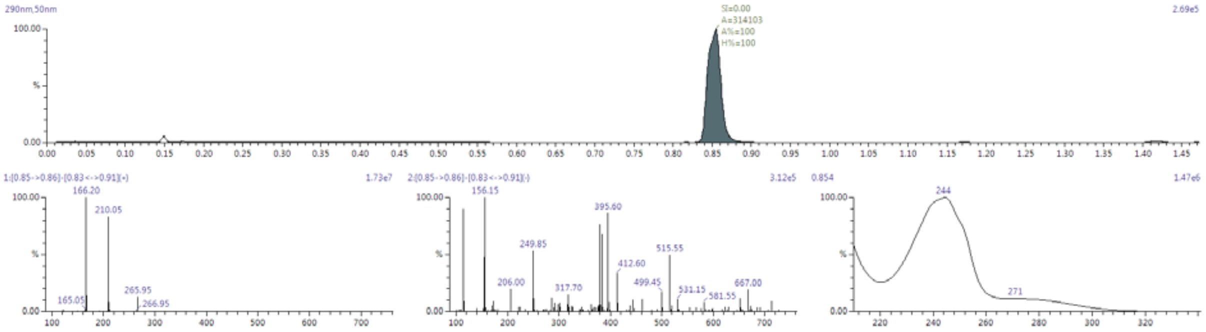

(±)-tert-Butyl 3-(pyrazin-2-yl)morpholine-4-carboxylate (±)-5p

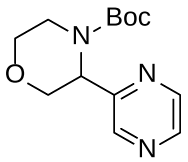

LC-MS $[M+H]^+$ = 266

Purity = 98.8%

Group	Rt	Area	A%Total	%Max Peak
1	0.855	75.91	98.8	100.0
2	1.185	0.96	1.2	1.3

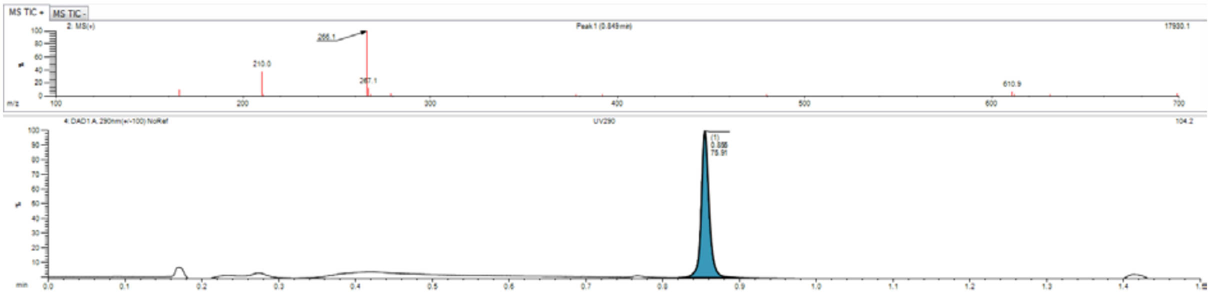

(±)-tert-Butyl 3-{1H-[1,2,3]triazolo[4,5-c]pyridin-4-yl}morpholine-4-carboxylate (±)-5q

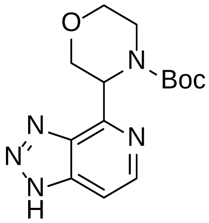

LC-MS $[M+H]^+$ = 306

Purity = 98.2%

Group	Rt	Area	A%Total	%Max Peak
1	0.810	56.88	98.2	100.0
3	1.185	1.03	1.8	1.8

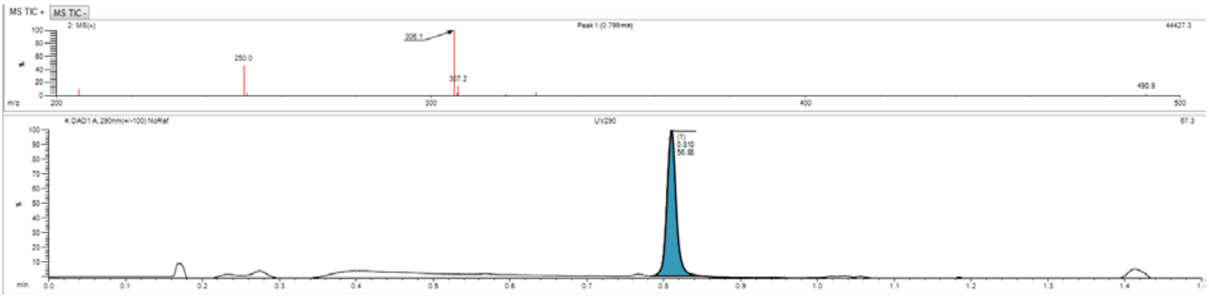

(±)-tert-Butyl 3-(3-fluoropyridin-4-yl)morpholine-4-carboxylate (±)-5r

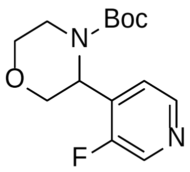

LC-MS $[M+H]^+$ = 283

Purity = >99.9%

Group	Rt	Area	A%Total	%Max Peak
1	0.859	61.09	100.0	100.0

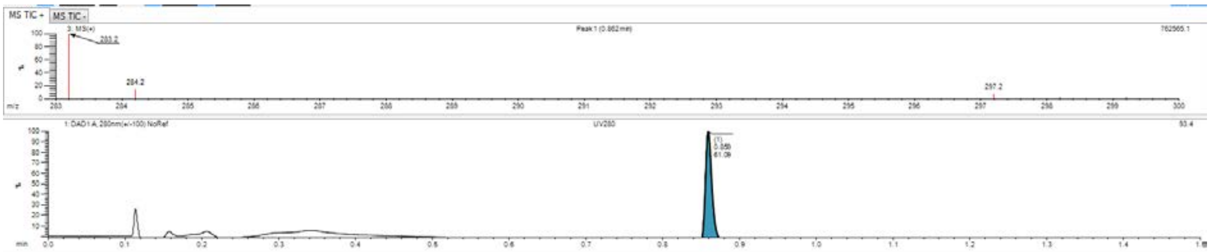

(±)-tert-Butyl 3-[5-(ethoxycarbonyl)pyridin-2-yl]morpholine-4-carboxylate (±)-5s

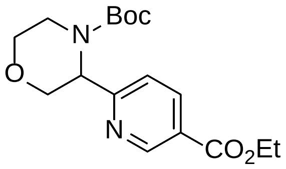

LC-MS $[M+H]^+$ = 336

Purity = 97.3%

Group	Rt	Area	A%Total	%Max Peak
1	0.616	0.59	0.1	0.1
2	0.654	2.99	0.4	0.5
3	0.767	1.21	0.2	0.2
4	0.900	1.23	0.2	0.2
5	0.919	1.10	0.2	0.2
6	0.942	0.65	0.1	0.1
7	0.989	0.56	0.1	0.1
8	1.060	659.37	97.3	100.0
9	1.086	0.33	0.0	0.1
10	1.101	5.87	0.9	0.9
11	1.158	0.27	0.0	0.0
12	1.175	3.53	0.5	0.5

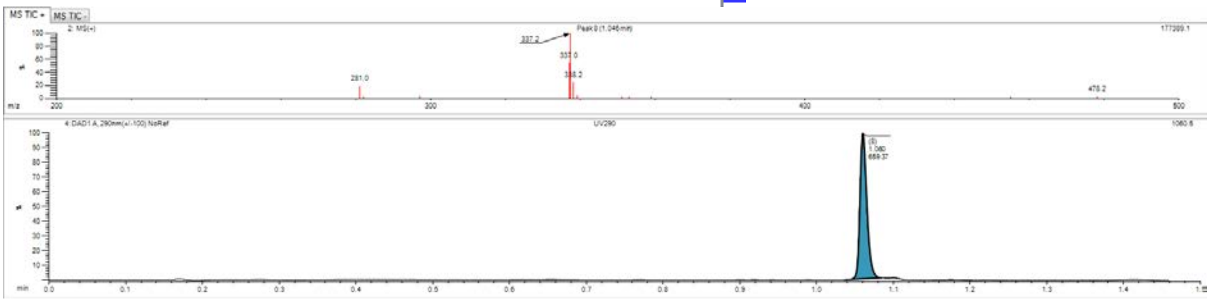

(±)-tert-butyl 3-(3-fluoropyrazin-2-yl)morpholine-4-carboxylate (±)-5t

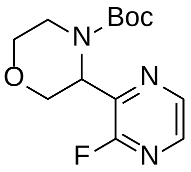

LC-MS $[M+H]^+ = 284$

Purity = >99.9%

Group	Rt	Area	A%Total	%Max Peak
1	0.904	55.65	100.0	100.0

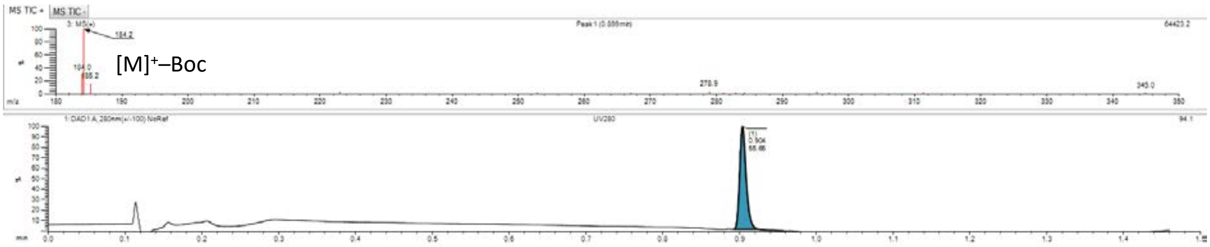

tert-Butyl 3-(4-{4-[(tert-butoxy)carbonyl]morpholin-3-yl}quinolin-2-yl)morpholine-4-carboxylate (±)-5u

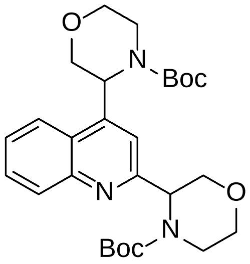

LC-MS $[M+H]^+ = 500$

Purity = 92.3%

Group	Rt	Area	A%Total	%Max Peak
1	0.636	0.97	0.1	0.1
2	0.778	60.21	5.4	5.9
3	0.838	3.22	0.3	0.3
4	0.894	1.23	0.1	0.1
5	1.013	1.40	0.1	0.1
6	1.066	2.10	0.2	0.2
7	1.095	14.60	1.3	1.4
8	1.124	0.86	0.1	0.1
9	1.156	1020.47	92.3	100.0

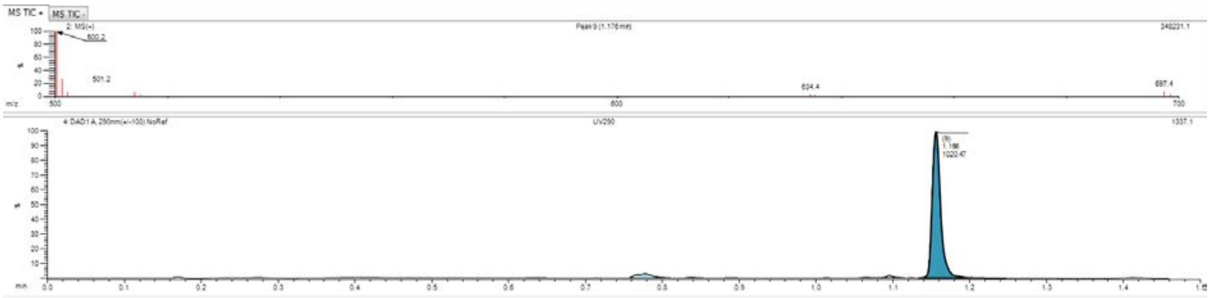

(±)-tert-Butyl 3-(4-methylquinolin-2-yl)morpholine-4-carboxylate (±)-5v

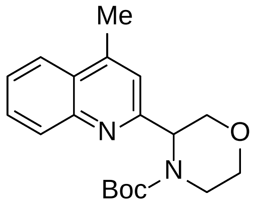

LC-MS $[M+H]^+$ = 329

Purity = 94.0 %

Group	Rt	Area	A%Total	%Max Peak
1	0.475	9.21	0.4	0.4
2	0.498	40.40	1.7	1.8
3	0.535	77.93	3.2	3.4
4	0.669	10.89	0.4	0.5
5	0.740	2.59	0.1	0.1
6	0.800	0.53	0.0	0.0
8	1.080	2290.08	94.0	100.0
10	1.149	1.60	0.1	0.1
11	1.184	1.03	0.0	0.0
12	1.203	1.88	0.1	0.1
13	1.227	0.68	0.0	0.0

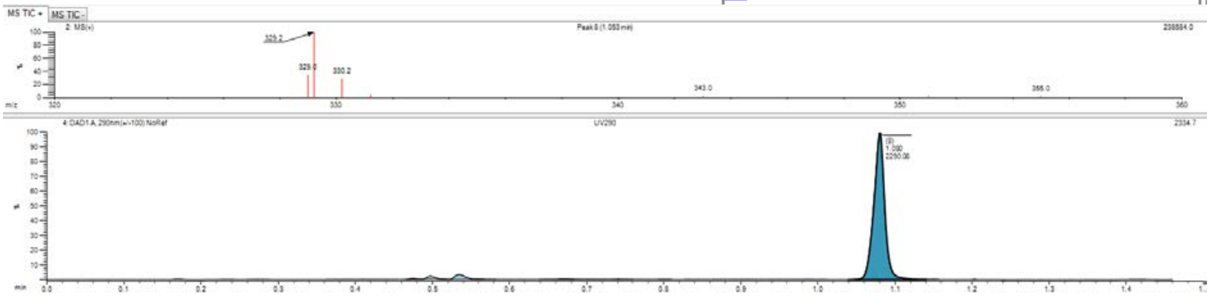

(±)-tert-Butyl 3-[5-(1,4-diazepane-1-sulfonyl)isoquinolin-1-yl]morpholine-4-carboxylate (±)-5w

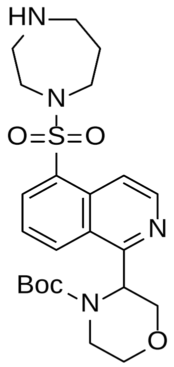

LC-MS $[M+H]^+$ = 477

Purity = 97.1%

Group	Rt	Area	A%Total	%Max Peak
1	0.794	33.22	97.1	100.0
2	1.183	0.98	2.9	3.0

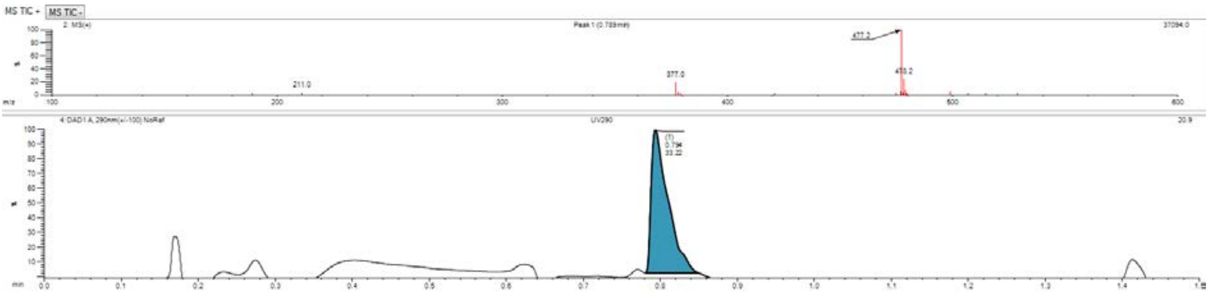

Exemplar spectra – Variable temperature experiments

(±)-tert-Butyl 2-(5-bromoisoquinolin-1-yl)pyrrolidine-1-carboxylate (±)-5g

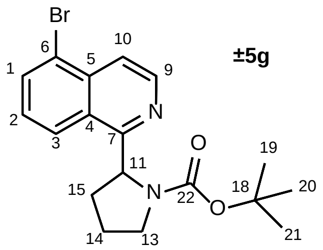

±5g exists as a mixture of rotamers in solution, in the ^{1}H NMR spectra many of these isomeric signals coalesce or sharpen at 90°C (363K) except the tert-butyl protons of the Boc group (19–21) (Figure SI-12). This exchange is also observed in the ^{13}C spectra, however elevated temperatures cause broadening and loss of resolution of several carbon signals (Figure SI-13).

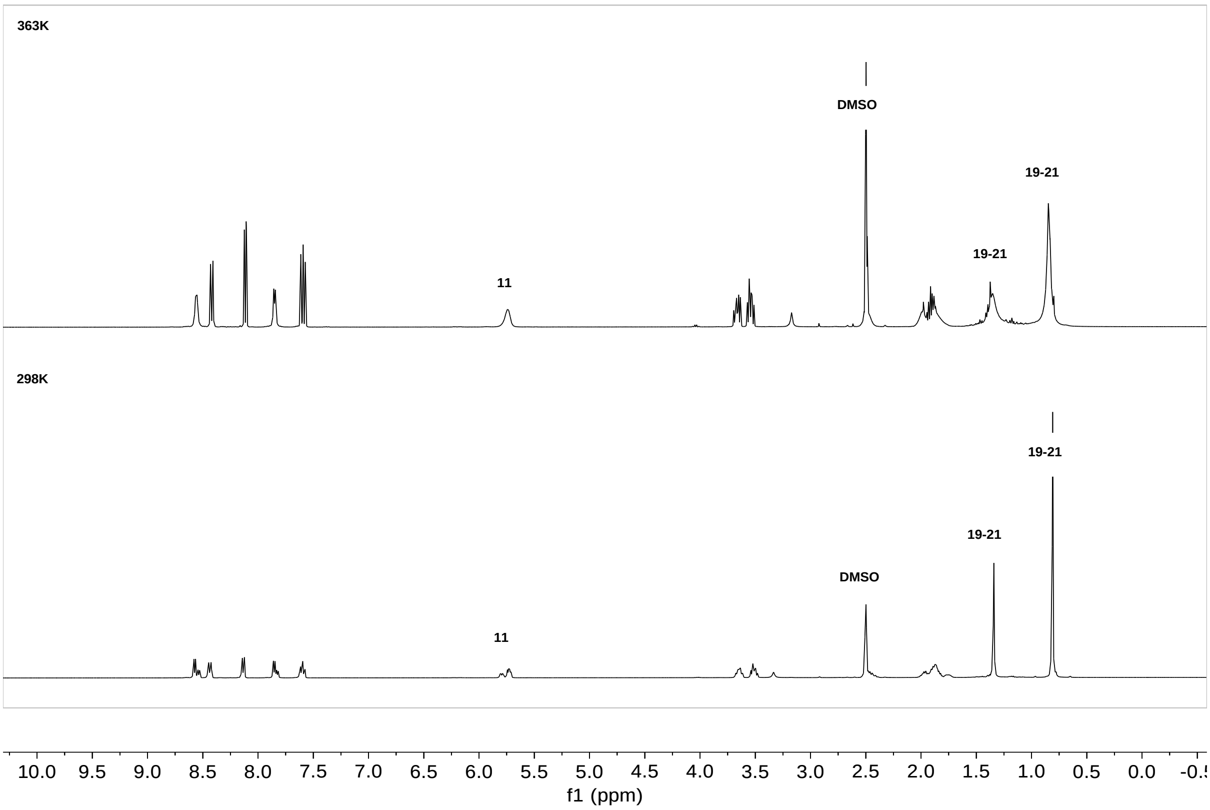

Figure SI-12: Variable temperature ^{1}H spectra of **(±)-5g**. At 363K, the two rotameric forms of **(±)-5h** begin to interconvert resulting in sharpening of signals in the ^{1}H NMR spectra, however protons *19-21* on the Boc group are still significantly split as two singlets separated by >0.5ppm.

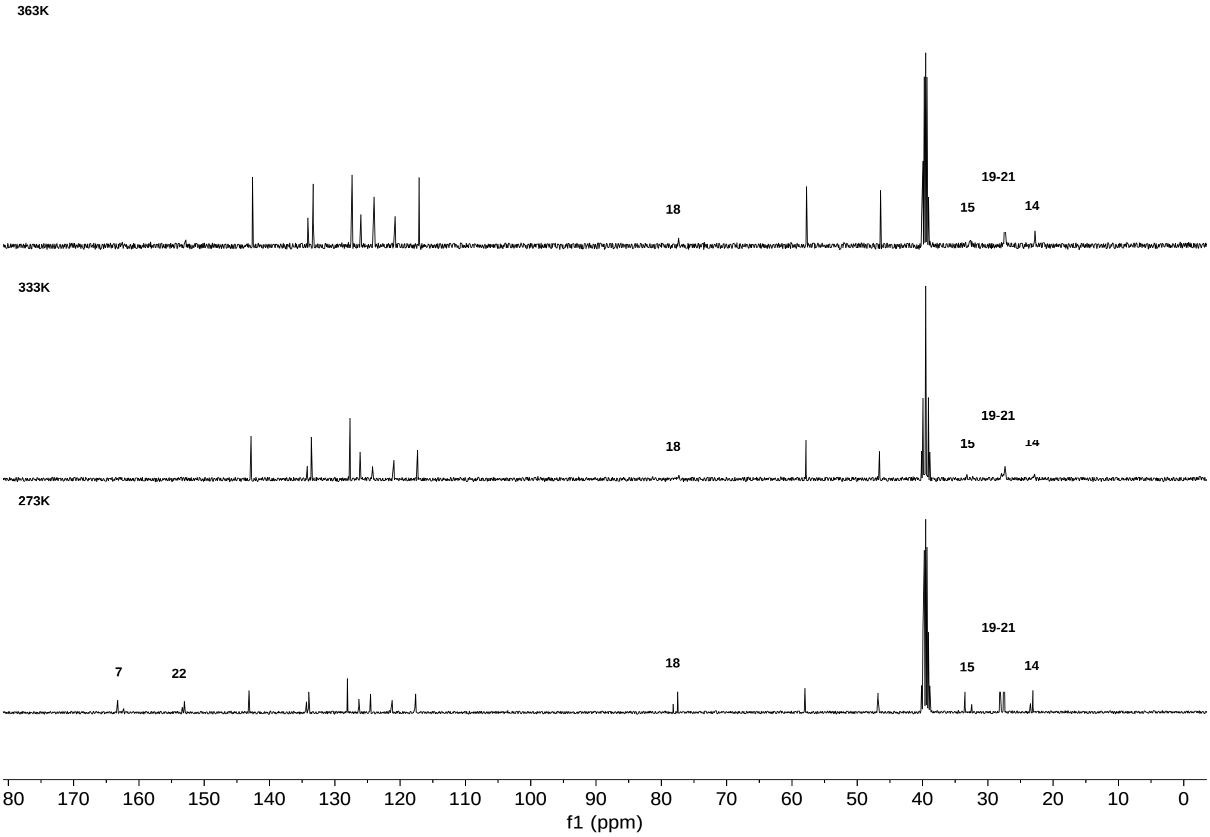

Figure SI-13: Variable temperature ^{13}C spectra of **(±)-5g**. Elevated temperatures did not significantly improve the rotameric signals in the ^{13}C spectra (like in the ^{1}H spectra) and actually resulted in the loss of several signals relating to quaternary carbons.

Exemplar spectra – Full spectral elucidation

(±)-tert-Butyl 3-(5-bromoisoquinolin-1-yl)morpholine-4-carboxylate(±)-5a

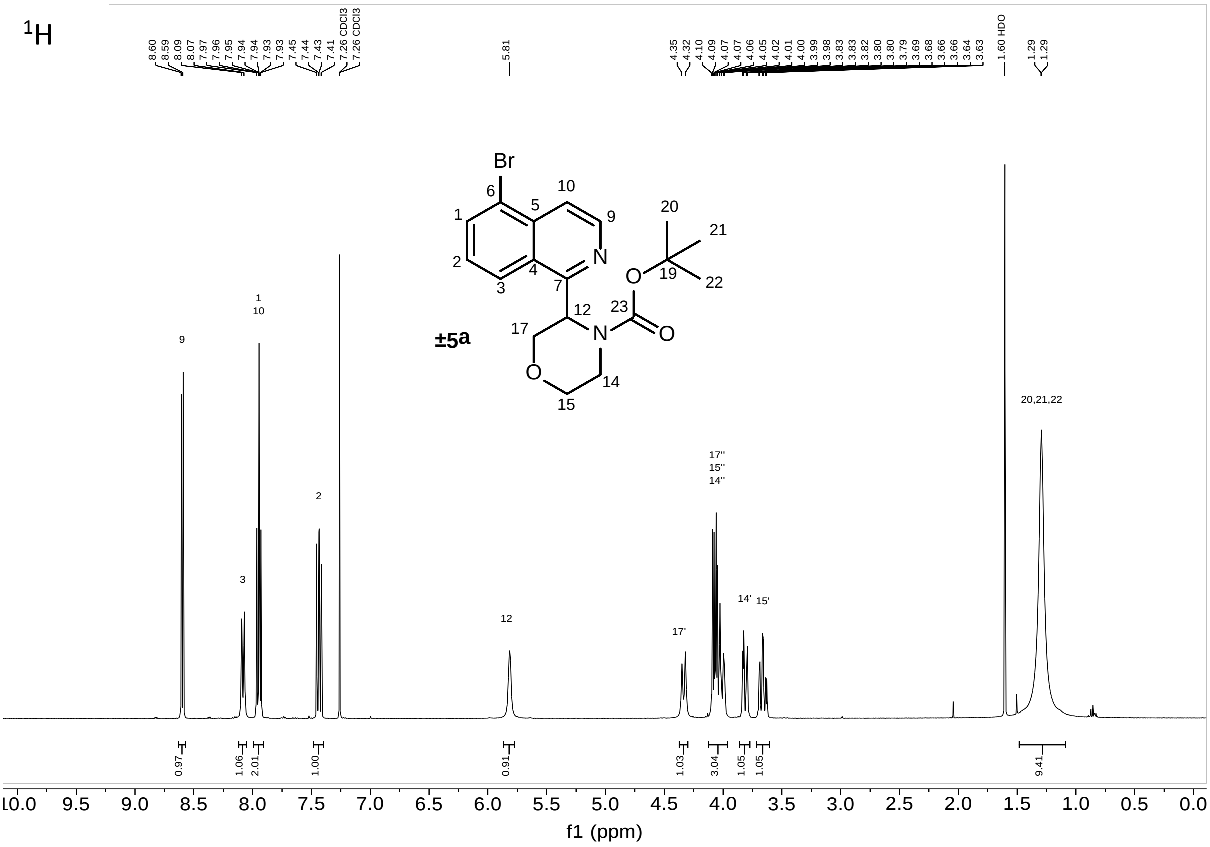

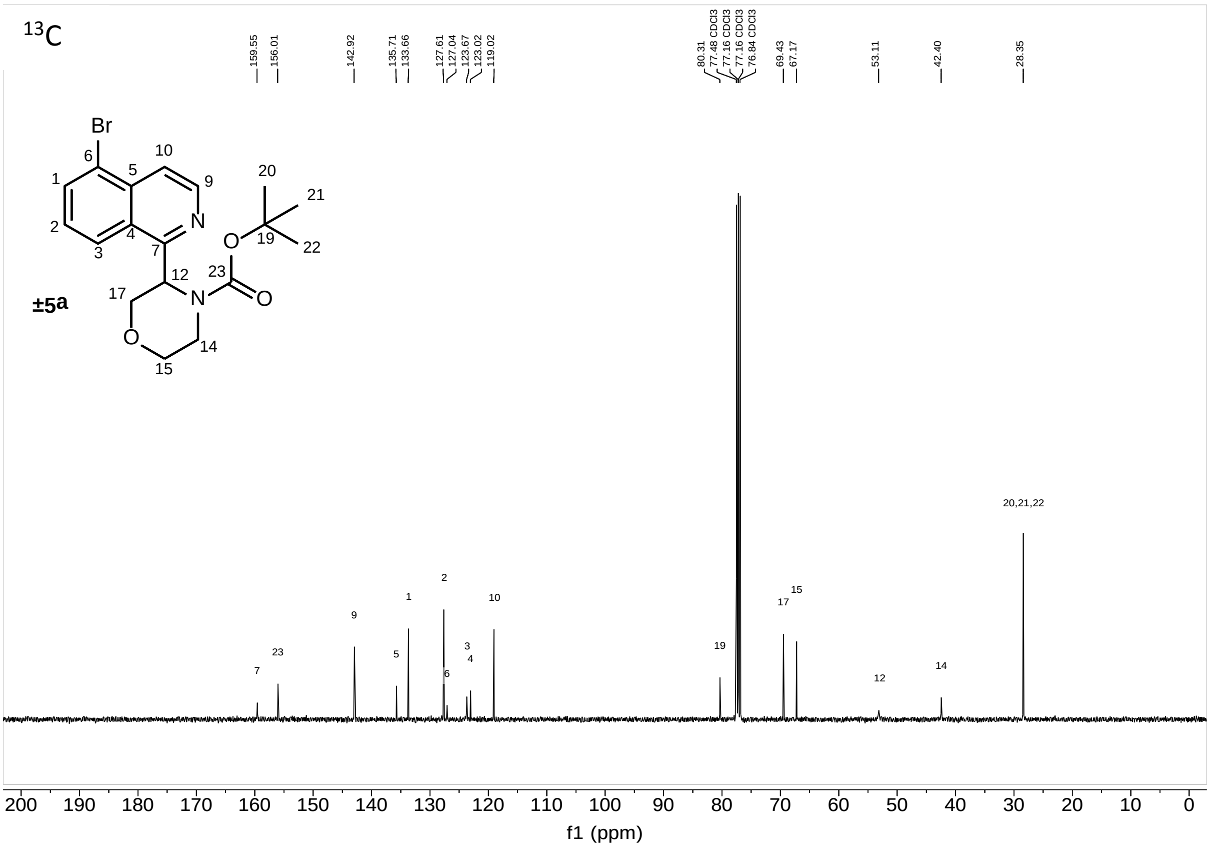

(±)-*trans*-1-tert-Butyl 3-methyl 2-(5-bromoisoquinolin-1-yl)azetidine-1,3-dicarboxylate (±)-5c

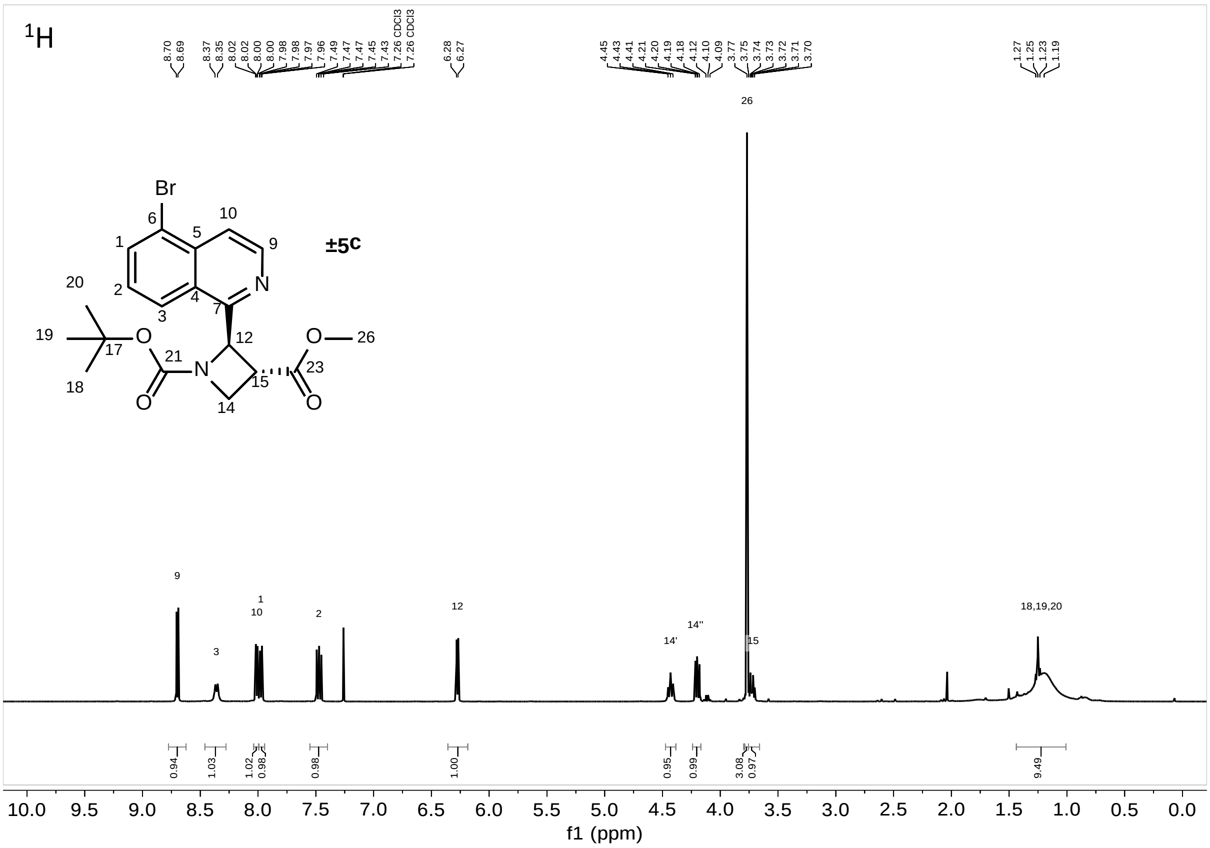

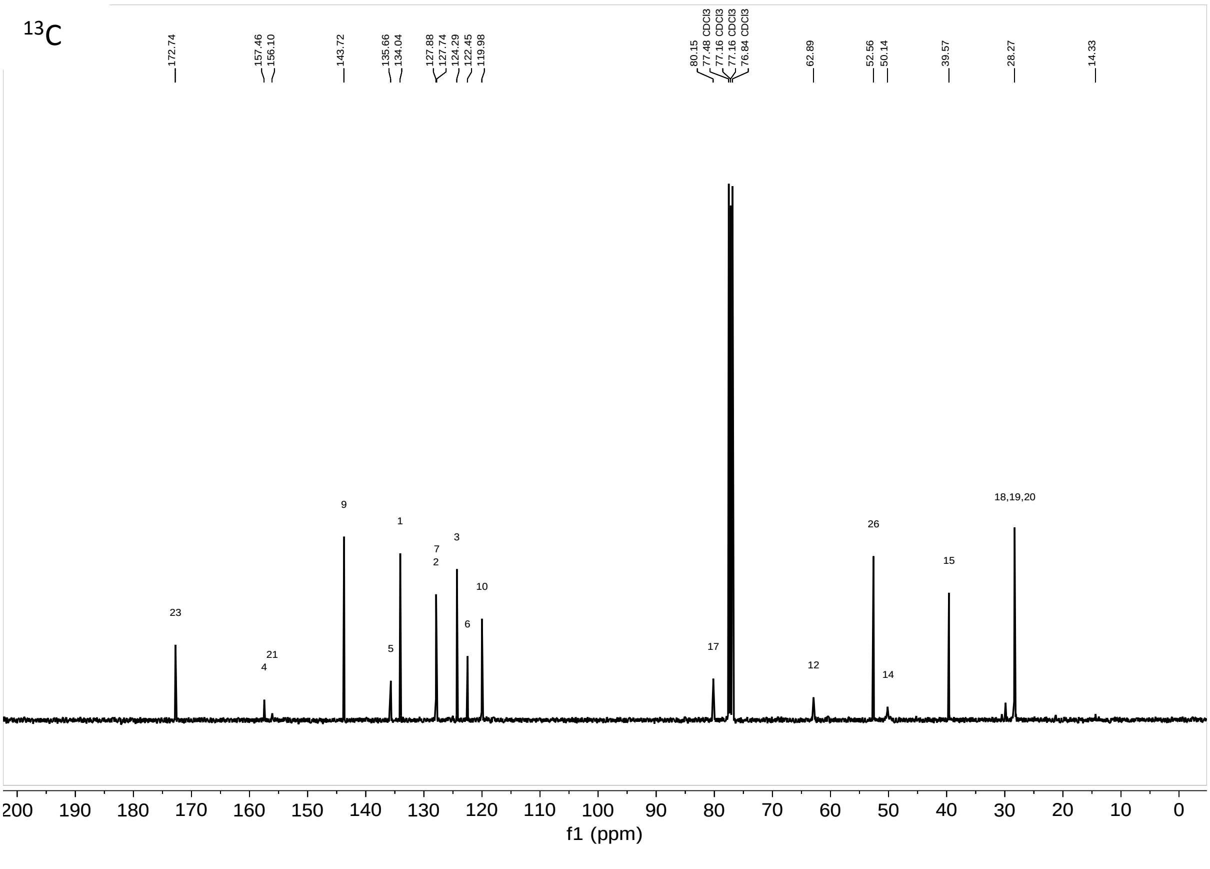

$^{1}H/^{1}H$ COSY (zero filling at 2048 (2K))

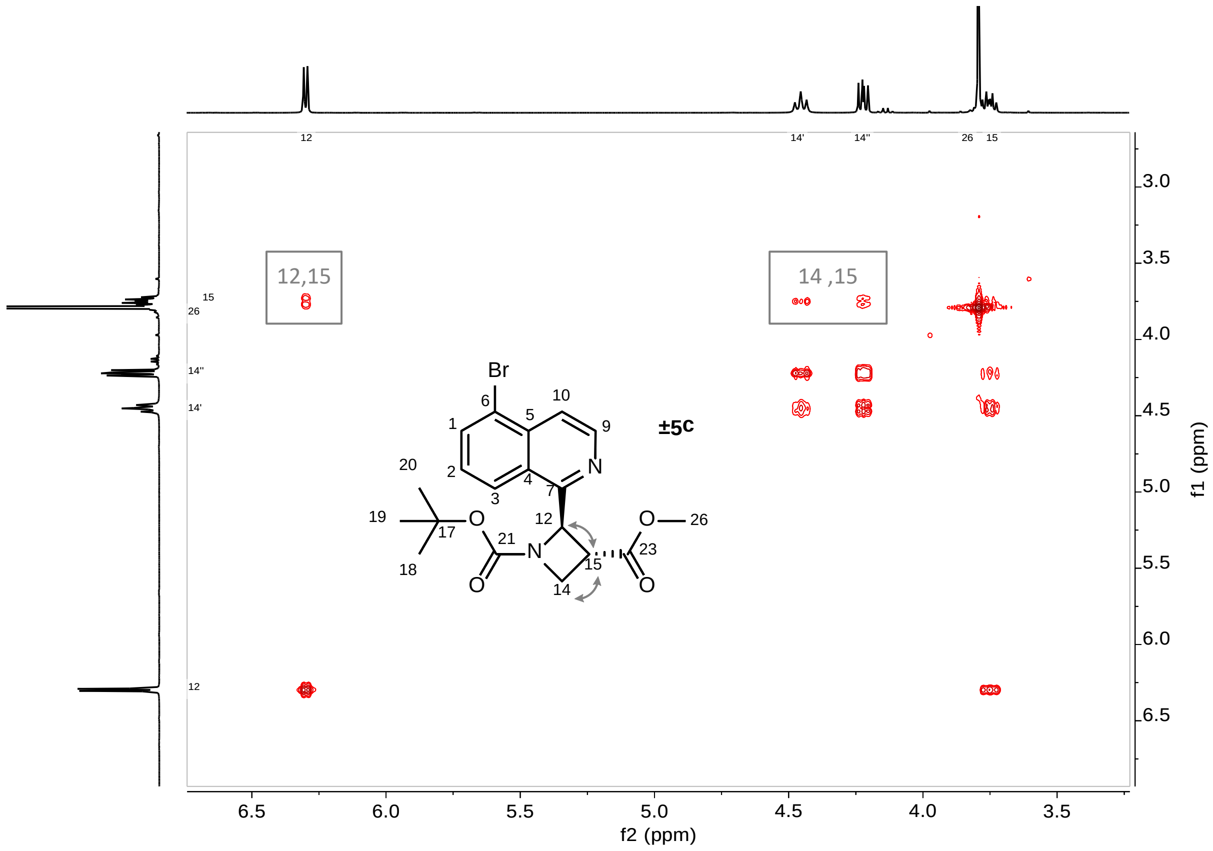

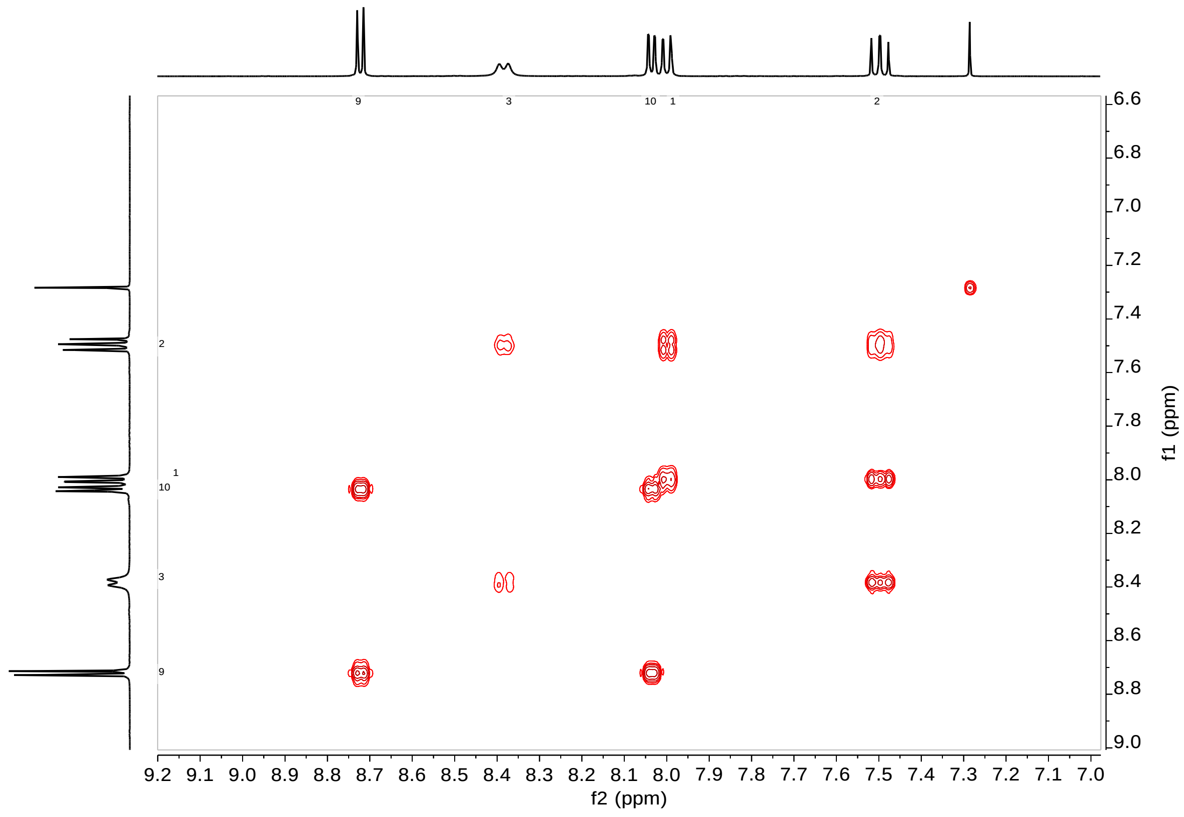

$^{1}H/^{13}C$ HSQC (zero filling at 8192 (8K))

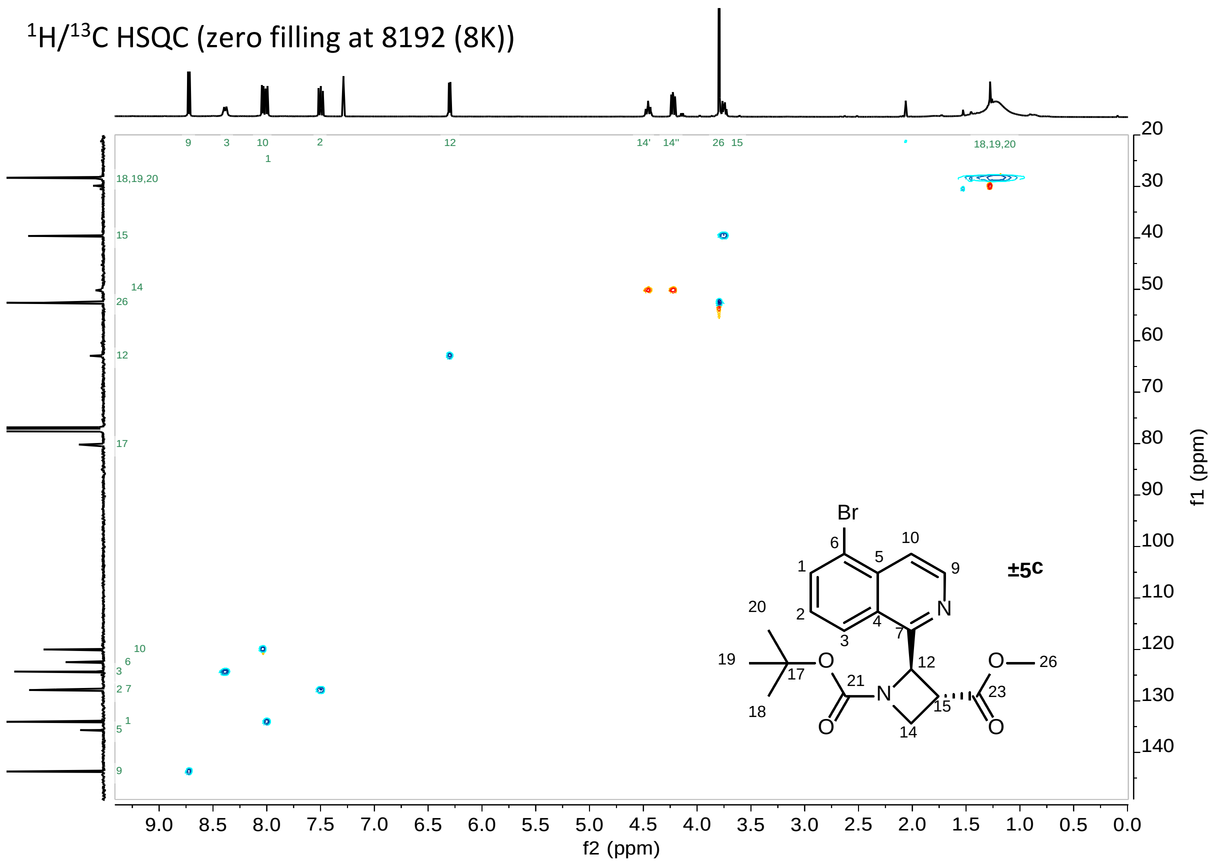

$^{1}H/^{1}H$ ROESY (zero filling at 2048 (2K))

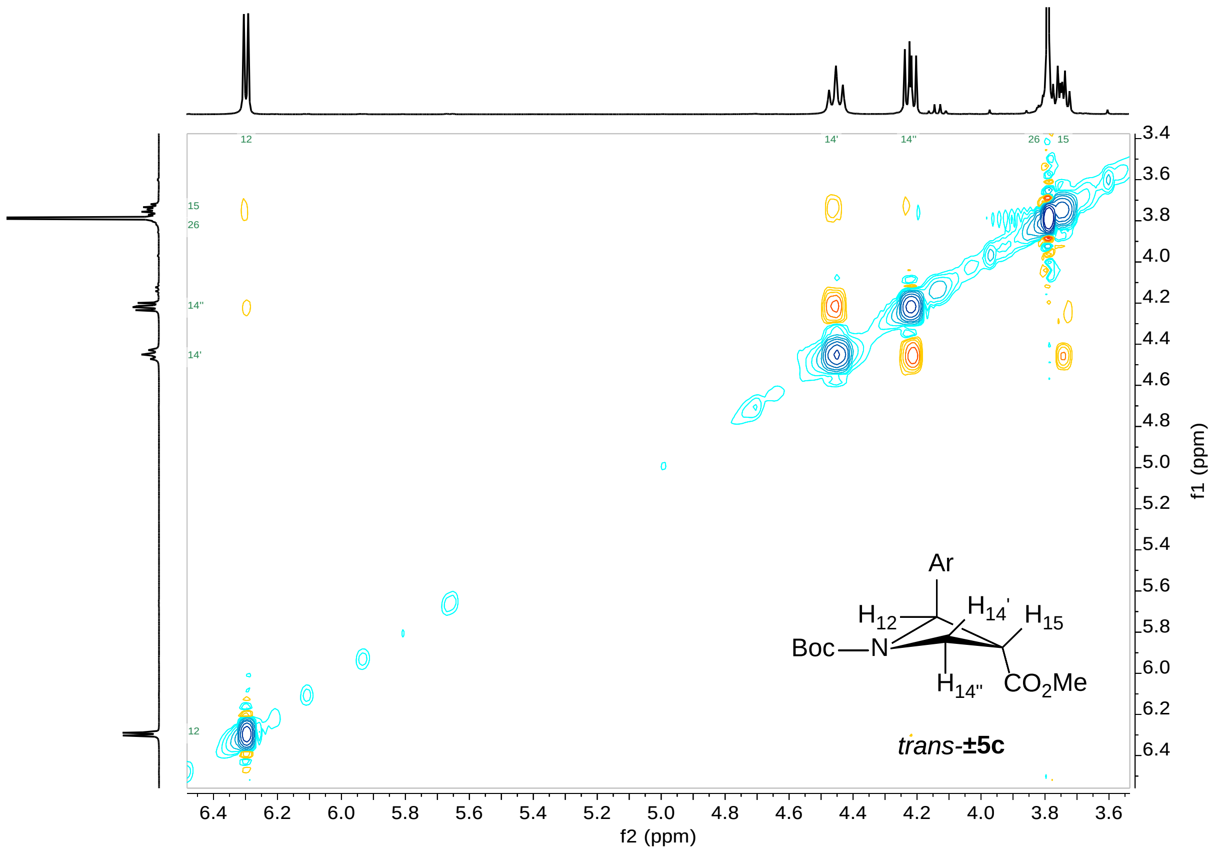

(±)-tert-Butyl 3-(pyridin-4-yl)morpholine-4-carboxylate (±)-5m

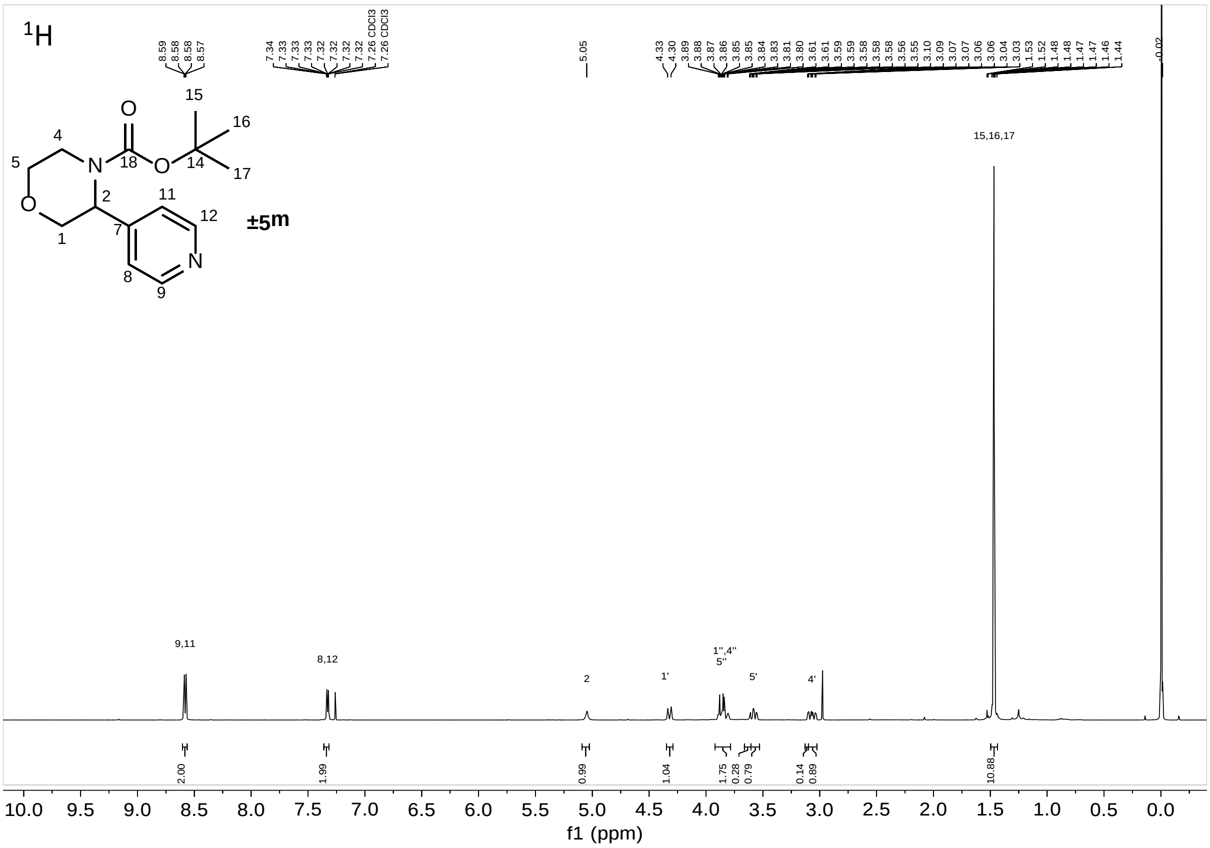

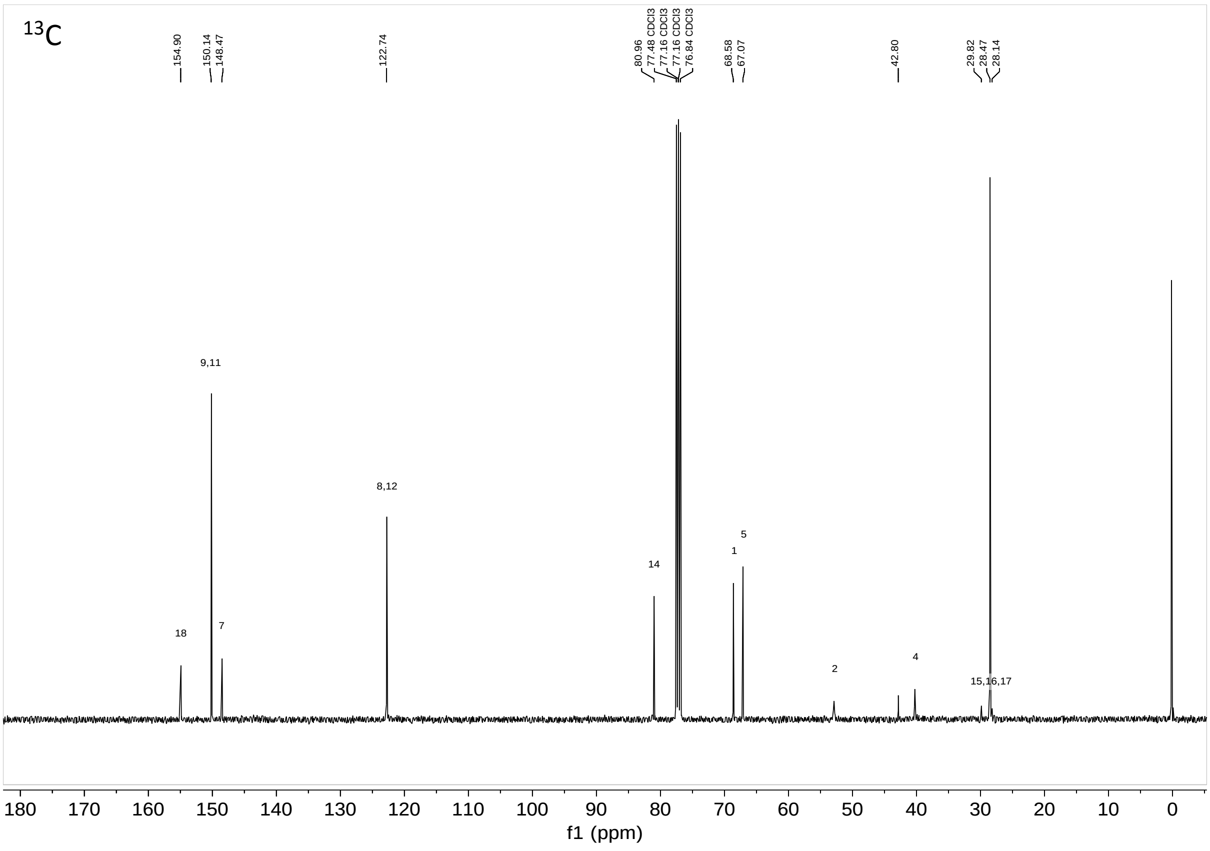

(±)-tert-Butyl 3-(pyridazin-4-yl)morpholine-4-carboxylate (±)-5n

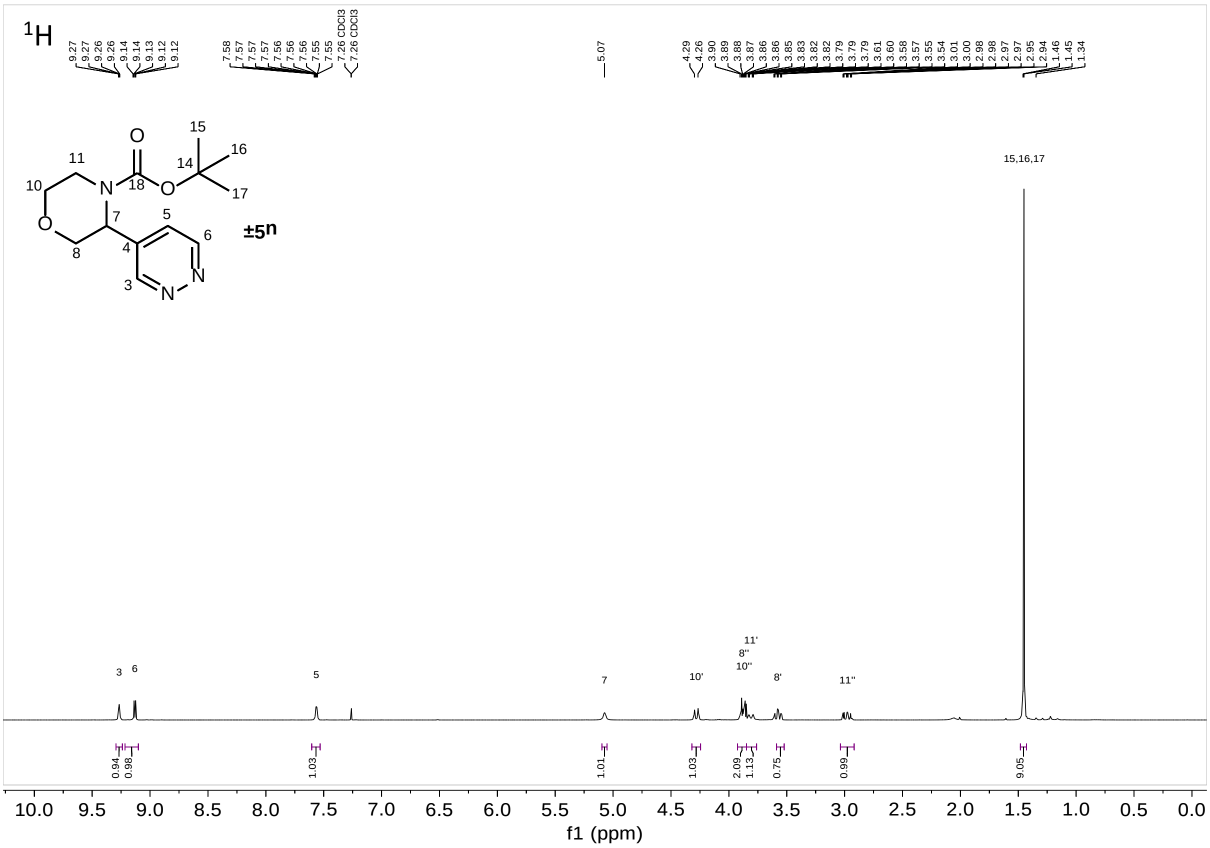

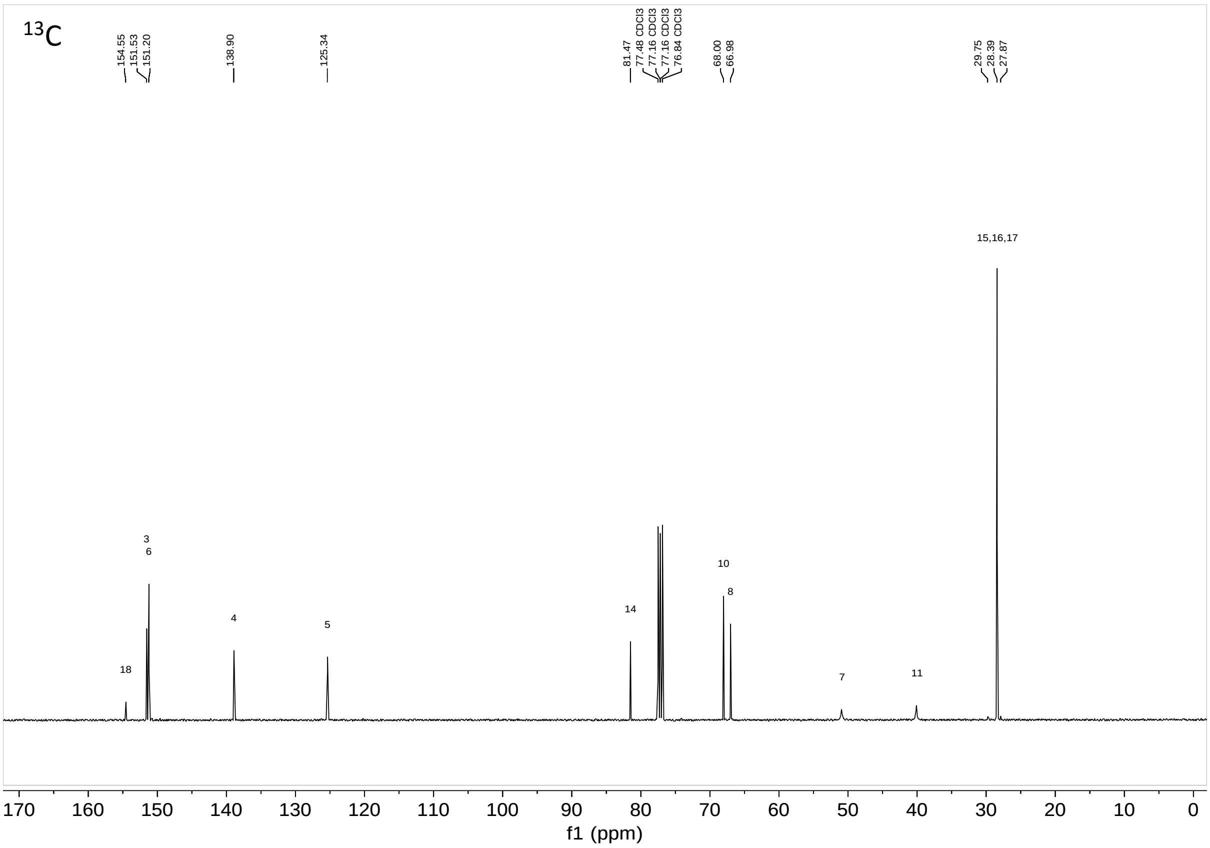

$^1H/^1H$ COSY (zero filling at 1024 (1K))

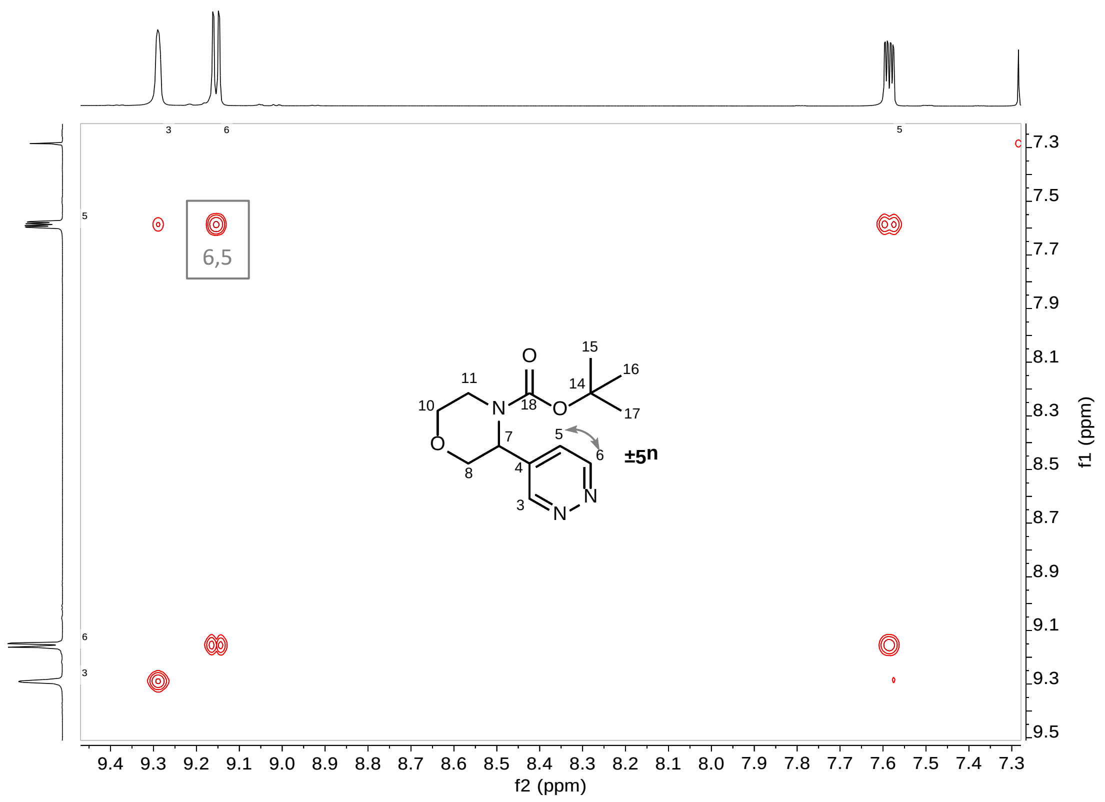

$^1H/^{13}C$ HSQC (zero filling at 4096 (4K))

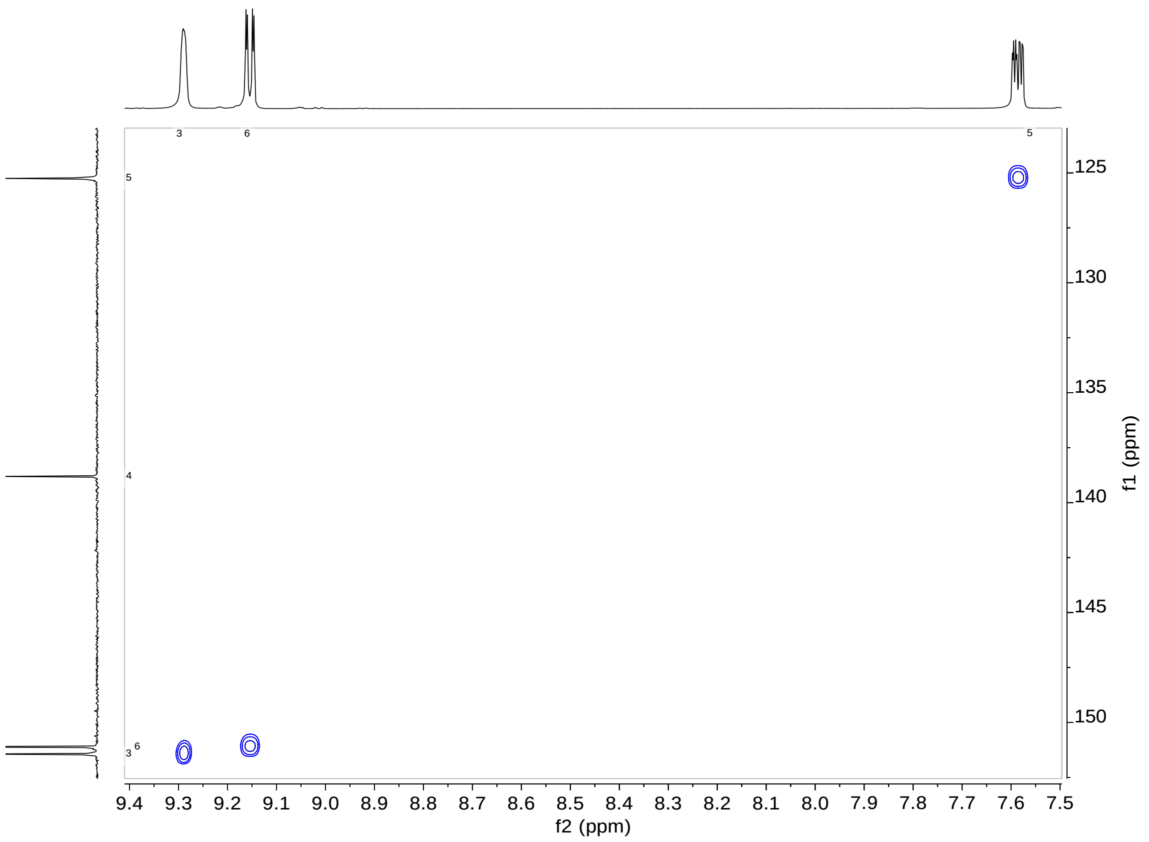

$^{1}H/^{13}C$ HMBC (zero filling at 2048 (2K))

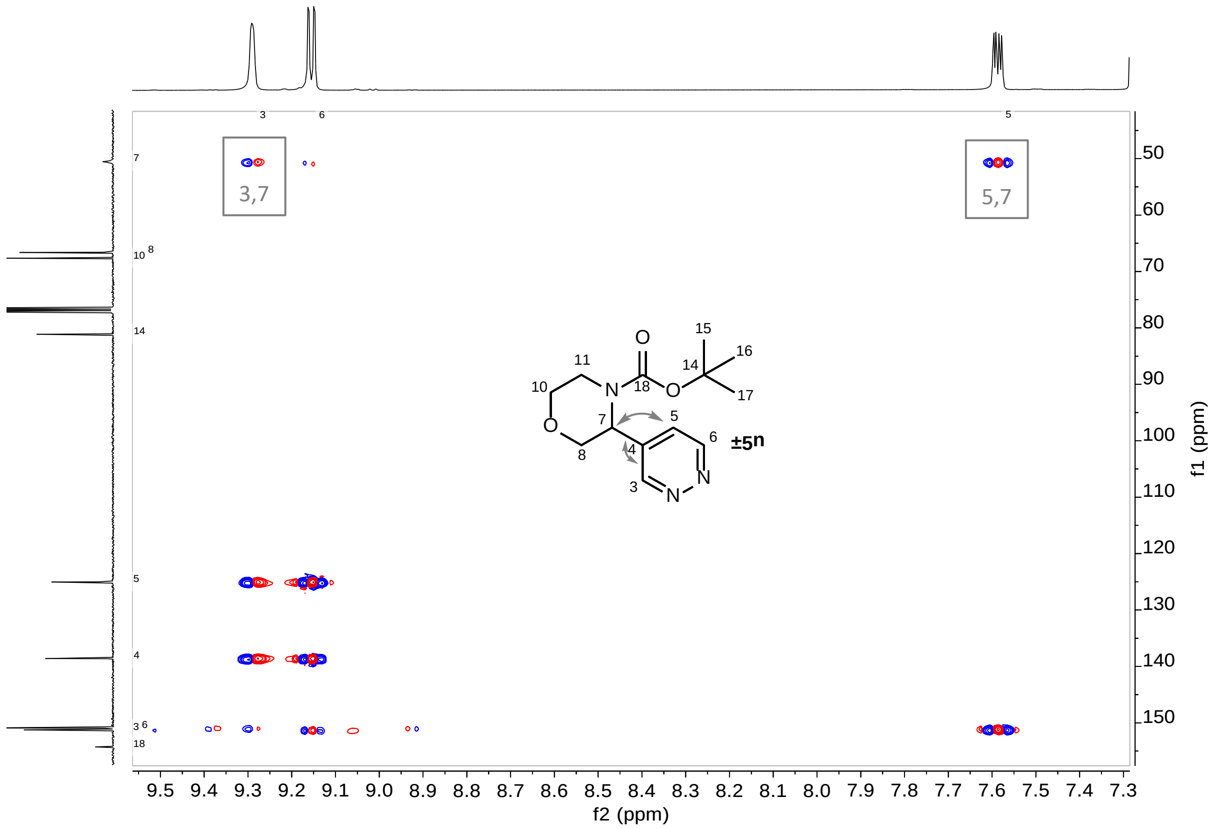

(±)-tert-Butyl 3-(pyrimidin-4-yl)morpholine-4-carboxylate (±)-5o

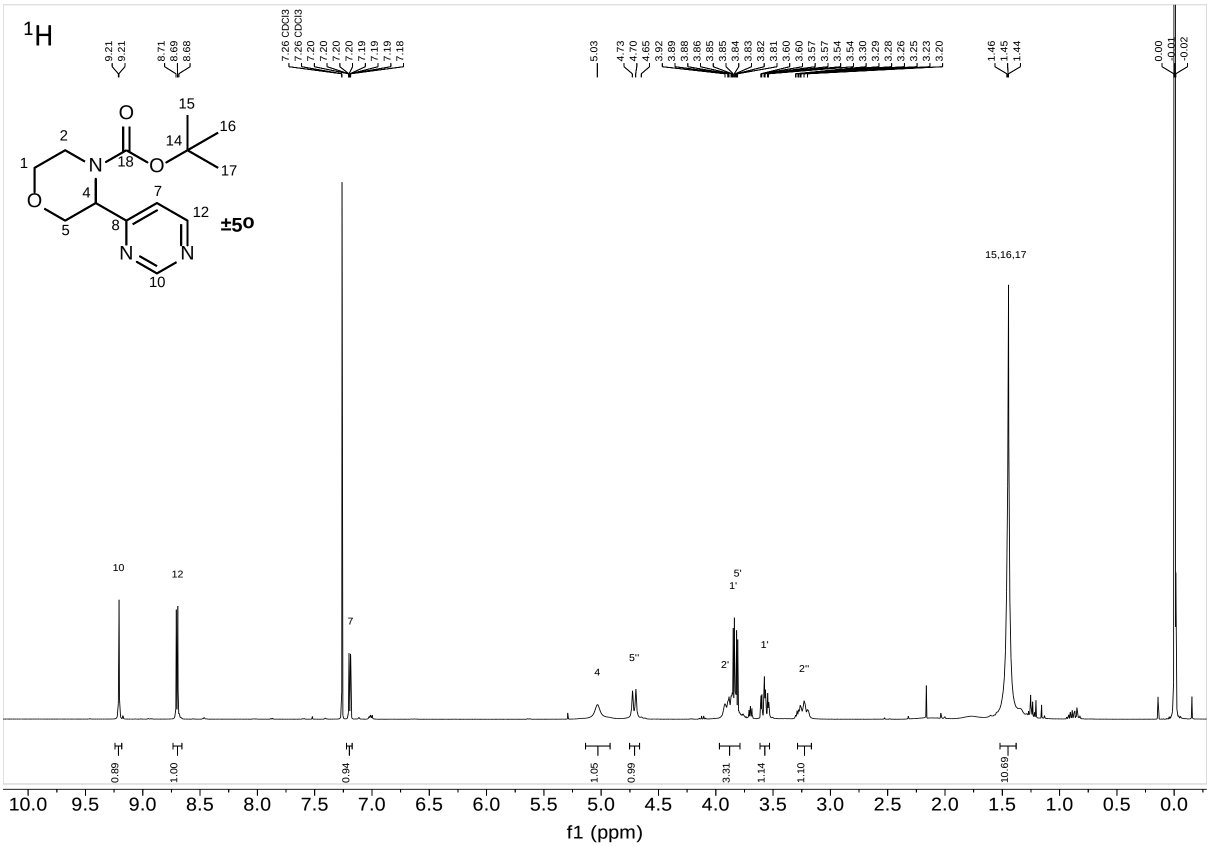

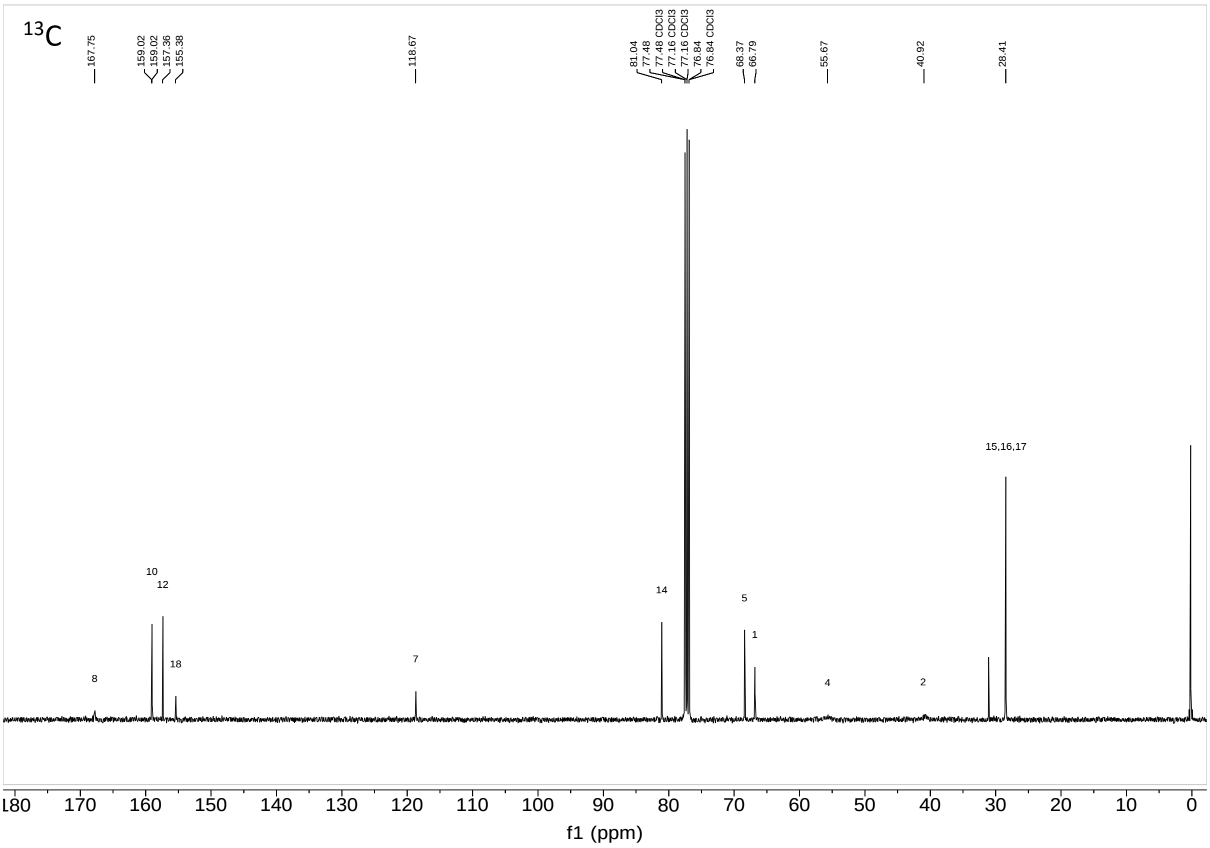

$^{1}H/^{13}C$ COSY (zero filling at 1024 (1K))

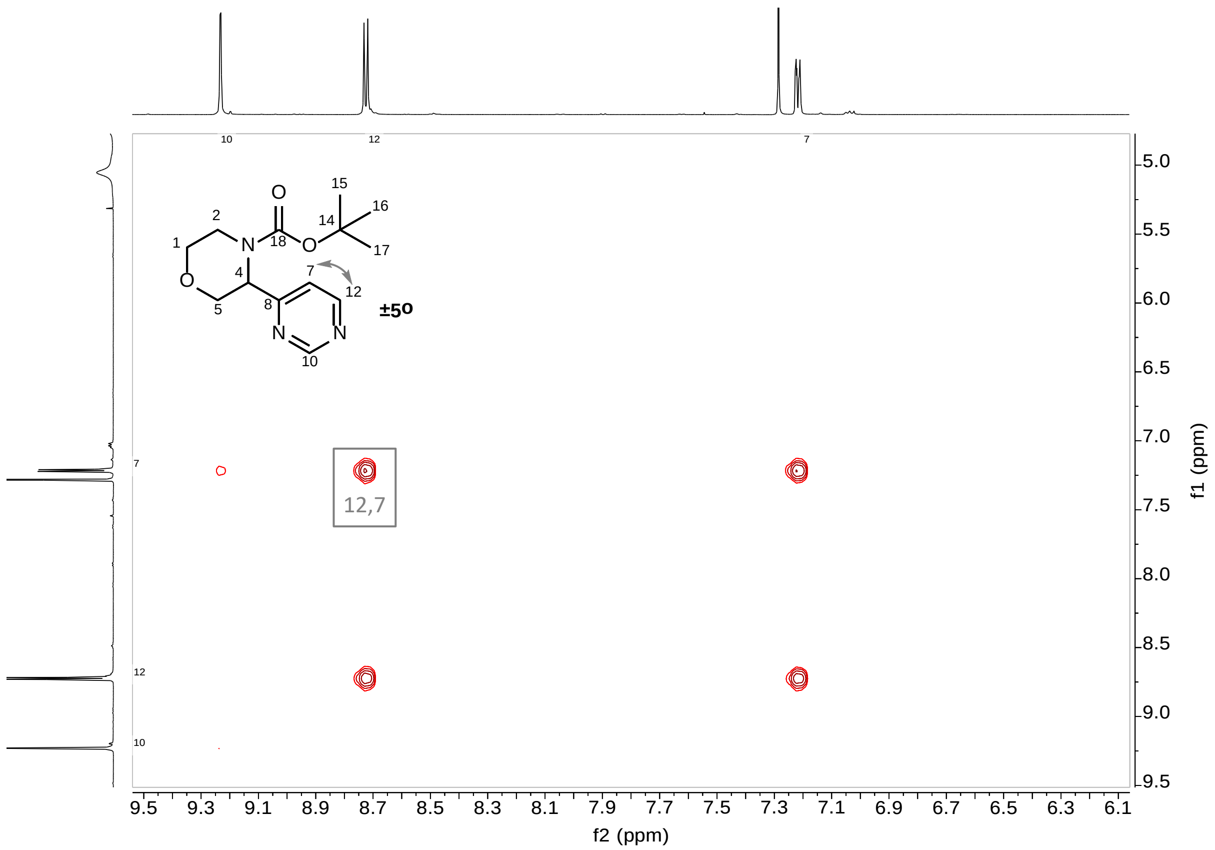

$^{1}H/^{13}C$ HMBC (zero filling at 4096 (4K))

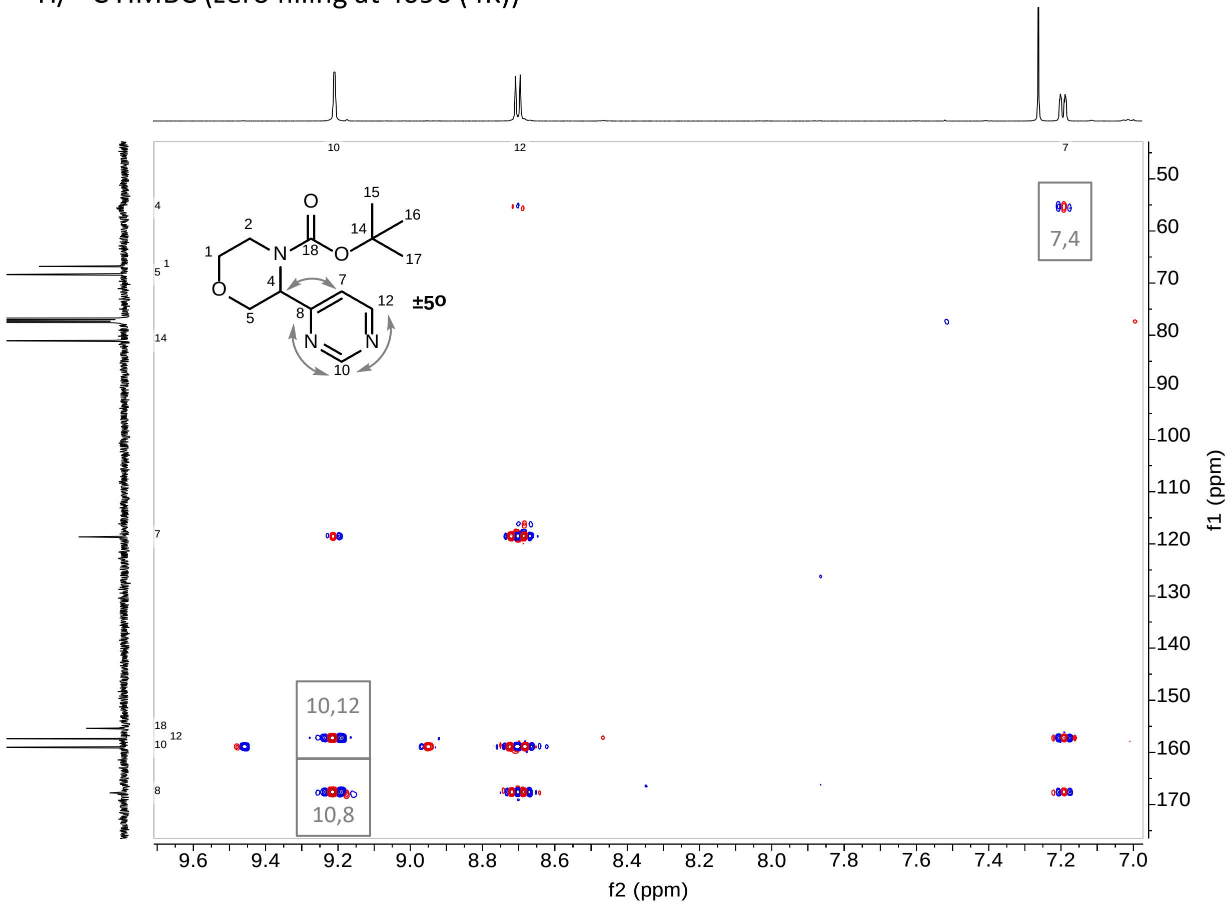

(±)-tert-Butyl 3-(3-fluoropyridin-4-yl)morpholine-4-carboxylate (±)-5r

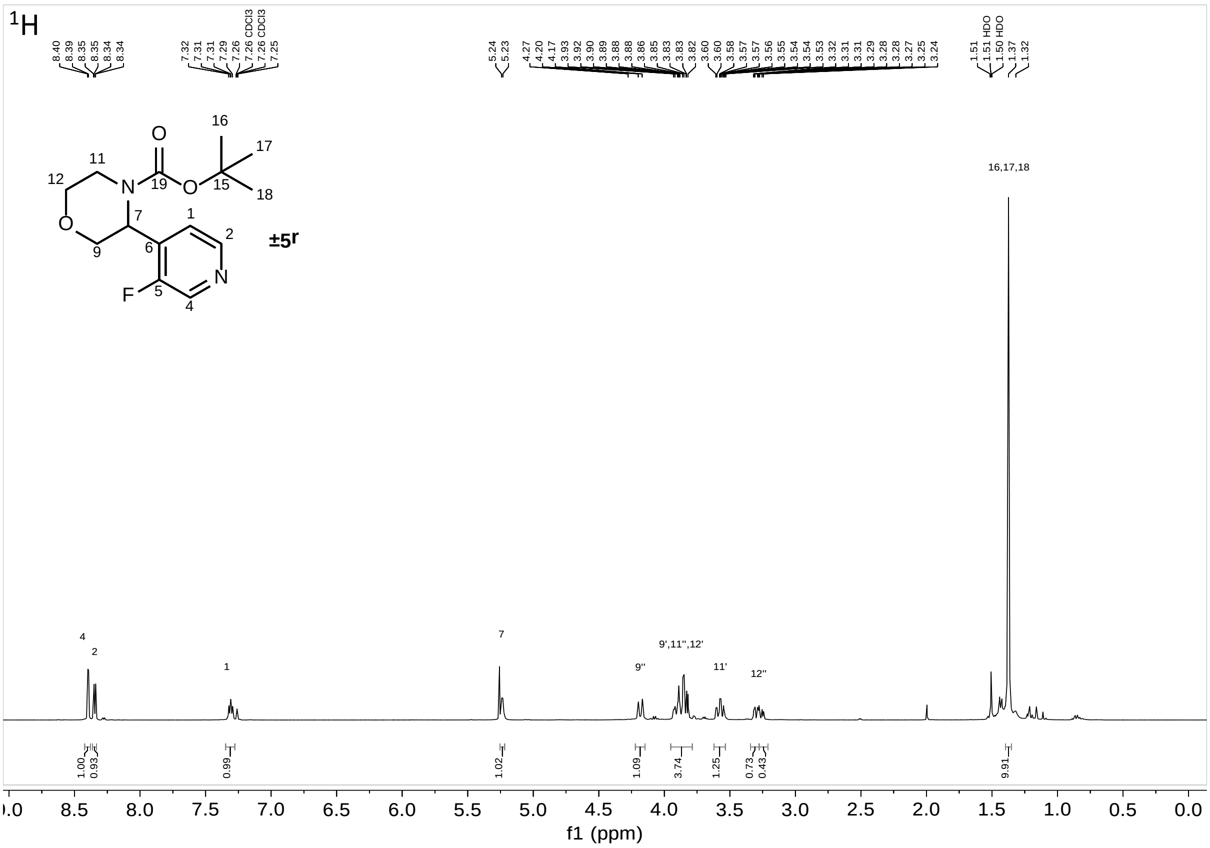

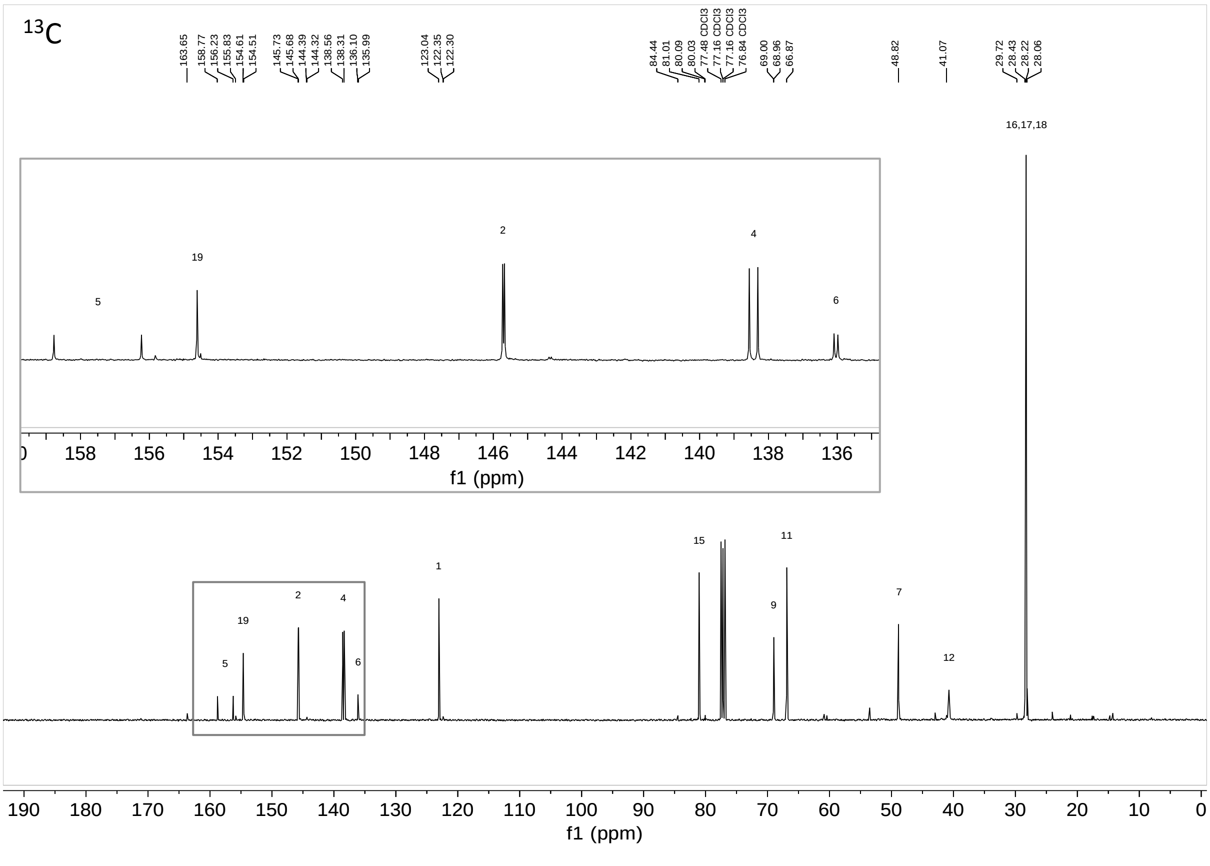

^{1}H/^{1}H COSY (zero filling at 4096 (4K))

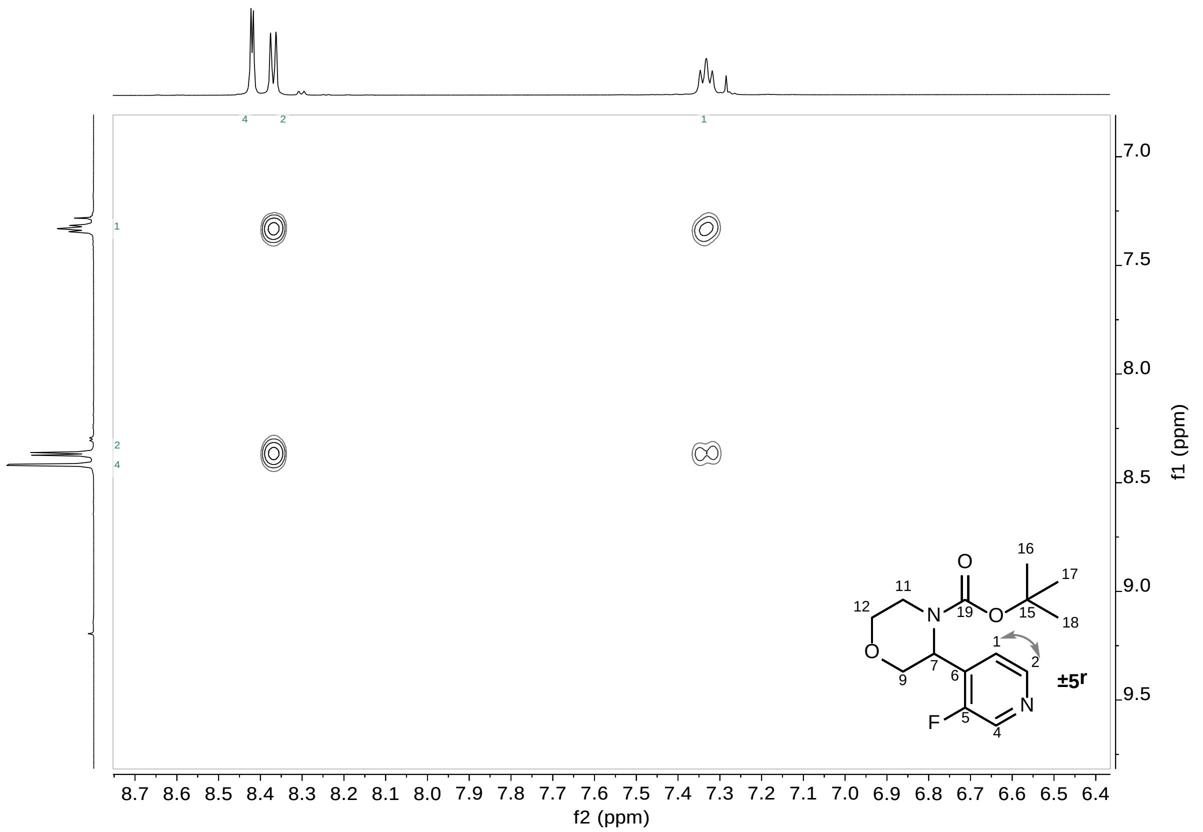

$^1H/^{13}C$ HSQC (zero filling at 4096 (4K))

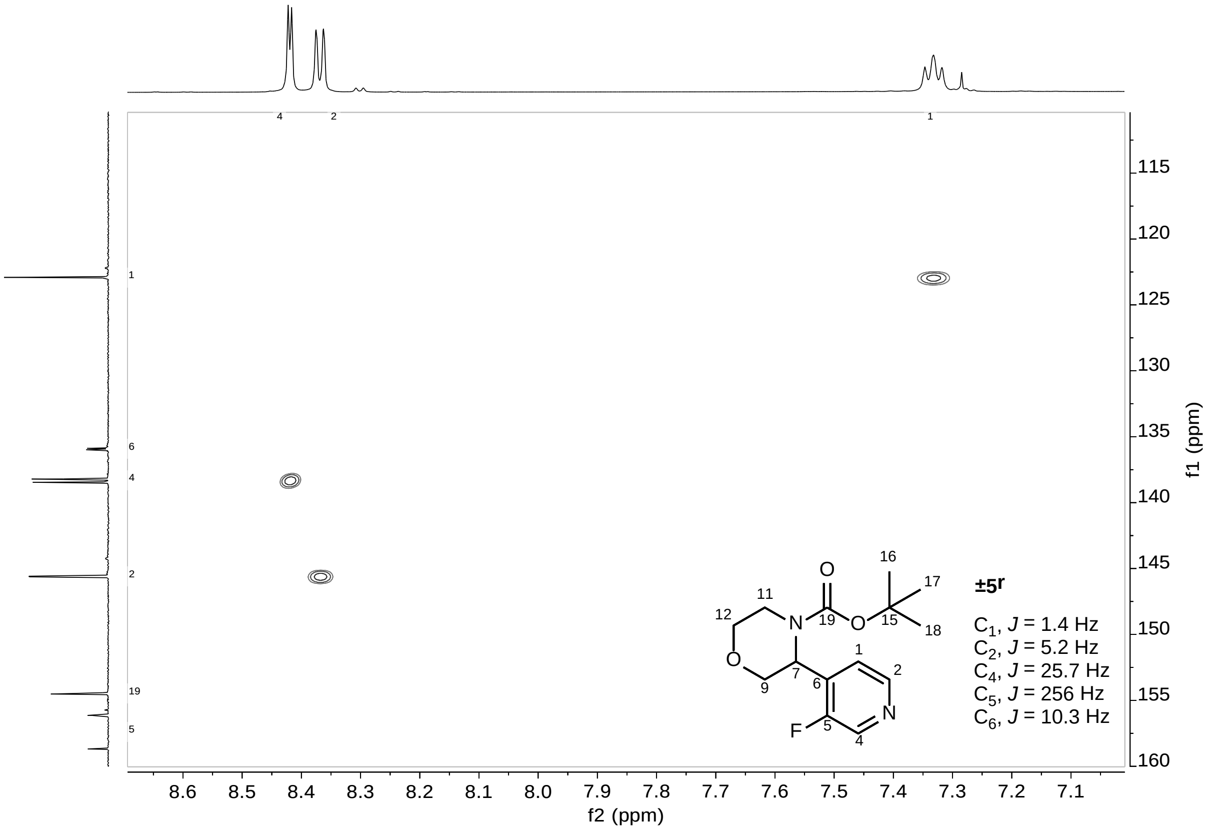

$^1H/^{13}C$ HMBC (zero filling at 8192 (8K))

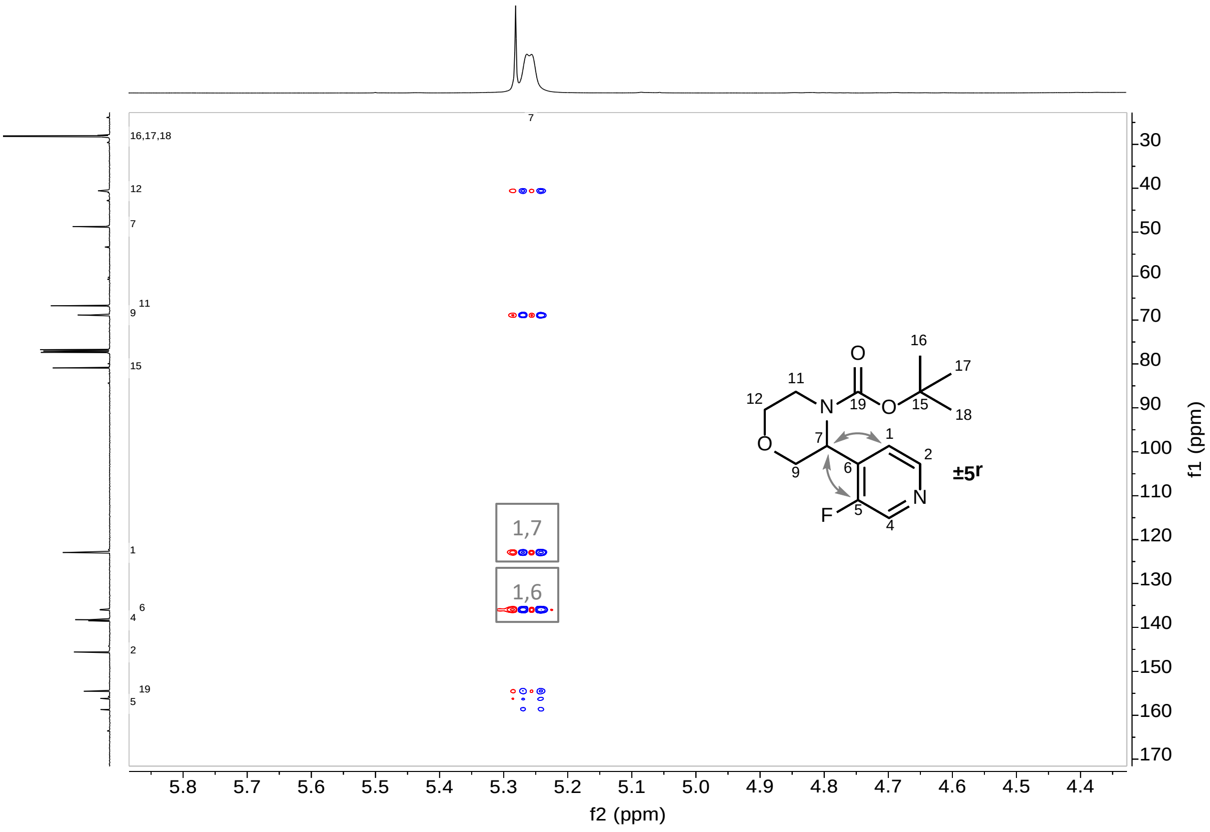

(±)-tert-Butyl 3-[5-(ethoxycarbonyl)pyridin-2-yl]morpholine-4-carboxylate (±)-5s

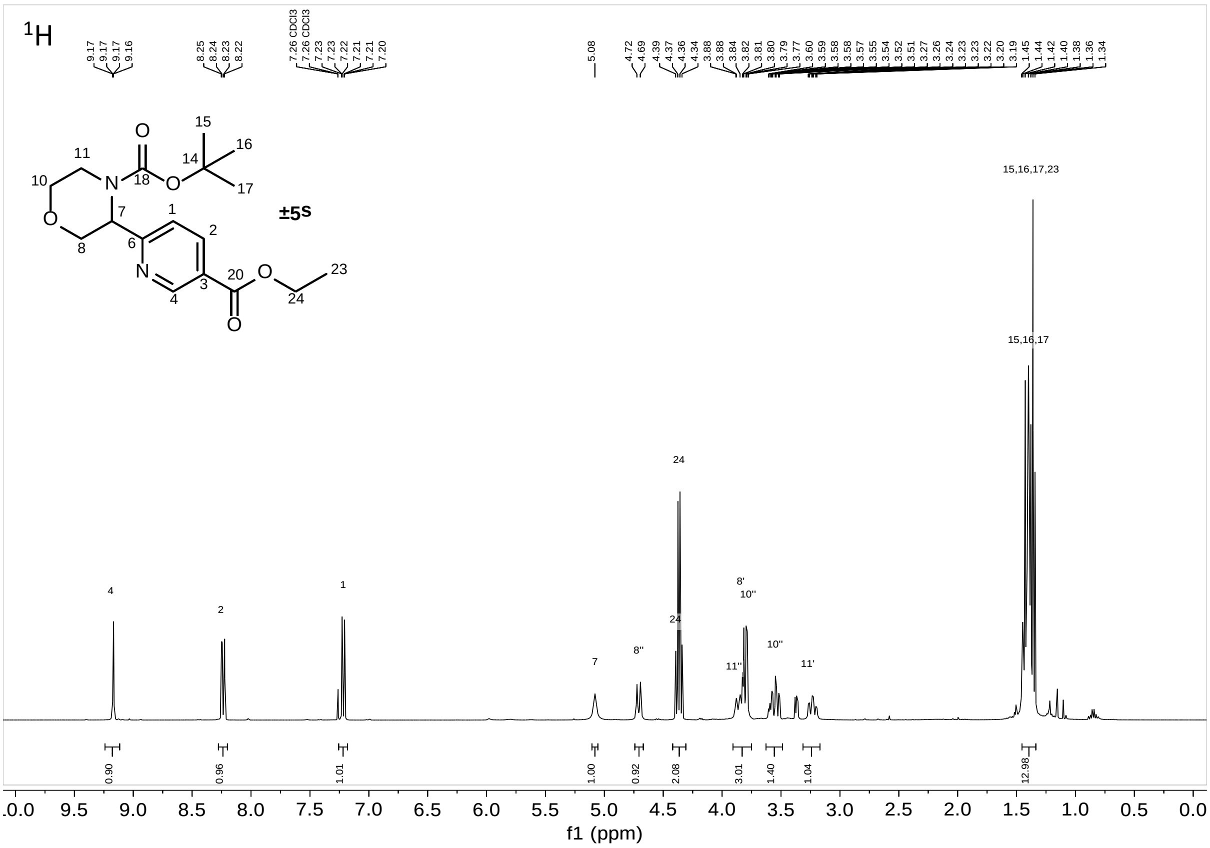

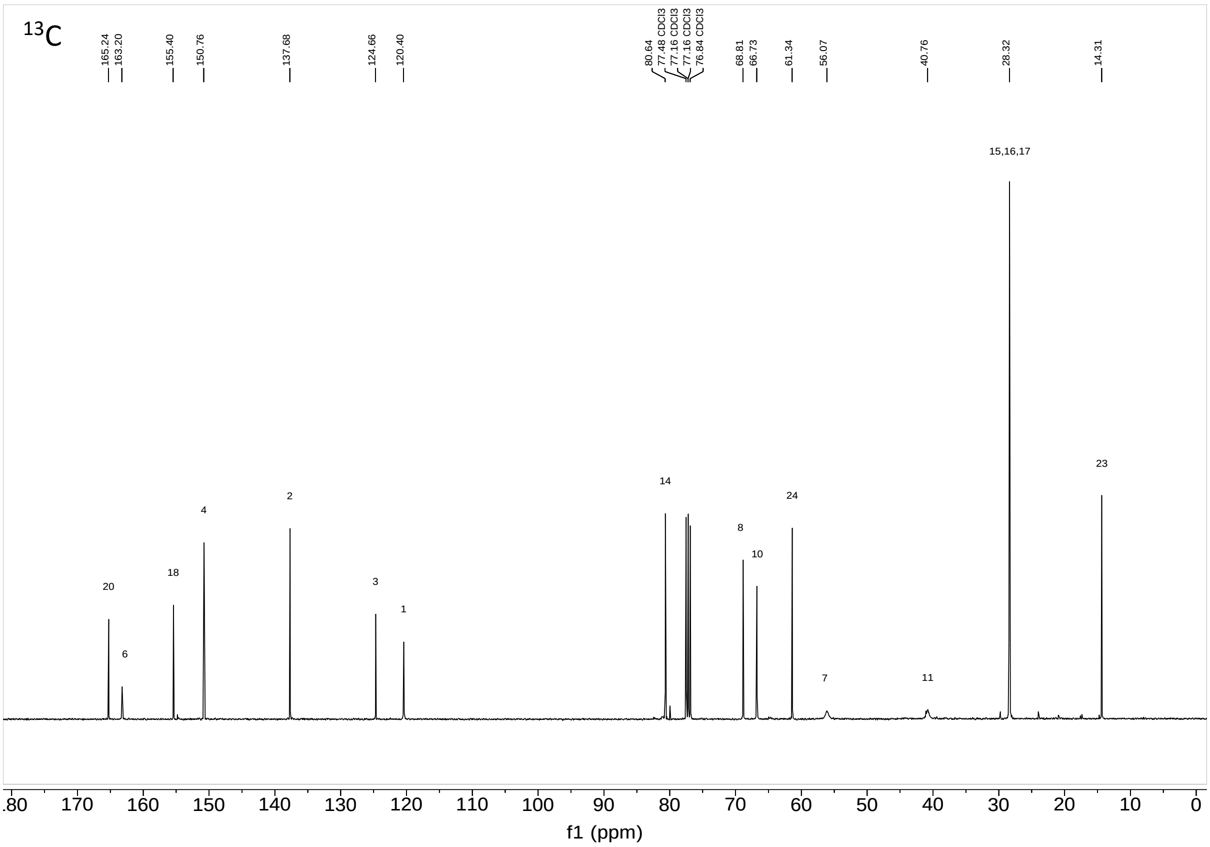

$^{1}H/^{13}C$ HSQC (zero filling at 4096 (4K))

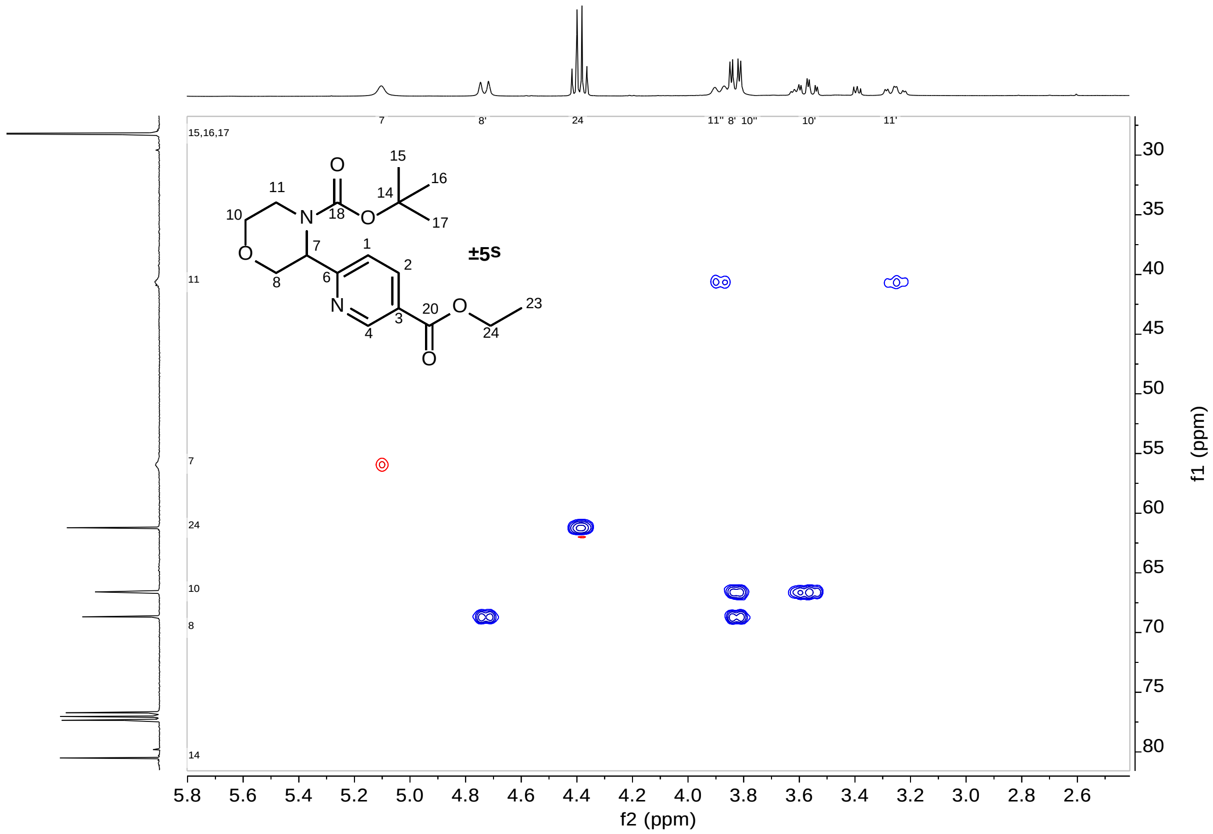

$^{1}H/^{13}C$ HMBC (zero filling at 4096 (4K))

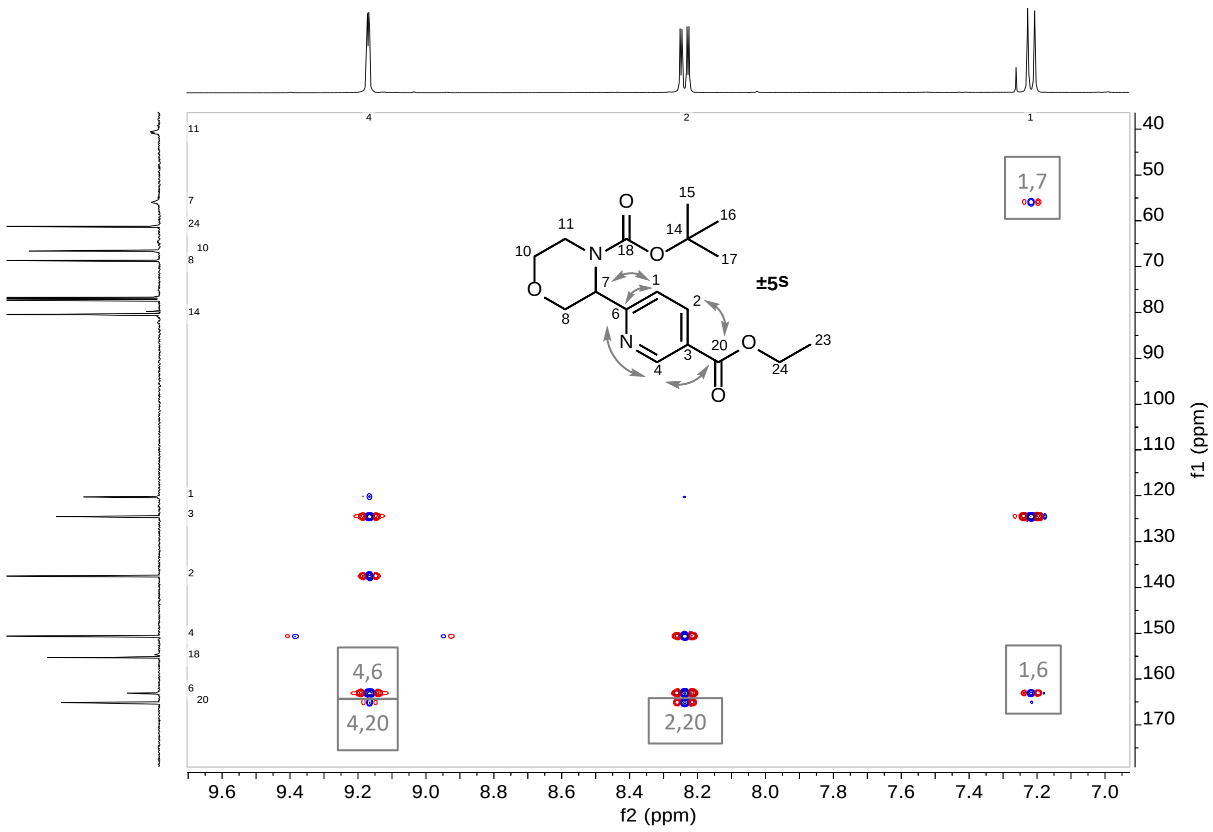

(±)-tert-butyl 3-(3-fluoropyrazin-2-yl)morpholine-4-carboxylate (±)-5t

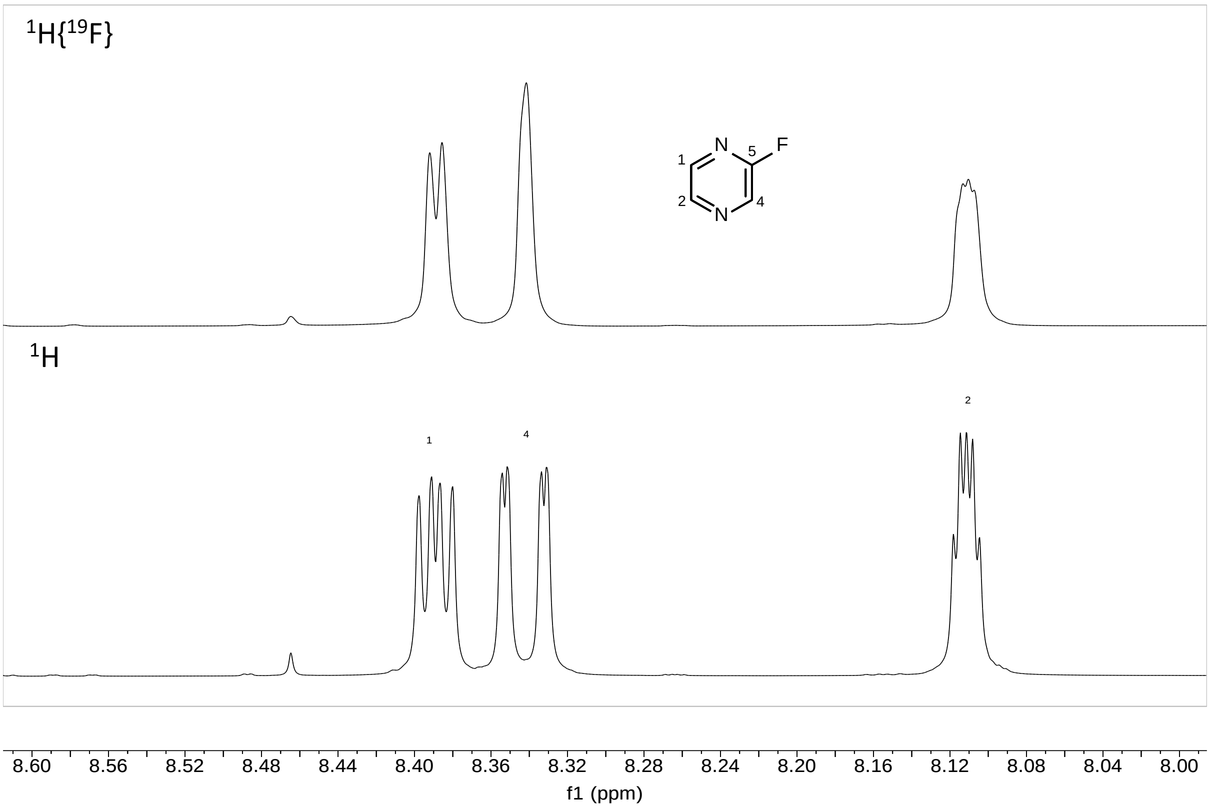

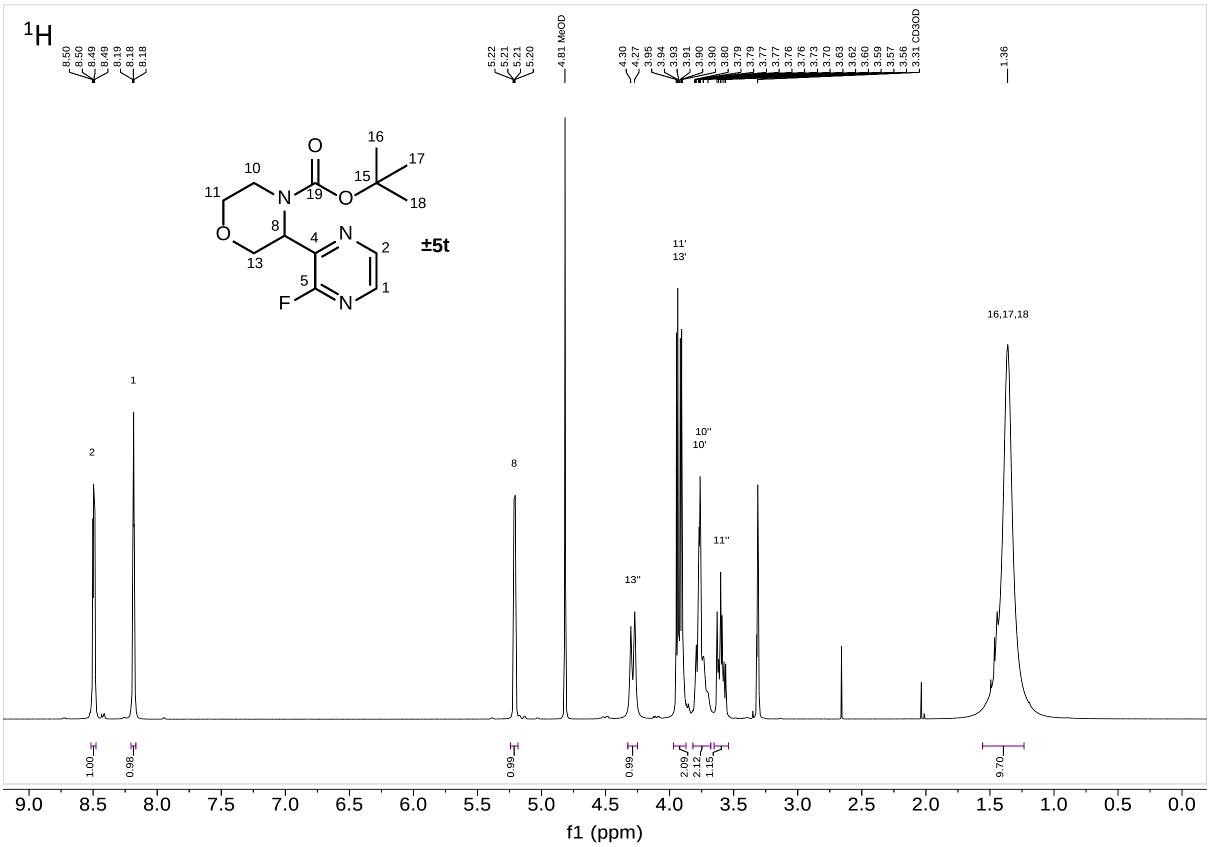

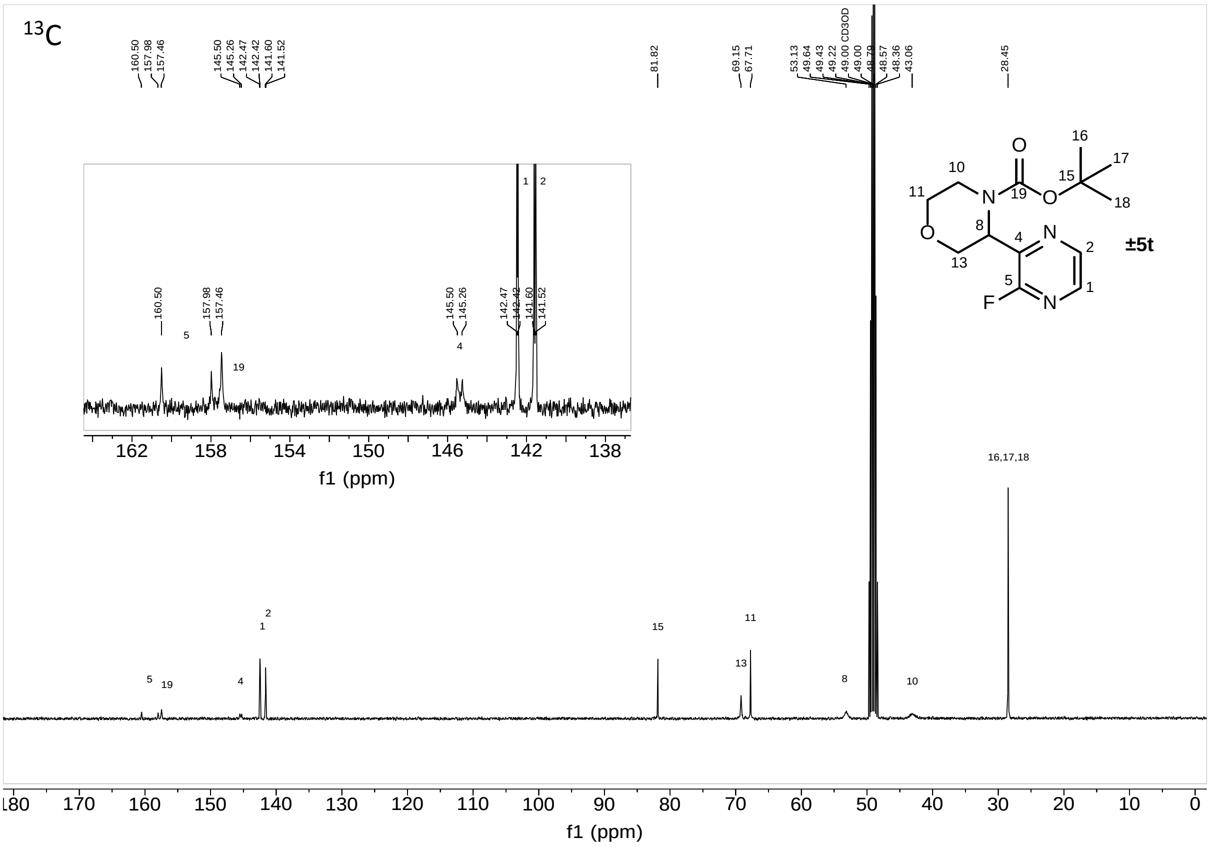
13C
±5t
16,17,18
f1 (ppm)

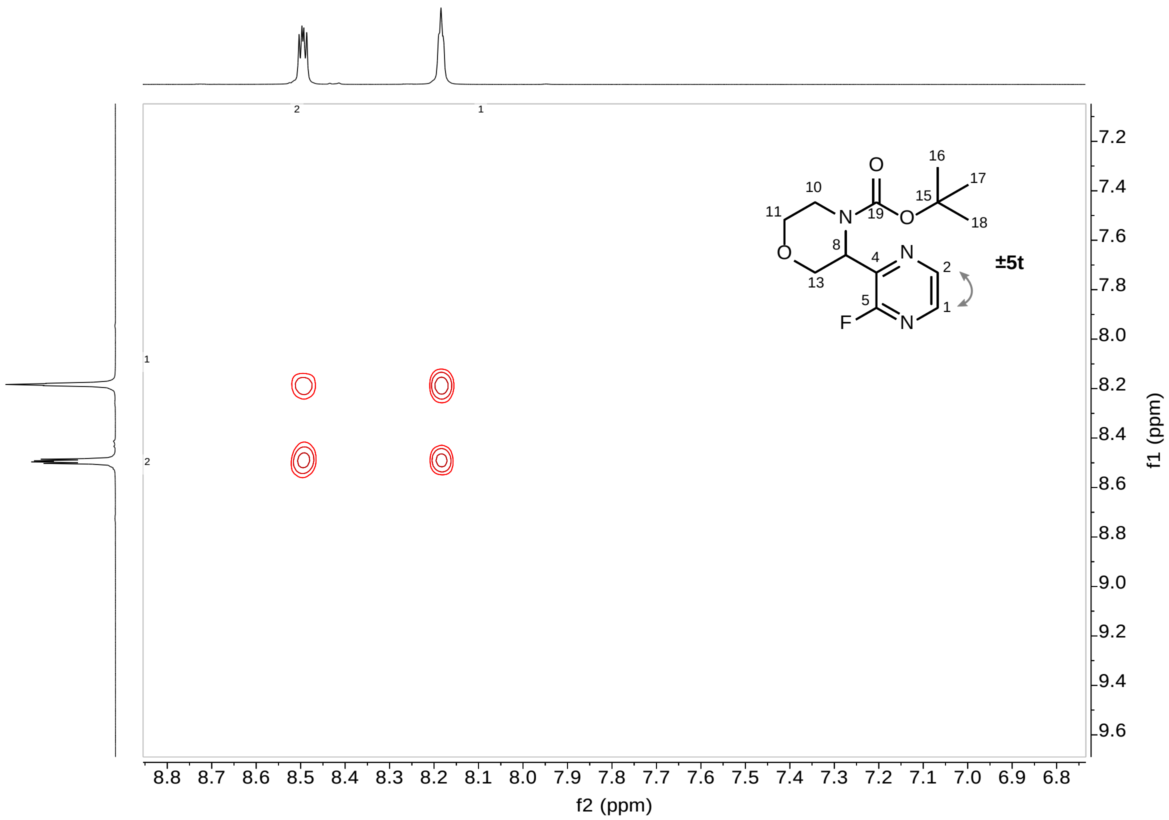
±5t
f2 (ppm)
f1 (ppm)

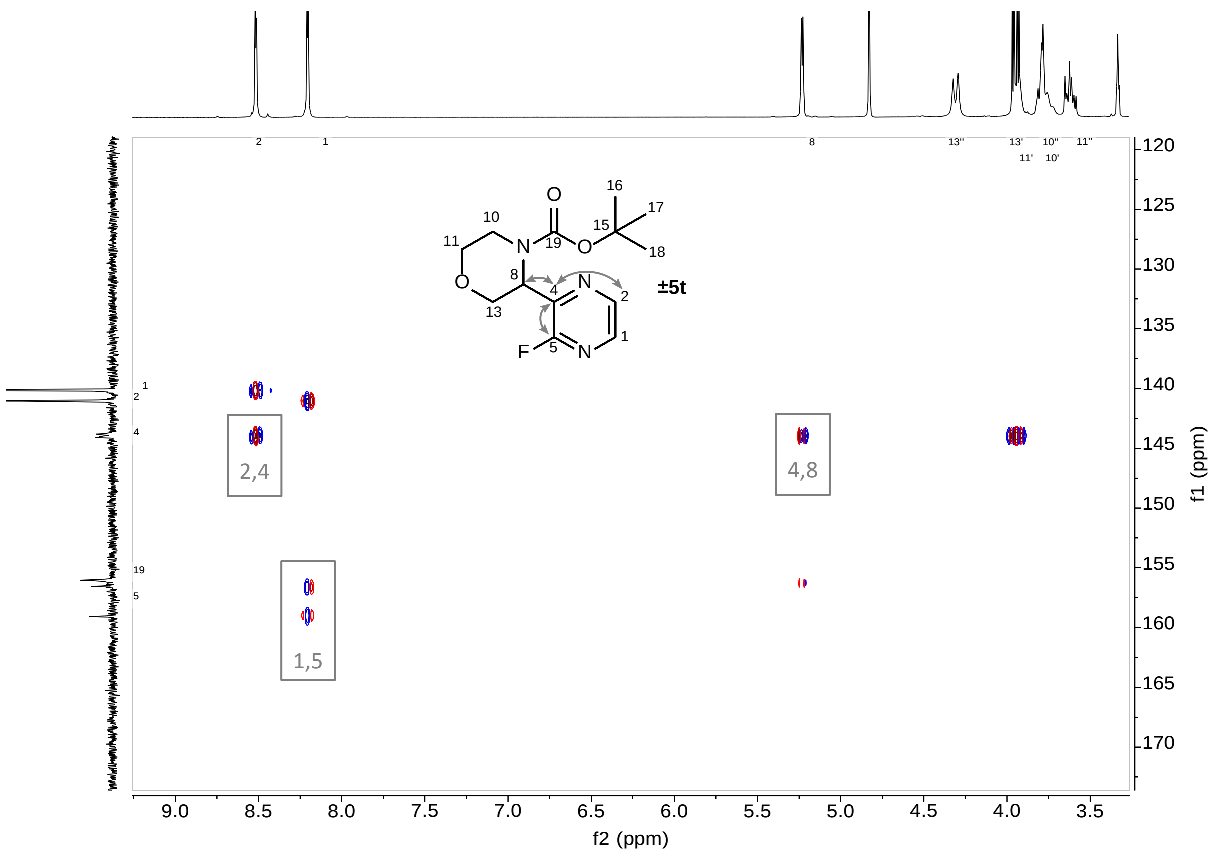

±5t
2,4
4,8
1,5
f2 (ppm)
f1 (ppm)

Liquid Handler Protocols

Andrew Alliance®

Source plate dosing

Andrew Alliance

Protocol
New protocol

Protocol

	Protocol status	Description
	VALID	-
	Author	Email
	Rachel Grainger	Rachel.grainger@astx.com

Created	Last modified	Printed
Aug 01, 2017 at 12:18 UTC-01:00	Aug 07, 2018 at 12:06 UTC-01:00	Aug 07, 2018 at 12:07 UTC-01:00
Filename	Software version	
1804_084.anp	Andrew Lab (v1.5.7)	
Pipette set in use	Microplate pipetting template	
1000G - Gilson PIPETMAN Classic™ P100, P1000	Column by column	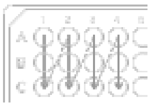

Your material

21 × 2.0 mL conical µtube

- ☐ "A1": A1 (1.2 mL)

  Microtube for centrifugation - spherical bottom, cylindrical shape and integrated flip cap (also called Eppendorf tube)

- ☐ "DMSO": DMSO (1.5 mL)

  Microtube for centrifugation - spherical bottom, cylindrical shape and integrated flip cap (also called Eppendorf tube)

- ☐ "B1": B1 (1.2 mL)

  Microtube for centrifugation - spherical bottom, cylindrical shape and integrated flip cap (also called Eppendorf tube)

- ☐ "D1": D1 (1.2 mL)

  Microtube for centrifugation - spherical bottom, cylindrical shape and integrated flip cap (also called Eppendorf tube)

- ☐ "E1": E1 (1.2 mL)

Microtube for centrifugation - spherical bottom, cylindrical shape and integrated flip cap (also called Eppendorf tube)

- [ ] "C1": C1 (1 mL)

Microtube for centrifugation - spherical bottom, cylindrical shape and integrated flip cap (also called Eppendorf tube)

- [ ] "C3": C1 (1 mL)

Microtube for centrifugation - spherical bottom, cylindrical shape and integrated flip cap (also called Eppendorf tube)

- [ ] "C5": C1 (1 mL)

Microtube for centrifugation - spherical bottom, cylindrical shape and integrated flip cap (also called Eppendorf tube)

- [ ] "C6": C1 (1 mL)

Microtube for centrifugation - spherical bottom, cylindrical shape and integrated flip cap (also called Eppendorf tube)

- [ ] "C7": C1 (1 mL)

Microtube for centrifugation - spherical bottom, cylindrical shape and integrated flip cap (also called Eppendorf tube)

- [ ] "C8": C1 (1 mL)

Microtube for centrifugation - spherical bottom, cylindrical shape and integrated flip cap (also called Eppendorf tube)

- [ ] "C11": C1 (1 mL)

Microtube for centrifugation - spherical bottom, cylindrical shape and integrated flip cap (also called Eppendorf tube)

- [ ] "C13": C1 (1 mL)

Microtube for centrifugation - spherical bottom, cylindrical shape and integrated flip cap (also called Eppendorf tube)

- [ ] "C15": C1 (1 mL)

Microtube for centrifugation - spherical bottom, cylindrical shape and integrated flip cap (also called Eppendorf tube)

- [ ] "C16": C1 (1 mL)

Microtube for centrifugation - spherical bottom, cylindrical shape and integrated flip cap (also called Eppendorf tube)

- [ ] "C2": C1 (1 mL)

Microtube for centrifugation - spherical bottom, cylindrical shape and integrated flip cap (also called Eppendorf tube)

- [ ] "C4": C1 (1 mL)

Microtube for centrifugation - spherical bottom, cylindrical shape and integrated flip cap (also called Eppendorf tube)

- [ ] "C9": C1 (1 mL)

Microtube for centrifugation - spherical bottom, cylindrical shape and integrated flip cap (also called Eppendorf tube)

- [ ] "C10": C1 (1 mL)

Microtube for centrifugation - spherical bottom, cylindrical shape and integrated flip cap (also called Eppendorf tube)

- [ ] "C12": C1 (1 mL)

Microtube for centrifugation - spherical bottom, cylindrical shape and integrated flip cap (also called Eppendorf tube)

- [ ] "C14": C1 (1 mL)

Microtube for centrifugation - spherical bottom, cylindrical shape and integrated flip cap (also called Eppendorf tube)

1 × 384-well plate

- [ ] "384-well plate #1": Empty

Generic microplate with 384 wells. This consumable describes typical square-shaped wells of capacity below 120 uL - with exclusion of 384-wells PCR microplates

1 × Waste

2 × Tips

- [ ] 16 × D200
- [ ] 5 × D1000

2 × Pipettes

- [ ] Gilson PIPETMAN Classic™ P100
- [ ] Gilson PIPETMAN Classic™ P1000

Protocol steps

☐	1	Dispense 60 µL from 2.0 mL conical µtube "A1" to 384-well plate "384-well plate #1": wells A23:P23<br>- Operation: Repetitive mode<br>- Do not change tip between pipetting<br>- Pipetting from Liquid level (Source) / Pipetting on-the-fly (Destination)
☐	2	Dispense 60 µL from 2.0 mL conical µtube "DMSO" to 384-well plate "384-well plate #1": wells A24:P24<br>- Operation: Repetitive mode<br>- Do not change tip between pipetting<br>- Pipetting from Liquid level (Source) / Pipetting on-the-fly (Destination)
☐	3	Dispense 60 µL from 2.0 mL conical µtube "B1" to 384-well plate "384-well plate #1": wells A22:P22

		- Operation: Repetitive mode<br>- Do not change tip between pipetting<br>- Pipetting from Liquid level (Source) / Pipetting on-the-fly (Destination)
☐	4	Dispense 60 µL from 2.0 mL conical µtube "D1" to 384-well plate "384-well plate #1": wells A21:P21<br>- Operation: Repetitive mode<br>- Do not change tip between pipetting<br>- Pipetting from Liquid level (Source) / Pipetting on-the-fly (Destination)
☐	5	Dispense 60 µL from 2.0 mL conical µtube "E1" to 384-well plate "384-well plate #1": wells A20:P20<br>- Operation: Repetitive mode<br>- Do not change tip between pipetting<br>- Pipetting from Liquid level (Source) / Pipetting on-the-fly (Destination)
☐	6	Dispense 60 µL from 2.0 mL conical µtube "C1" to 384-well plate "384-well plate #1": well A19<br>- Pipetting from Liquid level (Source)
☐	7	Dispense 60 µL from 2.0 mL conical µtube "C2" to 384-well plate "384-well plate #1": well B19<br>- Pipetting from Liquid level (Source)
☐	8	Dispense 60 µL from 2.0 mL conical µtube "C3" to 384-well plate "384-well plate #1": well C19<br>- Pipetting from Liquid level (Source)
☐	9	Dispense 60 µL from 2.0 mL conical µtube "C4" to 384-well plate "384-well plate #1": well D19<br>- Pipetting from Liquid level (Source)
☐	10	Dispense 60 µL from 2.0 mL conical µtube "C5" to 384-well plate "384-well plate #1": well E19<br>- Pipetting from Liquid level (Source)
☐	11	Dispense 60 µL from 2.0 mL conical µtube "C6" to 384-well plate "384-well plate #1": well F19<br>- Pipetting from Liquid level (Source)
☐	12	Dispense 60 µL from 2.0 mL conical µtube "C7" to 384-well plate "384-well plate #1": well G19<br>- Pipetting from Liquid level (Source)
☐	13	Dispense 60 µL from 2.0 mL conical µtube "C8" to 384-well plate "384-well plate #1": well H19<br>- Pipetting from Liquid level (Source)

☐	14	Dispense 60 µL from 2.0 mL conical µtube "C9" to 384-well plate "384-well plate #1": well I19<br>- Pipetting from Liquid level (Source)
☐	15	Dispense 60 µL from 2.0 mL conical µtube "C10" to 384-well plate "384-well plate #1": well J19<br>- Pipetting from Liquid level (Source)
☐	16	Dispense 60 µL from 2.0 mL conical µtube "C11" to 384-well plate "384-well plate #1": well K19<br>- Pipetting from Liquid level (Source)
☐	17	Dispense 60 µL from 2.0 mL conical µtube "C12" to 384-well plate "384-well plate #1": well L19<br>- Pipetting from Liquid level (Source)
☐	18	Dispense 60 µL from 2.0 mL conical µtube "C13" to 384-well plate "384-well plate #1": well M19<br>- Pipetting from Liquid level (Source)
☐	19	Dispense 60 µL from 2.0 mL conical µtube "C14" to 384-well plate "384-well plate #1": well N19<br>- Pipetting from Liquid level (Source)
☐	20	Dispense 60 µL from 2.0 mL conical µtube "C15" to 384-well plate "384-well plate #1": well O19<br>- Pipetting from Liquid level (Source)
☐	21	Dispense 60 µL from 2.0 mL conical µtube "C16" to 384-well plate "384-well plate #1": well P19<br>- Pipetting from Liquid level (Source)

This protocol was designed with Andrew Lab by Andrew Alliance (www.andrewalliance.com)

Mosquito TTP Labtech

Reaction plate dosing

5 position deck

Position:

1: [no plate]

2: Corning 1536 COC white

3: Greiner 384 (v bottom)

4: Corning 1536 COC white

5: [no plate]

Home tape

Aspirate 1000 nL from (P3, C23, R1, S1), well volume 50 µL

Dispense 500 nL to (P2, C1, R1, S1), well volume 0 µL

Dispense 500 nL to (P2, C3, R1, S1), well volume 0 µL

Aspirate 1000 nL from (P3, C23, R1, S1), well volume 49 µL

Dispense 500 nL to (P2, C5, R1, S1), well volume 0 µL

Dispense 500 nL to (P2, C7, R1, S1), well volume 0 µL

Aspirate 1000 nL from (P3, C23, R1, S1), well volume 48 µL

Dispense 500 nL to (P2, C9, R1, S1), well volume 0 µL

Dispense 500 nL to (P2, C11, R1, S1), well volume 0 µL

Aspirate 1000 nL from (P3, C23, R1, S1), well volume 47 µL

Dispense 500 nL to (P2, C1, R2, S1), well volume 0 µL

Dispense 500 nL to (P2, C3, R2, S1), well volume 0 µL

Aspirate 1000 nL from (P3, C23, R1, S1), well volume 46 µL

Dispense 500 nL to (P2, C5, R2, S1), well volume 0 µL

Dispense 500 nL to (P2, C7, R2, S1), well volume 0 µL

Aspirate 1000 nL from (P3, C23, R1, S1), well volume 45 µL

Dispense 500 nL to (P2, C9, R2, S1), well volume 0 µL

Dispense 500 nL to (P2, C11, R2, S1), well volume 0 µL

Aspirate 1000 nL from (P3, C23, R1, S1), well volume 44 µL

Dispense 500 nL to (P2, C2, R1, S1), well volume 0 µL

Dispense 500 nL to (P2, C4, R1, S1), well volume 0 µL

Aspirate 1000 nL from (P3, C23, R1, S1), well volume 43 µL

Dispense 500 nL to (P2, C6, R1, S1), well volume 0 µL

Dispense 500 nL to (P2, C8, R1, S1), well volume 0 µL

Aspirate 1000 nL from (P3, C23, R1, S1), well volume 42 µL

Dispense 500 nL to (P2, C10, R1, S1), well volume 0 µL

Dispense 500 nL to (P2, C12, R1, S1), well volume 0 µL

Aspirate 1000 nL from (P3, C23, R1, S1), well volume 41 µL

Dispense 500 nL to (P2, C2, R2, S1), well volume 0 µL

Dispense 500 nL to (P2, C4, R2, S1), well volume 0 µL

Aspirate 1000 nL from (P3, C23, R1, S1), well volume 40 µL

Dispense 500 nL to (P2, C6, R2, S1), well volume 0 µL

Dispense 500 nL to (P2, C8, R2, S1), well volume 0 µL

Aspirate 1000 nL from (P3, C23, R1, S1), well volume 39 µL

Dispense 500 nL to (P2, C10, R2, S1), well volume 0 µL

Dispense 500 nL to (P2, C12, R2, S1), well volume 0 µL

Change pipettes

Aspirate 1000 nL from (P3, C22, R1, S1), well volume 50 µL

Dispense 125 nL to (P2, C1, R1, S1), well volume 0 µL

Dispense 125 nL to (P2, C3, R1, S1), well volume 0 µL

Dispense 125 nL to (P2, C1, R1, S1), well volume 0 µL

Dispense 125 nL to (P2, C3, R1, S1), well volume 0 µL

Dispense 125 nL to (P2, C2, R1, S1), well volume 0 µL

Dispense 125 nL to (P2, C4, R1, S1), well volume 0 µL

Dispense 125 nL to (P2, C2, R1, S1), well volume 0 µL

Dispense 125 nL to (P2, C4, R1, S1), well volume 0 µL

Aspirate 1200 nL from (P3, C22, R1, S1), well volume 49 µL

Dispense 300 nL to (P2, C5, R1, S1), well volume 0 µL

Dispense 300 nL to (P2, C7, R1, S1), well volume 0 µL

Dispense 300 nL to (P2, C5, R1, S1), well volume 0 µL

Dispense 300 nL to (P2, C7, R1, S1), well volume 0 µL

Aspirate 1200 nL from (P3, C22, R1, S1), well volume 47.8 µL

Dispense 300 nL to (P2, C6, R1, S1), well volume 0 µL

Dispense 300 nL to (P2, C8, R1, S1), well volume 0 µL

Dispense 300 nL to (P2, C6, R1, S1), well volume 0 µL

Dispense 300 nL to (P2, C8, R1, S1), well volume 0 µL

Aspirate 1000 nL from (P3, C22, R1, S1), well volume 46.6 µL

Dispense 500 nL to (P2, C9, R1, S1), well volume 0 µL

Dispense 500 nL to (P2, C11, R1, S1), well volume 0 µL

Aspirate 1000 nL from (P3, C22, R1, S1), well volume 45.6 µL

Dispense 500 nL to (P2, C9, R1, S1), well volume 0 µL

Dispense 500 nL to (P2, C11, R1, S1), well volume 0 µL

Aspirate 1000 nL from (P3, C22, R1, S1), well volume 44.6 µL

Dispense 500 nL to (P2, C10, R1, S1), well volume 0 µL

Dispense 500 nL to (P2, C12, R1, S1), well volume 0 µL

Aspirate 1000 nL from (P3, C22, R1, S1), well volume 43.6 µL

Dispense 500 nL to (P2, C10, R1, S1), well volume 0 µL

Dispense 500 nL to (P2, C12, R1, S1), well volume 0 µL

Change pipettes

Aspirate 1125 nL from (P3, C24, R1, S1), well volume 50 µL

Dispense 375 nL to (P2, C1, R1, S1), well volume 0 µL

Dispense 375 nL to (P2, C3, R1, S1), well volume 0 µL

Dispense 375 nL to (P2, C1, R1, S1), well volume 0 µL

Aspirate 1125 nL from (P3, C24, R1, S1), well volume 48.875 µL

Dispense 375 nL to (P2, C3, R1, S1), well volume 0 µL

Dispense 375 nL to (P2, C2, R1, S1), well volume 0 µL

Dispense 375 nL to (P2, C4, R1, S1), well volume 0 µL

Aspirate 1150 nL from (P3, C24, R1, S1), well volume 47.75 µL

Dispense 375 nL to (P2, C2, R1, S1), well volume 0 µL

Dispense 375 nL to (P2, C4, R1, S1), well volume 0 µL

Dispense 200 nL to (P2, C5, R1, S1), well volume 0 µL

Dispense 200 nL to (P2, C7, R1, S1), well volume 0 µL

Aspirate 1200 nL from (P3, C24, R1, S1), well volume 46.6 µL

Dispense 200 nL to (P2, C5, R1, S1), well volume 0 µL

Dispense 200 nL to (P2, C7, R1, S1), well volume 0 µL

Dispense 200 nL to (P2, C6, R1, S1), well volume 0 µL

Dispense 200 nL to (P2, C8, R1, S1), well volume 0 µL

Dispense 200 nL to (P2, C6, R1, S1), well volume 0 µL

Dispense 200 nL to (P2, C8, R1, S1), well volume 0 µL

Change pipettes

Aspirate 1000 nL from (P3, C21, R1, S1), well volume 50 µL

Dispense 250 nL to (P2, C1, R1, S1), well volume 0 µL

Dispense 250 nL to (P2, C1, R2, S1), well volume 0 µL

Dispense 250 nL to (P2, C2, R1, S1), well volume 0 µL

Dispense 250 nL to (P2, C2, R2, S1), well volume 0 µL

Aspirate 1000 nL from (P3, C21, R1, S1), well volume 49 µL

Dispense 500 nL to (P2, C3, R1, S1), well volume 0 µL

Dispense 500 nL to (P2, C3, R2, S1), well volume 0 µL

Aspirate 1000 nL from (P3, C21, R1, S1), well volume 48 µL

Dispense 500 nL to (P2, C4, R1, S1), well volume 0 µL

Dispense 500 nL to (P2, C4, R2, S1), well volume 0 µL

Aspirate 1000 nL from (P3, C21, R1, S1), well volume 47 µL

Dispense 250 nL to (P2, C5, R1, S1), well volume 0 µL

Dispense 250 nL to (P2, C5, R2, S1), well volume 0 µL

Dispense 250 nL to (P2, C6, R1, S1), well volume 0 µL

Dispense 250 nL to (P2, C6, R2, S1), well volume 0 µL

Aspirate 1000 nL from (P3, C21, R1, S1), well volume 46 µL

Dispense 500 nL to (P2, C7, R1, S1), well volume 0 µL

Dispense 500 nL to (P2, C7, R2, S1), well volume 0 µL

Aspirate 1000 nL from (P3, C21, R1, S1), well volume 45 µL

Dispense 500 nL to (P2, C8, R1, S1), well volume 0 µL

Dispense 500 nL to (P2, C8, R2, S1), well volume 0 µL

Aspirate 1000 nL from (P3, C21, R1, S1), well volume 44 µL

Dispense 250 nL to (P2, C9, R1, S1), well volume 0 µL

Dispense 250 nL to (P2, C9, R2, S1), well volume 0 µL

Dispense 250 nL to (P2, C10, R1, S1), well volume 0 µL

Dispense 250 nL to (P2, C10, R2, S1), well volume 0 µL

Aspirate 1000 nL from (P3, C21, R1, S1), well volume 43 µL

Dispense 500 nL to (P2, C11, R1, S1), well volume 0 µL

Dispense 500 nL to (P2, C11, R2, S1), well volume 0 µL

Aspirate 1000 nL from (P3, C21, R1, S1), well volume 42 µL

Dispense 500 nL to (P2, C12, R1, S1), well volume 0 µL

Dispense 500 nL to (P2, C12, R2, S1), well volume 0 µL

Change pipettes

Aspirate 1000 nL from (P3, C24, R1, S1), well volume 45.4 µL

Dispense 250 nL to (P2, C1, R1, S1), well volume 0 µL

Dispense 250 nL to (P2, C1, R2, S1), well volume 0 µL

Dispense 250 nL to (P2, C2, R1, S1), well volume 0 µL

Dispense 250 nL to (P2, C2, R2, S1), well volume 0 µL

Aspirate 1000 nL from (P3, C24, R1, S1), well volume 44.4 µL

Dispense 250 nL to (P2, C5, R1, S1), well volume 0 µL

Dispense 250 nL to (P2, C5, R2, S1), well volume 0 µL

Dispense 250 nL to (P2, C6, R1, S1), well volume 0 µL

Dispense 250 nL to (P2, C6, R2, S1), well volume 0 µL

Aspirate 1000 nL from (P3, C24, R1, S1), well volume 43.4 µL

Dispense 250 nL to (P2, C9, R1, S1), well volume 0 µL

Dispense 250 nL to (P2, C9, R2, S1), well volume 0 µL

Dispense 250 nL to (P2, C10, R1, S1), well volume 0 µL

Dispense 250 nL to (P2, C10, R2, S1), well volume 0 µL

Change pipettes

Aspirate 1000 nL from (P3, C20, R1, S1), well volume 50 µL

Dispense 500 nL to (P2, C1, R1, S1), well volume 0 µL

Dispense 500 nL to (P2, C3, R1, S1), well volume 0 µL

Aspirate 1000 nL from (P3, C20, R1, S1), well volume 49 µL

Dispense 500 nL to (P2, C5, R1, S1), well volume 0 µL

Dispense 500 nL to (P2, C7, R1, S1), well volume 0 µL

Aspirate 1000 nL from (P3, C20, R1, S1), well volume 48 µL

Dispense 500 nL to (P2, C9, R1, S1), well volume 0 µL

Dispense 500 nL to (P2, C11, R1, S1), well volume 0 µL

Aspirate 1000 nL from (P3, C20, R1, S1), well volume 47 µL

Dispense 500 nL to (P2, C1, R2, S1), well volume 0 µL

Dispense 500 nL to (P2, C3, R2, S1), well volume 0 µL

Aspirate 1000 nL from (P3, C20, R1, S1), well volume 46 µL

Dispense 500 nL to (P2, C5, R2, S1), well volume 0 µL

Dispense 500 nL to (P2, C7, R2, S1), well volume 0 µL

Aspirate 1000 nL from (P3, C20, R1, S1), well volume 45 µL

Dispense 500 nL to (P2, C9, R2, S1), well volume 0 µL

Dispense 500 nL to (P2, C11, R2, S1), well volume 0 µL

Change pipettes

Aspirate 1000 nL from (P3, C24, R1, S1), well volume 42.4 µL

Dispense 500 nL to (P2, C2, R1, S1), well volume 0 µL

Dispense 500 nL to (P2, C4, R1, S1), well volume 0 µL

Aspirate 1000 nL from (P3, C24, R1, S1), well volume 41.4 µL

Dispense 500 nL to (P2, C6, R1, S1), well volume 0 µL

Dispense 500 nL to (P2, C8, R1, S1), well volume 0 µL

Aspirate 1000 nL from (P3, C24, R1, S1), well volume 40.4 µL

Dispense 500 nL to (P2, C10, R1, S1), well volume 0 µL

Dispense 500 nL to (P2, C12, R1, S1), well volume 0 µL

Aspirate 1000 nL from (P3, C24, R1, S1), well volume 39.4 µL

Dispense 500 nL to (P2, C2, R2, S1), well volume 0 µL

Dispense 500 nL to (P2, C4, R2, S1), well volume 0 µL

Aspirate 1000 nL from (P3, C24, R1, S1), well volume 38.4 µL

Dispense 500 nL to (P2, C6, R2, S1), well volume 0 µL

Dispense 500 nL to (P2, C8, R2, S1), well volume 0 µL

Aspirate 1000 nL from (P3, C24, R1, S1), well volume 37.4 µL

Dispense 500 nL to (P2, C10, R2, S1), well volume 0 µL

Dispense 500 nL to (P2, C12, R2, S1), well volume 0 µL

Change pipettes

Aspirate 500 nL from (P3, C19, R1, S1), well volume 50 µL, no over aspirate

Dispense 500 nL to (P2, C1, R1, S1), well volume 0 µL, mix cycles 3, mix volume 500nL, mix move 3mm

Change pipettes

Aspirate 500 nL from (P3, C19, R1, S1), well volume 49.5 µL, no over aspirate

Dispense 500 nL to (P2, C3, R1, S1), well volume 0 µL, mix cycles 3, mix volume 500nL, mix move 3mm

Change pipettes

Aspirate 500 nL from (P3, C19, R1, S1), well volume 49 µL, no over aspirate

Dispense 500 nL to (P2, C5, R1, S1), well volume 0 µL, mix cycles 3, mix volume 500nL, mix move 3mm

Change pipettes

Aspirate 500 nL from (P3, C19, R1, S1), well volume 48.5 µL, no over aspirate

Dispense 500 nL to (P2, C7, R1, S1), well volume 0 µL, mix cycles 3, mix volume 500nL, mix move 3mm

Change pipettes

Aspirate 500 nL from (P3, C19, R1, S1), well volume 48 µL, no over aspirate

Dispense 500 nL to (P2, C9, R1, S1), well volume 0 µL, mix cycles 3, mix volume 500nL, mix move 3mm

Change pipettes

Aspirate 500 nL from (P3, C19, R1, S1), well volume 47.5 µL, no over aspirate

Dispense 500 nL to (P2, C11, R1, S1), well volume 0 µL, mix cycles 3, mix volume 500nL, mix move 3mm

Change pipettes

Aspirate 500 nL from (P3, C19, R1, S1), well volume 47 µL, no over aspirate

Dispense 500 nL to (P2, C1, R2, S1), well volume 0 µL, mix cycles 3, mix volume 500nL, mix move 3mm

Change pipettes

Aspirate 500 nL from (P3, C19, R1, S1), well volume 46.5 µL, no over aspirate

Dispense 500 nL to (P2, C3, R2, S1), well volume 0 µL, mix cycles 3, mix volume 500nL, mix move 3mm

Change pipettes

Aspirate 500 nL from (P3, C19, R1, S1), well volume 46 µL, no over aspirate

Dispense 500 nL to (P2, C5, R2, S1), well volume 0 µL, mix cycles 3, mix volume 500nL, mix move 3mm

Change pipettes

Aspirate 500 nL from (P3, C19, R1, S1), well volume 45.5 µL, no over aspirate

Dispense 500 nL to (P2, C7, R2, S1), well volume 0 µL, mix cycles 3, mix volume 500nL, mix move 3mm

Change pipettes

Aspirate 500 nL from (P3, C19, R1, S1), well volume 45 µL, no over aspirate

Dispense 500 nL to (P2, C9, R2, S1), well volume 0 µL, mix cycles 3, mix volume 500nL, mix move 3mm

Change pipettes

Aspirate 500 nL from (P3, C19, R1, S1), well volume 44.5 µL, no over aspirate

Dispense 500 nL to (P2, C11, R2, S1), well volume 0 µL, mix cycles 3, mix volume 500nL, mix move 3mm

Change pipettes

Aspirate 500 nL from (P3, C19, R1, S1), well volume 44 µL, no over aspirate

Dispense 500 nL to (P2, C2, R1, S1), well volume 0 µL, mix cycles 3, mix volume 500nL, mix move 3mm

Change pipettes

Aspirate 500 nL from (P3, C19, R1, S1), well volume 43.5 µL, no over aspirate

Dispense 500 nL to (P2, C4, R1, S1), well volume 0 µL, mix cycles 3, mix volume 500nL, mix move 3mm

Change pipettes

Aspirate 500 nL from (P3, C19, R1, S1), well volume 43 µL, no over aspirate

Dispense 500 nL to (P2, C6, R1, S1), well volume 0 µL, mix cycles 3, mix volume 500nL, mix move 3mm

Change pipettes

Aspirate 500 nL from (P3, C19, R1, S1), well volume 42.5 µL, no over aspirate

Dispense 500 nL to (P2, C8, R1, S1), well volume 0 µL, mix cycles 3, mix volume 500nL, mix move 3mm

Change pipettes

Aspirate 500 nL from (P3, C19, R1, S1), well volume 42 µL, no over aspirate

Dispense 500 nL to (P2, C10, R1, S1), well volume 0 µL, mix cycles 3, mix volume 500nL, mix move 3mm

Change pipettes

Aspirate 500 nL from (P3, C19, R1, S1), well volume 41.5 µL, no over aspirate

Dispense 500 nL to (P2, C12, R1, S1), well volume 0 µL, mix cycles 3, mix volume 500nL, mix move 3mm

Change pipettes

Aspirate 500 nL from (P3, C19, R1, S1), well volume 41 µL, no over aspirate

Dispense 500 nL to (P2, C2, R2, S1), well volume 0 µL, mix cycles 3, mix volume 500nL, mix move 3mm

Change pipettes

Aspirate 500 nL from (P3, C19, R1, S1), well volume 40.5 µL, no over aspirate

Dispense 500 nL to (P2, C4, R2, S1), well volume 0 µL, mix cycles 3, mix volume 500nL, mix move 3mm

Change pipettes

Aspirate 500 nL from (P3, C19, R1, S1), well volume 40 µL, no over aspirate

Dispense 500 nL to (P2, C6, R2, S1), well volume 0 µL, mix cycles 3, mix volume 500nL, mix move 3mm

Change pipettes

Aspirate 500 nL from (P3, C19, R1, S1), well volume 39.5 µL, no over aspirate

Dispense 500 nL to (P2, C8, R2, S1), well volume 0 µL, mix cycles 3, mix volume 500nL, mix move 3mm

Change pipettes

Aspirate 500 nL from (P3, C19, R1, S1), well volume 39 µL, no over aspirate

Dispense 500 nL to (P2, C10, R2, S1), well volume 0 µL, mix cycles 3, mix volume 500nL, mix move 3mm

Change pipettes

Aspirate 500 nL from (P3, C19, R1, S1), well volume 38.5 µL, no over aspirate

Dispense 500 nL to (P2, C12, R2, S1), well volume 0 µL, mix cycles 3, mix volume 500nL, mix move 3mm

Change pipettes

Aspirate 1000 nL from (P3, C23, R1, S1), well volume 38 µL

Dispense 500 nL to (P4, C1, R1, S1), well volume 0 µL

Dispense 500 nL to (P4, C3, R1, S1), well volume 0 µL

Aspirate 1000 nL from (P3, C23, R1, S1), well volume 37 µL

Dispense 500 nL to (P4, C5, R1, S1), well volume 0 µL

Dispense 500 nL to (P4, C7, R1, S1), well volume 0 µL

Aspirate 1000 nL from (P3, C23, R1, S1), well volume 36 µL

Dispense 500 nL to (P4, C9, R1, S1), well volume 0 µL

Dispense 500 nL to (P4, C11, R1, S1), well volume 0 µL

Aspirate 1000 nL from (P3, C23, R1, S1), well volume 35 µL

Dispense 500 nL to (P4, C1, R1, S1), well volume 0.5 µL

Dispense 500 nL to (P4, C3, R1, S1), well volume 0.5 µL

Aspirate 1000 nL from (P3, C23, R1, S1), well volume 34 µL

Dispense 500 nL to (P4, C5, R1, S1), well volume 0.5 µL

Dispense 500 nL to (P4, C7, R1, S1), well volume 0.5 µL

Aspirate 1000 nL from (P3, C23, R1, S1), well volume 33 µL

Dispense 500 nL to (P4, C9, R1, S1), well volume 0.5 µL

Dispense 500 nL to (P4, C11, R1, S1), well volume 0.5 µL

Aspirate 1000 nL from (P3, C23, R1, S1), well volume 32 µL

Dispense 500 nL to (P4, C2, R1, S1), well volume 0 µL

Dispense 500 nL to (P4, C4, R1, S1), well volume 0 µL

Aspirate 1000 nL from (P3, C23, R1, S1), well volume 31 µL

Dispense 500 nL to (P4, C6, R1, S1), well volume 0 µL

Dispense 500 nL to (P4, C8, R1, S1), well volume 0 µL

Aspirate 1000 nL from (P3, C23, R1, S1), well volume 30 µL

Dispense 500 nL to (P4, C10, R1, S1), well volume 0 µL

Dispense 500 nL to (P4, C12, R1, S1), well volume 0 µL

Aspirate 1000 nL from (P3, C23, R1, S1), well volume 29 µL

Dispense 500 nL to (P4, C2, R1, S1), well volume 0.5 µL

Dispense 500 nL to (P4, C4, R1, S1), well volume 0.5 µL

Aspirate 1000 nL from (P3, C23, R1, S1), well volume 28 µL

Dispense 500 nL to (P4, C6, R1, S1), well volume 0.5 µL

Dispense 500 nL to (P4, C8, R1, S1), well volume 0.5 µL

Aspirate 1000 nL from (P3, C23, R1, S1), well volume 27 µL

Dispense 500 nL to (P4, C10, R1, S1), well volume 0.5 µL

Dispense 500 nL to (P4, C12, R1, S1), well volume 0.5 µL

Change pipettes

Aspirate 1000 nL from (P3, C22, R1, S1), well volume 42.6 µL

Dispense 125 nL to (P4, C1, R1, S1), well volume 1 µL

Dispense 125 nL to (P4, C3, R1, S1), well volume 1 µL

Dispense 125 nL to (P4, C1, R1, S1), well volume 1.125 µL

Dispense 125 nL to (P4, C3, R1, S1), well volume 1.125 µL

Dispense 125 nL to (P4, C2, R1, S1), well volume 1 µL

Dispense 125 nL to (P4, C4, R1, S1), well volume 1 µL

Dispense 125 nL to (P4, C2, R1, S1), well volume 1.125 µL

Dispense 125 nL to (P4, C4, R1, S1), well volume 1.125 µL

Aspirate 1200 nL from (P3, C22, R1, S1), well volume 41.6 µL

Dispense 300 nL to (P4, C5, R1, S1), well volume 1 µL

Dispense 300 nL to (P4, C7, R1, S1), well volume 1 µL

Dispense 300 nL to (P4, C5, R1, S1), well volume 1.3 µL

Dispense 300 nL to (P4, C7, R1, S1), well volume 1.3 µL

Aspirate 1200 nL from (P3, C22, R1, S1), well volume 40.4 µL

Dispense 300 nL to (P4, C6, R1, S1), well volume 1 µL

Dispense 300 nL to (P4, C8, R1, S1), well volume 1 µL

Dispense 300 nL to (P4, C6, R1, S1), well volume 1.3 µL

Dispense 300 nL to (P4, C8, R1, S1), well volume 1.3 µL

Aspirate 1000 nL from (P3, C22, R1, S1), well volume 39.2 µL

Dispense 500 nL to (P4, C9, R1, S1), well volume 1 µL

Dispense 500 nL to (P4, C11, R1, S1), well volume 1 µL

Aspirate 1000 nL from (P3, C22, R1, S1), well volume 38.2 µL

Dispense 500 nL to (P4, C9, R1, S1), well volume 1.5 µL

Dispense 500 nL to (P4, C11, R1, S1), well volume 1.5 µL

Aspirate 1000 nL from (P3, C22, R1, S1), well volume 37.2 µL

Dispense 500 nL to (P4, C10, R1, S1), well volume 1 µL

Dispense 500 nL to (P4, C12, R1, S1), well volume 1 µL

Aspirate 1000 nL from (P3, C22, R1, S1), well volume 36.2 µL

Dispense 500 nL to (P4, C10, R1, S1), well volume 1.5 µL

Dispense 500 nL to (P4, C12, R1, S1), well volume 1.5 µL

Change pipettes

Aspirate 1125 nL from (P3, C24, R1, S1), well volume 36.4 µL

Dispense 375 nL to (P4, C1, R1, S1), well volume 1.25 µL

Dispense 375 nL to (P4, C3, R1, S1), well volume 1.25 µL

Dispense 375 nL to (P4, C1, R1, S1), well volume 1.625 µL

Aspirate 1125 nL from (P3, C24, R1, S1), well volume 35.275 µL

Dispense 375 nL to (P4, C3, R1, S1), well volume 1.625 µL

Dispense 375 nL to (P4, C2, R1, S1), well volume 1.25 µL

Dispense 375 nL to (P4, C4, R1, S1), well volume 1.25 µL

Aspirate 1150 nL from (P3, C24, R1, S1), well volume 34.15 µL

Dispense 375 nL to (P4, C2, R1, S1), well volume 1.625 µL

Dispense 375 nL to (P4, C4, R1, S1), well volume 1.625 µL

Dispense 200 nL to (P4, C5, R1, S1), well volume 1.6 µL

Dispense 200 nL to (P4, C7, R1, S1), well volume 1.6 µL

Aspirate 1200 nL from (P3, C24, R1, S1), well volume 33 µL

Dispense 200 nL to (P4, C5, R1, S1), well volume 1.8 µL

Dispense 200 nL to (P4, C7, R1, S1), well volume 1.8 µL

Dispense 200 nL to (P4, C6, R1, S1), well volume 1.6 µL

Dispense 200 nL to (P4, C8, R1, S1), well volume 1.6 µL

Dispense 200 nL to (P4, C6, R1, S1), well volume 1.8 µL

Dispense 200 nL to (P4, C8, R1, S1), well volume 1.8 µL

Change pipettes

Aspirate 1000 nL from (P3, C21, R1, S1), well volume 41 µL

Dispense 250 nL to (P4, C1, R1, S1), well volume 2 µL

Dispense 250 nL to (P4, C1, R1, S1), well volume 2.25 µL

Dispense 250 nL to (P4, C2, R1, S1), well volume 2 µL

Dispense 250 nL to (P4, C2, R1, S1), well volume 2.25 µL

Aspirate 1000 nL from (P3, C21, R1, S1), well volume 40 µL

Dispense 500 nL to (P4, C3, R1, S1), well volume 2 µL

Dispense 500 nL to (P4, C3, R1, S1), well volume 2.5 µL

Aspirate 1000 nL from (P3, C21, R1, S1), well volume 39 µL

Dispense 500 nL to (P4, C4, R1, S1), well volume 2 µL

Dispense 500 nL to (P4, C4, R1, S1), well volume 2.5 µL

Aspirate 1000 nL from (P3, C21, R1, S1), well volume 38 µL

Dispense 250 nL to (P4, C5, R1, S1), well volume 2 µL

Dispense 250 nL to (P4, C5, R1, S1), well volume 2.25 µL

Dispense 250 nL to (P4, C6, R1, S1), well volume 2 µL

Dispense 250 nL to (P4, C6, R1, S1), well volume 2.25 µL

Aspirate 1000 nL from (P3, C21, R1, S1), well volume 37 µL

Dispense 500 nL to (P4, C7, R1, S1), well volume 2 µL

Dispense 500 nL to (P4, C7, R1, S1), well volume 2.5 µL

Aspirate 1000 nL from (P3, C21, R1, S1), well volume 36 µL

Dispense 500 nL to (P4, C8, R1, S1), well volume 2 µL

Dispense 500 nL to (P4, C8, R1, S1), well volume 2.5 µL

Aspirate 1000 nL from (P3, C21, R1, S1), well volume 35 µL

Dispense 250 nL to (P4, C9, R1, S1), well volume 2 µL

Dispense 250 nL to (P4, C9, R1, S1), well volume 2.25 µL

Dispense 250 nL to (P4, C10, R1, S1), well volume 2 µL

Dispense 250 nL to (P4, C10, R1, S1), well volume 2.25 µL

Aspirate 1000 nL from (P3, C21, R1, S1), well volume 34 µL

Dispense 500 nL to (P4, C11, R1, S1), well volume 2 µL

Dispense 500 nL to (P4, C11, R1, S1), well volume 2.5 µL

Aspirate 1000 nL from (P3, C21, R1, S1), well volume 33 µL

Dispense 500 nL to (P4, C12, R1, S1), well volume 2 µL

Dispense 500 nL to (P4, C12, R1, S1), well volume 2.5 µL

Change pipettes

Aspirate 1000 nL from (P3, C24, R1, S1), well volume 31.8 µL

Dispense 250 nL to (P4, C1, R1, S1), well volume 2.5 µL

Dispense 250 nL to (P4, C1, R1, S1), well volume 2.75 µL

Dispense 250 nL to (P4, C2, R1, S1), well volume 2.5 µL

Dispense 250 nL to (P4, C2, R1, S1), well volume 2.75 µL

Aspirate 1000 nL from (P3, C24, R1, S1), well volume 30.8 µL

Dispense 250 nL to (P4, C5, R1, S1), well volume 2.5 µL

Dispense 250 nL to (P4, C5, R1, S1), well volume 2.75 µL

Dispense 250 nL to (P4, C6, R1, S1), well volume 2.5 µL

Dispense 250 nL to (P4, C6, R1, S1), well volume 2.75 µL

Aspirate 1000 nL from (P3, C24, R1, S1), well volume 29.8 µL

Dispense 250 nL to (P4, C9, R1, S1), well volume 2.5 µL

Dispense 250 nL to (P4, C9, R1, S1), well volume 2.75 µL

Dispense 250 nL to (P4, C10, R1, S1), well volume 2.5 µL

Dispense 250 nL to (P4, C10, R1, S1), well volume 2.75 µL

Change pipettes

Aspirate 1000 nL from (P3, C20, R1, S1), well volume 44 µL

Dispense 500 nL to (P4, C1, R1, S1), well volume 3 µL

Dispense 500 nL to (P4, C3, R1, S1), well volume 3 µL

Aspirate 1000 nL from (P3, C20, R1, S1), well volume 43 µL

Dispense 500 nL to (P4, C5, R1, S1), well volume 3 µL

Dispense 500 nL to (P4, C7, R1, S1), well volume 3 µL

Aspirate 1000 nL from (P3, C20, R1, S1), well volume 42 µL

Dispense 500 nL to (P4, C9, R1, S1), well volume 3 µL

Dispense 500 nL to (P4, C11, R1, S1), well volume 3 µL

Aspirate 1000 nL from (P3, C20, R1, S1), well volume 41 µL

Dispense 500 nL to (P4, C1, R1, S1), well volume 3.5 µL

Dispense 500 nL to (P4, C3, R1, S1), well volume 3.5 µL

Aspirate 1000 nL from (P3, C20, R1, S1), well volume 40 µL

Dispense 500 nL to (P4, C5, R1, S1), well volume 3.5 µL

Dispense 500 nL to (P4, C7, R1, S1), well volume 3.5 µL

Aspirate 1000 nL from (P3, C20, R1, S1), well volume 39 µL

Dispense 500 nL to (P4, C9, R1, S1), well volume 3.5 µL

Dispense 500 nL to (P4, C11, R1, S1), well volume 3.5 µL

Change pipettes

Aspirate 1000 nL from (P3, C24, R1, S1), well volume 28.8 µL

Dispense 500 nL to (P4, C2, R1, S1), well volume 3 µL

Dispense 500 nL to (P4, C4, R1, S1), well volume 3 µL

Aspirate 1000 nL from (P3, C24, R1, S1), well volume 27.8 µL

Dispense 500 nL to (P4, C6, R1, S1), well volume 3 µL

Dispense 500 nL to (P4, C8, R1, S1), well volume 3 µL

Aspirate 1000 nL from (P3, C24, R1, S1), well volume 26.8 µL

Dispense 500 nL to (P4, C10, R1, S1), well volume 3 µL

Dispense 500 nL to (P4, C12, R1, S1), well volume 3 µL

Aspirate 1000 nL from (P3, C24, R1, S1), well volume 25.8 µL

Dispense 500 nL to (P4, C2, R1, S1), well volume 3.5 µL

Dispense 500 nL to (P4, C4, R1, S1), well volume 3.5 µL

Aspirate 1000 nL from (P3, C24, R1, S1), well volume 24.8 µL

Dispense 500 nL to (P4, C6, R1, S1), well volume 3.5 µL

Dispense 500 nL to (P4, C8, R1, S1), well volume 3.5 µL

Aspirate 1000 nL from (P3, C24, R1, S1), well volume 23.8 µL

Dispense 500 nL to (P4, C10, R1, S1), well volume 3.5 µL

Dispense 500 nL to (P4, C12, R1, S1), well volume 3.5 µL

Change pipettes

Aspirate 500 nL from (P3, C19, R1, S1), well volume 38 µL, no over aspirate

Dispense 500 nL to (P4, C1, R1, S1), well volume 4 µL, mix cycles 3, mix volume 500nL, mix move 3mm

Change pipettes

Aspirate 500 nL from (P3, C19, R1, S1), well volume 37.5 µL, no over aspirate

Dispense 500 nL to (P4, C3, R1, S1), well volume 4 µL, mix cycles 3, mix volume 500nL, mix move 3mm

Change pipettes

Aspirate 500 nL from (P3, C19, R1, S1), well volume 37 µL, no over aspirate

Dispense 500 nL to (P4, C5, R1, S1), well volume 4 µL, mix cycles 3, mix volume 500nL, mix move 3mm

Change pipettes

Aspirate 500 nL from (P3, C19, R1, S1), well volume 36.5 µL, no over aspirate

Dispense 500 nL to (P4, C7, R1, S1), well volume 4 µL, mix cycles 3, mix volume 500nL, mix move 3mm

Change pipettes

Aspirate 500 nL from (P3, C19, R1, S1), well volume 36 µL, no over aspirate

Dispense 500 nL to (P4, C9, R1, S1), well volume 4 µL, mix cycles 3, mix volume 500nL, mix move 3mm

Change pipettes

Aspirate 500 nL from (P3, C19, R1, S1), well volume 35.5 µL, no over aspirate

Dispense 500 nL to (P4, C11, R1, S1), well volume 4 µL, mix cycles 3, mix volume 500nL, mix move 3mm

Change pipettes

Aspirate 500 nL from (P3, C19, R1, S1), well volume 35 µL, no over aspirate

Dispense 500 nL to (P4, C1, R1, S1), well volume 4.5 µL, mix cycles 3, mix volume 500nL, mix move 3mm

Change pipettes

Aspirate 500 nL from (P3, C19, R1, S1), well volume 34.5 µL, no over aspirate

Dispense 500 nL to (P4, C3, R1, S1), well volume 4.5 µL, mix cycles 3, mix volume 500nL, mix move 3mm

Change pipettes

Aspirate 500 nL from (P3, C19, R1, S1), well volume 34 µL, no over aspirate

Dispense 500 nL to (P4, C5, R1, S1), well volume 4.5 µL, mix cycles 3, mix volume 500nL, mix move 3mm

Change pipettes

Aspirate 500 nL from (P3, C19, R1, S1), well volume 33.5 µL, no over aspirate

Dispense 500 nL to (P4, C7, R1, S1), well volume 4.5 µL, mix cycles 3, mix volume 500nL, mix move 3mm

Change pipettes

Aspirate 500 nL from (P3, C19, R1, S1), well volume 33 µL, no over aspirate

Dispense 500 nL to (P4, C9, R1, S1), well volume 4.5 µL, mix cycles 3, mix volume 500nL, mix move 3mm

Change pipettes

Aspirate 500 nL from (P3, C19, R1, S1), well volume 32.5 µL, no over aspirate

Dispense 500 nL to (P4, C11, R1, S1), well volume 4.5 µL, mix cycles 3, mix volume 500nL, mix move 3mm

Change pipettes

Aspirate 500 nL from (P3, C19, R1, S1), well volume 32 µL, no over aspirate

Dispense 500 nL to (P4, C2, R1, S1), well volume 4 µL, mix cycles 3, mix volume 500nL, mix move 3mm

Change pipettes

Aspirate 500 nL from (P3, C19, R1, S1), well volume 31.5 µL, no over aspirate

Dispense 500 nL to (P4, C4, R1, S1), well volume 4 µL, mix cycles 3, mix volume 500nL, mix move 3mm

Change pipettes

Aspirate 500 nL from (P3, C19, R1, S1), well volume 31 µL, no over aspirate

Dispense 500 nL to (P4, C6, R1, S1), well volume 4 µL, mix cycles 3, mix volume 500nL, mix move 3mm

Change pipettes

Aspirate 500 nL from (P3, C19, R1, S1), well volume 30.5 µL, no over aspirate

Dispense 500 nL to (P4, C8, R1, S1), well volume 4 µL, mix cycles 3, mix volume 500nL, mix move 3mm

Change pipettes

Aspirate 500 nL from (P3, C19, R1, S1), well volume 30 µL, no over aspirate

Dispense 500 nL to (P4, C10, R1, S1), well volume 4 µL, mix cycles 3, mix volume 500nL, mix move 3mm

Change pipettes

Aspirate 500 nL from (P3, C19, R1, S1), well volume 29.5 µL, no over aspirate

Dispense 500 nL to (P4, C12, R1, S1), well volume 4 µL, mix cycles 3, mix volume 500nL, mix move 3mm

Change pipettes

Aspirate 500 nL from (P3, C19, R1, S1), well volume 29 µL, no over aspirate

Dispense 500 nL to (P4, C2, R1, S1), well volume 4.5 µL, mix cycles 3, mix volume 500nL, mix move 3mm

Change pipettes

Aspirate 500 nL from (P3, C19, R1, S1), well volume 28.5 µL, no over aspirate

Dispense 500 nL to (P4, C4, R1, S1), well volume 4.5 µL, mix cycles 3, mix volume 500nL, mix move 3mm

Change pipettes

Aspirate 500 nL from (P3, C19, R1, S1), well volume 28 µL, no over aspirate

Dispense 500 nL to (P4, C6, R1, S1), well volume 4.5 µL, mix cycles 3, mix volume 500nL, mix move 3mm

Change pipettes

Aspirate 500 nL from (P3, C19, R1, S1), well volume 27.5 µL, no over aspirate

Dispense 500 nL to (P4, C8, R1, S1), well volume 4.5 µL, mix cycles 3, mix volume 500nL, mix move 3mm

Change pipettes

Aspirate 500 nL from (P3, C19, R1, S1), well volume 27 µL, no over aspirate

Dispense 500 nL to (P4, C10, R1, S1), well volume 4.5 µL, mix cycles 3, mix volume 500nL, mix move 3mm

Change pipettes

Aspirate 500 nL from (P3, C19, R1, S1), well volume 26.5 µL, no over aspirate

Dispense 500 nL to (P4, C12, R1, S1), well volume 4.5 µL, mix cycles 3, mix volume 500nL, mix move 3mm

Change pipettes

Analysis plate dosing

5 position deck

Position:

1: [no plate]

2: Greiner 384 (v bottom); Plate Id: Analysis Plate 1

3: Corning 1536 COC white; Plate Id: Reaction Plate

4: Greiner 384 (v bottom); Plate Id: Analysis Plate 1

5: [no plate]

Home tape

Aspirate 1000 nL from (P3, C1, R1, S1), well volume 0 µL, no over aspirate

Dispense 1000 nL to (P2, C1, R1, S1), well volume 95 µL, mix cycles 3, mix volume 1000nL, mix move 5mm, manual height: 5 mm

Aspirate 1000 nL from (P3, C1, R1, S1), well volume 0 µL, no over aspirate

Dispense 1000 nL to (P2, C1, R1, S1), well volume 96 µL, mix cycles 3, mix volume 1000nL, mix move 5mm, manual height: 5 mm

Change pipettes

Aspirate 1000 nL from (P3, C1, R2, S1), well volume 0 µL, no over aspirate

Dispense 1000 nL to (P2, C2, R1, S1), well volume 95 µL, mix cycles 3, mix volume 1000nL, mix move 5mm, manual height: 5 mm

Aspirate 1000 nL from (P3, C1, R2, S1), well volume 0 µL, no over aspirate

Dispense 1000 nL to (P2, C2, R1, S1), well volume 96 µL, mix cycles 3, mix volume 1000nL, mix move 5mm, manual height: 5 mm

Change pipettes

Aspirate 1000 nL from (P3, C2, R1, S1), well volume 0 µL, no over aspirate

Dispense 1000 nL to (P2, C3, R1, S1), well volume 95 µL, mix cycles 3, mix volume 1000nL, mix move 5mm, manual height: 5 mm

Aspirate 1000 nL from (P3, C2, R1, S1), well volume 0 µL, no over aspirate

Dispense 1000 nL to (P2, C3, R1, S1), well volume 96 µL, mix cycles 3, mix volume 1000nL, mix move 5mm, manual height: 5 mm

Change pipettes

Aspirate 1000 nL from (P3, C2, R2, S1), well volume 0 µL, no over aspirate

Dispense 1000 nL to (P2, C4, R1, S1), well volume 95 µL, mix cycles 3, mix volume 1000nL, mix move 5mm, manual height: 5 mm

Aspirate 1000 nL from (P3, C2, R2, S1), well volume 0 µL, no over aspirate

Dispense 1000 nL to (P2, C4, R1, S1), well volume 96 µL, mix cycles 3, mix volume 1000nL, mix move 5mm, manual height: 5 mm

Change pipettes

Aspirate 1000 nL from (P3, C3, R1, S1), well volume 0 µL, no over aspirate

Dispense 1000 nL to (P2, C5, R1, S1), well volume 95 µL, mix cycles 3, mix volume 1000nL, mix move 5mm, manual height: 5 mm

Aspirate 1000 nL from (P3, C3, R1, S1), well volume 0 µL, no over aspirate

Dispense 1000 nL to (P2, C5, R1, S1), well volume 96 µL, mix cycles 3, mix volume 1000nL, mix move 5mm, manual height: 5 mm

Change pipettes

Aspirate 1000 nL from (P3, C3, R2, S1), well volume 0 µL, no over aspirate

Dispense 1000 nL to (P2, C6, R1, S1), well volume 95 µL, mix cycles 3, mix volume 1000nL, mix move 5mm, manual height: 5 mm

Aspirate 1000 nL from (P3, C2, R2, S1), well volume 0 µL, no over aspirate

Dispense 1000 nL to (P2, C6, R1, S1), well volume 96 µL, mix cycles 3, mix volume 1000nL, mix move 5mm, manual height: 5 mm

Change pipettes

Aspirate 1000 nL from (P3, C4, R1, S1), well volume 0 µL, no over aspirate

Dispense 1000 nL to (P2, C7, R1, S1), well volume 95 µL, mix cycles 3, mix volume 1000nL, mix move 5mm, manual height: 5 mm

Aspirate 1000 nL from (P3, C4, R1, S1), well volume 0 µL, no over aspirate

Dispense 1000 nL to (P2, C7, R1, S1), well volume 96 µL, mix cycles 3, mix volume 1000nL, mix move 5mm, manual height: 5 mm

Change pipettes

Aspirate 1000 nL from (P3, C4, R2, S1), well volume 0 µL, no over aspirate

Dispense 1000 nL to (P2, C8, R1, S1), well volume 95 µL, mix cycles 3, mix volume 1000nL, mix move 5mm, manual height: 5 mm

Aspirate 1000 nL from (P3, C4, R2, S1), well volume 0 µL, no over aspirate

Dispense 1000 nL to (P2, C8, R1, S1), well volume 96 µL, mix cycles 3, mix volume 1000nL, mix move 5mm, manual height: 5 mm

Change pipettes

Aspirate 1000 nL from (P3, C5, R1, S1), well volume 0 µL, no over aspirate

Dispense 1000 nL to (P2, C9, R1, S1), well volume 95 µL, mix cycles 3, mix volume 1000nL, mix move 5mm, manual height: 5 mm

Aspirate 1000 nL from (P3, C5, R1, S1), well volume 0 µL, no over aspirate

Dispense 1000 nL to (P2, C9, R1, S1), well volume 96 µL, mix cycles 3, mix volume 1000nL, mix move 5mm, manual height: 5 mm

Change pipettes

Aspirate 1000 nL from (P3, C5, R2, S1), well volume 0 µL, no over aspirate

Dispense 1000 nL to (P2, C10, R1, S1), well volume 95 µL, mix cycles 3, mix volume 1000nL, mix move 5mm, manual height: 5 mm

Aspirate 1000 nL from (P3, C5, R2, S1), well volume 0 µL, no over aspirate

Dispense 1000 nL to (P2, C10, R1, S1), well volume 96 µL, mix cycles 3, mix volume 1000nL, mix move 5mm, manual height: 5 mm

Change pipettes

Aspirate 1000 nL from (P3, C6, R1, S1), well volume 0 µL, no over aspirate

Dispense 1000 nL to (P2, C11, R1, S1), well volume 95 µL, mix cycles 3, mix volume 1000nL, mix move 5mm, manual height: 5 mm

Aspirate 1000 nL from (P3, C6, R1, S1), well volume 0 µL, no over aspirate

Dispense 1000 nL to (P2, C11, R1, S1), well volume 96 µL, mix cycles 3, mix volume 1000nL, mix move 5mm, manual height: 5 mm

Change pipettes

Aspirate 1000 nL from (P3, C6, R2, S1), well volume 0 µL, no over aspirate

Dispense 1000 nL to (P2, C12, R1, S1), well volume 95 µL, mix cycles 3, mix volume 1000nL, mix move 5mm, manual height: 5 mm

Aspirate 1000 nL from (P3, C6, R2, S1), well volume 0 µL, no over aspirate

Dispense 1000 nL to (P2, C12, R1, S1), well volume 96 µL, mix cycles 3, mix volume 1000nL, mix move 5mm, manual height: 5 mm

Change pipettes

Aspirate 1000 nL from (P3, C7, R1, S1), well volume 0 µL, no over aspirate

Dispense 1000 nL to (P2, C13, R1, S1), well volume 95 µL, mix cycles 3, mix volume 1000nL, mix move 5mm, manual height: 5 mm

Aspirate 1000 nL from (P3, C7, R1, S1), well volume 0 µL, no over aspirate

Dispense 1000 nL to (P2, C13, R1, S1), well volume 96 µL, mix cycles 3, mix volume 1000nL, mix move 5mm, manual height: 5 mm

Change pipettes

Aspirate 1000 nL from (P3, C7, R2, S1), well volume 0 µL, no over aspirate

Dispense 1000 nL to (P2, C14, R1, S1), well volume 95 µL, mix cycles 3, mix volume 1000nL, mix move 5mm, manual height: 5 mm

Aspirate 1000 nL from (P3, C7, R2, S1), well volume 0 µL, no over aspirate

Dispense 1000 nL to (P2, C14, R1, S1), well volume 96 µL, mix cycles 3, mix volume 1000nL, mix move 5mm, manual height: 5 mm

Change pipettes

Aspirate 1000 nL from (P3, C8, R1, S1), well volume 0 µL, no over aspirate

Dispense 1000 nL to (P2, C15, R1, S1), well volume 95 µL, mix cycles 3, mix volume 1000nL, mix move 5mm, manual height: 5 mm

Aspirate 1000 nL from (P3, C8, R1, S1), well volume 0 µL, no over aspirate

Dispense 1000 nL to (P2, C15, R1, S1), well volume 96 µL, mix cycles 3, mix volume 1000nL, mix move 5mm, manual height: 5 mm

Change pipettes

Aspirate 1000 nL from (P3, C8, R2, S1), well volume 0 µL, no over aspirate

Dispense 1000 nL to (P2, C16, R1, S1), well volume 95 µL, mix cycles 3, mix volume 1000nL, mix move 5mm, manual height: 5 mm

Aspirate 1000 nL from (P3, C8, R2, S1), well volume 0 µL, no over aspirate

Dispense 1000 nL to (P2, C16, R1, S1), well volume 96 µL, mix cycles 3, mix volume 1000nL, mix move 5mm, manual height: 5 mm

Change pipettes

Aspirate 1000 nL from (P3, C9, R1, S1), well volume 0 µL, no over aspirate

Dispense 1000 nL to (P2, C17, R1, S1), well volume 95 µL, mix cycles 3, mix volume 1000nL, mix move 5mm, manual height: 5 mm

Aspirate 1000 nL from (P3, C9, R1, S1), well volume 0 µL, no over aspirate

Dispense 1000 nL to (P2, C17, R1, S1), well volume 96 µL, mix cycles 3, mix volume 1000nL, mix move 5mm, manual height: 5 mm

Change pipettes

Aspirate 1000 nL from (P3, C9, R2, S1), well volume 0 µL, no over aspirate

Dispense 1000 nL to (P2, C18, R1, S1), well volume 95 µL, mix cycles 3, mix volume 1000nL, mix move 5mm, manual height: 5 mm

Aspirate 1000 nL from (P3, C9, R2, S1), well volume 0 µL, no over aspirate

Dispense 1000 nL to (P2, C18, R1, S1), well volume 96 µL, mix cycles 3, mix volume 1000nL, mix move 5mm, manual height: 5 mm

Change pipettes

Aspirate 1000 nL from (P3, C10, R1, S1), well volume 0 µL, no over aspirate

Dispense 1000 nL to (P2, C19, R1, S1), well volume 95 µL, mix cycles 3, mix volume 1000nL, mix move 5mm, manual height: 5 mm

Aspirate 1000 nL from (P3, C10, R1, S1), well volume 0 µL, no over aspirate

Dispense 1000 nL to (P2, C19, R1, S1), well volume 96 µL, mix cycles 3, mix volume 1000nL, mix move 5mm, manual height: 5 mm

Change pipettes

Aspirate 1000 nL from (P3, C10, R2, S1), well volume 0 µL, no over aspirate

Dispense 1000 nL to (P2, C20, R1, S1), well volume 95 µL, mix cycles 3, mix volume 1000nL, mix move 5mm, manual height: 5 mm

Aspirate 1000 nL from (P3, C10, R2, S1), well volume 0 µL, no over aspirate

Dispense 1000 nL to (P2, C20, R1, S1), well volume 96 µL, mix cycles 3, mix volume 1000nL, mix move 5mm, manual height: 5 mm

Change pipettes

Aspirate 1000 nL from (P3, C11, R1, S1), well volume 0 µL, no over aspirate

Dispense 1000 nL to (P2, C21, R1, S1), well volume 95 µL, mix cycles 3, mix volume 1000nL, mix move 5mm, manual height: 5 mm

Aspirate 1000 nL from (P3, C11, R1, S1), well volume 0 µL, no over aspirate

Dispense 1000 nL to (P2, C21, R1, S1), well volume 96 µL, mix cycles 3, mix volume 1000nL, mix move 5mm, manual height: 5 mm

Change pipettes

Aspirate 1000 nL from (P3, C11, R2, S1), well volume 0 µL, no over aspirate

Dispense 1000 nL to (P2, C22, R1, S1), well volume 95 µL, mix cycles 3, mix volume 1000nL, mix move 5mm, manual height: 5 mm

Aspirate 1000 nL from (P3, C11, R2, S1), well volume 0 µL, no over aspirate

Dispense 1000 nL to (P2, C22, R1, S1), well volume 96 µL, mix cycles 3, mix volume 1000nL, mix move 5mm, manual height: 5 mm

Change pipettes

Aspirate 1000 nL from (P3, C12, R1, S1), well volume 0 µL, no over aspirate

Dispense 1000 nL to (P2, C23, R1, S1), well volume 95 µL, mix cycles 3, mix volume 1000nL, mix move 5mm, manual height: 5 mm

Aspirate 1000 nL from (P3, C12, R1, S1), well volume 0 µL, no over aspirate

Dispense 1000 nL to (P2, C23, R1, S1), well volume 96 µL, mix cycles 3, mix volume 1000nL, mix move 5mm, manual height: 5 mm

Change pipettes

Aspirate 1000 nL from (P3, C12, R2, S1), well volume 0 µL, no over aspirate

Dispense 1000 nL to (P2, C24, R1, S1), well volume 95 µL, mix cycles 3, mix volume 1000nL, mix move 5mm, manual height: 5 mm

Aspirate 1000 nL from (P3, C12, R2, S1), well volume 0 µL, no over aspirate

Dispense 1000 nL to (P2, C24, R1, S1), well volume 96 µL, mix cycles 3, mix volume 1000nL, mix move 5mm, manual height: 5 mm

Change pipettes

Aspirate 1000 nL from (P3, C1, R1, S1), well volume 0 µL, no over aspirate

Dispense 1000 nL to (P4, C1, R1, S1), well volume 0 µL, mix cycles 3, mix volume 1000nL, mix move 5mm, manual height: 5 mm

Aspirate 1000 nL from (P3, C1, R1, S1), well volume 0 µL, no over aspirate

Dispense 1000 nL to (P4, C1, R1, S1), well volume 0 µL, mix cycles 3, mix volume 1000nL, mix move 5mm, manual height: 5 mm

Change pipettes

Aspirate 1000 nL from (P3, C1, R2, S1), well volume 0 µL, no over aspirate

Dispense 1000 nL to (P4, C2, R1, S1), well volume 0 µL, mix cycles 3, mix volume 1000nL, mix move 5mm, manual height: 5 mm

Aspirate 1000 nL from (P3, C1, R2, S1), well volume 0 µL, no over aspirate

Dispense 1000 nL to (P4, C2, R1, S1), well volume 0 µL, mix cycles 3, mix volume 1000nL, mix move 5mm, manual height: 5 mm

Change pipettes

Aspirate 1000 nL from (P3, C2, R1, S1), well volume 0 µL, no over aspirate

Dispense 1000 nL to (P4, C3, R1, S1), well volume 0 µL, mix cycles 3, mix volume 1000nL, mix move 5mm, manual height: 5 mm

Aspirate 1000 nL from (P3, C2, R1, S1), well volume 0 µL, no over aspirate

Dispense 1000 nL to (P4, C3, R1, S1), well volume 0 µL, mix cycles 3, mix volume 1000nL, mix move 5mm, manual height: 5 mm

Change pipettes

Aspirate 1000 nL from (P3, C2, R2, S1), well volume 0 µL, no over aspirate

Dispense 1000 nL to (P4, C4, R1, S1), well volume 0 µL, mix cycles 3, mix volume 1000nL, mix move 5mm, manual height: 5 mm

Aspirate 1000 nL from (P3, C2, R2, S1), well volume 0 µL, no over aspirate

Dispense 1000 nL to (P4, C4, R1, S1), well volume 0 µL, mix cycles 3, mix volume 1000nL, mix move 5mm, manual height: 5 mm

Change pipettes

Aspirate 1000 nL from (P3, C3, R1, S1), well volume 0 µL, no over aspirate

Dispense 1000 nL to (P4, C5, R1, S1), well volume 0 µL, mix cycles 3, mix volume 1000nL, mix move 5mm, manual height: 5 mm

Aspirate 1000 nL from (P3, C3, R1, S1), well volume 0 µL, no over aspirate

Dispense 1000 nL to (P4, C5, R1, S1), well volume 0 µL, mix cycles 3, mix volume 1000nL, mix move 5mm, manual height: 5 mm

Change pipettes

Aspirate 1000 nL from (P3, C3, R2, S1), well volume 0 µL, no over aspirate

Dispense 1000 nL to (P4, C6, R1, S1), well volume 0 µL, mix cycles 3, mix volume 1000nL, mix move 5mm, manual height: 5 mm

Aspirate 1000 nL from (P3, C3, R2, S1), well volume 0 µL, no over aspirate

Dispense 1000 nL to (P4, C6, R1, S1), well volume 0 µL, mix cycles 3, mix volume 1000nL, mix move 5mm, manual height: 5 mm

Change pipettes

Aspirate 1000 nL from (P3, C4, R1, S1), well volume 0 µL, no over aspirate

Dispense 1000 nL to (P4, C7, R1, S1), well volume 0 µL, mix cycles 3, mix volume 1000nL, mix move 5mm, manual height: 5 mm

Aspirate 1000 nL from (P3, C4, R1, S1), well volume 0 µL, no over aspirate

Dispense 1000 nL to (P4, C7, R1, S1), well volume 0 µL, mix cycles 3, mix volume 1000nL, mix move 5mm, manual height: 5 mm

Change pipettes

Aspirate 1000 nL from (P3, C4, R2, S1), well volume 0 µL, no over aspirate

Dispense 1000 nL to (P4, C8, R1, S1), well volume 0 µL, mix cycles 3, mix volume 1000nL, mix move 5mm, manual height: 5 mm

Aspirate 1000 nL from (P3, C4, R2, S1), well volume 0 µL, no over aspirate

Dispense 1000 nL to (P4, C8, R1, S1), well volume 0 µL, mix cycles 3, mix volume 1000nL, mix move 5mm, manual height: 5 mm

Change pipettes

Aspirate 1000 nL from (P3, C5, R1, S1), well volume 0 µL, no over aspirate

Dispense 1000 nL to (P4, C9, R1, S1), well volume 0 µL, mix cycles 3, mix volume 1000nL, mix move 5mm, manual height: 5 mm

Aspirate 1000 nL from (P3, C5, R1, S1), well volume 0 µL, no over aspirate

Dispense 1000 nL to (P4, C9, R1, S1), well volume 0 µL, mix cycles 3, mix volume 1000nL, mix move 5mm, manual height: 5 mm

Change pipettes

Aspirate 1000 nL from (P3, C5, R2, S1), well volume 0 µL, no over aspirate

Dispense 1000 nL to (P4, C10, R1, S1), well volume 0 µL, mix cycles 3, mix volume 1000nL, mix move 5mm, manual height: 5 mm

Aspirate 1000 nL from (P3, C5, R2, S1), well volume 0 µL, no over aspirate

Dispense 1000 nL to (P4, C10, R1, S1), well volume 0 µL, mix cycles 3, mix volume 1000nL, mix move 5mm, manual height: 5 mm

Change pipettes

Aspirate 1000 nL from (P3, C6, R1, S1), well volume 0 µL, no over aspirate

Dispense 1000 nL to (P4, C11, R1, S1), well volume 0 µL, mix cycles 3, mix volume 1000nL, mix move 5mm, manual height: 5 mm

Aspirate 1000 nL from (P3, C6, R1, S1), well volume 0 µL, no over aspirate

Dispense 1000 nL to (P4, C11, R1, S1), well volume 0 µL, mix cycles 3, mix volume 1000nL, mix move 5mm, manual height: 5 mm

Change pipettes

Aspirate 1000 nL from (P3, C6, R2, S1), well volume 0 µL, no over aspirate

Dispense 1000 nL to (P4, C12, R1, S1), well volume 0 µL, mix cycles 3, mix volume 1000nL, mix move 5mm, manual height: 5 mm

Aspirate 1000 nL from (P3, C6, R2, S1), well volume 0 µL, no over aspirate

Dispense 1000 nL to (P4, C12, R1, S1), well volume 0 µL, mix cycles 3, mix volume 1000nL, mix move 5mm, manual height: 5 mm

Change pipettes

Aspirate 1000 nL from (P3, C7, R1, S1), well volume 0 µL, no over aspirate

Dispense 1000 nL to (P4, C13, R1, S1), well volume 0 µL, mix cycles 3, mix volume 1000nL, mix move 5mm, manual height: 5 mm

Aspirate 1000 nL from (P3, C7, R1, S1), well volume 0 µL, no over aspirate

Dispense 1000 nL to (P4, C13, R1, S1), well volume 0 µL, mix cycles 3, mix volume 1000nL, mix move 5mm, manual height: 5 mm

Change pipettes

Aspirate 1000 nL from (P3, C7, R2, S1), well volume 0 µL, no over aspirate

Dispense 1000 nL to (P4, C14, R1, S1), well volume 0 µL, mix cycles 3, mix volume 1000nL, mix move 5mm, manual height: 5 mm

Aspirate 1000 nL from (P3, C7, R2, S1), well volume 0 µL, no over aspirate

Dispense 1000 nL to (P4, C14, R1, S1), well volume 0 µL, mix cycles 3, mix volume 1000nL, mix move 5mm, manual height: 5 mm

Change pipettes

Aspirate 1000 nL from (P3, C8, R1, S1), well volume 0 µL, no over aspirate

Dispense 1000 nL to (P4, C15, R1, S1), well volume 0 µL, mix cycles 3, mix volume 1000nL, mix move 5mm, manual height: 5 mm

Aspirate 1000 nL from (P3, C8, R1, S1), well volume 0 µL, no over aspirate

Dispense 1000 nL to (P4, C15, R1, S1), well volume 0 µL, mix cycles 3, mix volume 1000nL, mix move 5mm, manual height: 5 mm

Change pipettes

Aspirate 1000 nL from (P3, C8, R2, S1), well volume 0 µL, no over aspirate

Dispense 1000 nL to (P4, C16, R1, S1), well volume 0 µL, mix cycles 3, mix volume 1000nL, mix move 5mm, manual height: 5 mm

Aspirate 1000 nL from (P3, C8, R2, S1), well volume 0 µL, no over aspirate

Dispense 1000 nL to (P4, C16, R1, S1), well volume 0 µL, mix cycles 3, mix volume 1000nL, mix move 5mm, manual height: 5 mm

Change pipettes

Aspirate 1000 nL from (P3, C9, R1, S1), well volume 0 µL, no over aspirate

Dispense 1000 nL to (P4, C17, R1, S1), well volume 0 µL, mix cycles 3, mix volume 1000nL, mix move 5mm, manual height: 5 mm

Aspirate 1000 nL from (P3, C9, R1, S1), well volume 0 µL, no over aspirate

Dispense 1000 nL to (P4, C17, R1, S1), well volume 0 µL, mix cycles 3, mix volume 1000nL, mix move 5mm, manual height: 5 mm

Change pipettes

Aspirate 1000 nL from (P3, C9, R2, S1), well volume 0 µL, no over aspirate

Dispense 1000 nL to (P4, C18, R1, S1), well volume 0 µL, mix cycles 3, mix volume 1000nL, mix move 5mm, manual height: 5 mm

Aspirate 1000 nL from (P3, C9, R2, S1), well volume 0 µL, no over aspirate

Dispense 1000 nL to (P4, C18, R1, S1), well volume 0 µL, mix cycles 3, mix volume 1000nL, mix move 5mm, manual height: 5 mm

Change pipettes

Aspirate 1000 nL from (P3, C10, R1, S1), well volume 0 µL, no over aspirate

Dispense 1000 nL to (P4, C19, R1, S1), well volume 0 µL, mix cycles 3, mix volume 1000nL, mix move 5mm, manual height: 5 mm

Aspirate 1000 nL from (P3, C10, R1, S1), well volume 0 µL, no over aspirate

Dispense 1000 nL to (P4, C19, R1, S1), well volume 0 µL, mix cycles 3, mix volume 1000nL, mix move 5mm, manual height: 5 mm

Change pipettes

Aspirate 1000 nL from (P3, C10, R2, S1), well volume 0 µL, no over aspirate

Dispense 1000 nL to (P4, C20, R1, S1), well volume 0 µL, mix cycles 3, mix volume 1000nL, mix move 5mm, manual height: 5 mm

Aspirate 1000 nL from (P3, C10, R2, S1), well volume 0 µL, no over aspirate

Dispense 1000 nL to (P4, C20, R1, S1), well volume 0 µL, mix cycles 3, mix volume 1000nL, mix move 5mm, manual height: 5 mm

Change pipettes

Aspirate 1000 nL from (P3, C11, R1, S1), well volume 0 µL, no over aspirate

Dispense 1000 nL to (P4, C21, R1, S1), well volume 0 µL, mix cycles 3, mix volume 1000nL, mix move 5mm, manual height: 5 mm

Aspirate 1000 nL from (P3, C11, R1, S1), well volume 0 µL, no over aspirate

Dispense 1000 nL to (P4, C21, R1, S1), well volume 0 µL, mix cycles 3, mix volume 1000nL, mix move 5mm, manual height: 5 mm

Change pipettes

Aspirate 1000 nL from (P3, C11, R2, S1), well volume 0 µL, no over aspirate

Dispense 1000 nL to (P4, C22, R1, S1), well volume 0 µL, mix cycles 3, mix volume 1000nL, mix move 5mm, manual height: 5 mm

Aspirate 1000 nL from (P3, C11, R2, S1), well volume 0 µL, no over aspirate

Dispense 1000 nL to (P4, C22, R1, S1), well volume 0 µL, mix cycles 3, mix volume 1000nL, mix move 5mm, manual height: 5 mm

Change pipettes

Aspirate 1000 nL from (P3, C12, R1, S1), well volume 0 µL, no over aspirate

Dispense 1000 nL to (P4, C23, R1, S1), well volume 0 µL, mix cycles 3, mix volume 1000nL, mix move 5mm, manual height: 5 mm

Aspirate 1000 nL from (P3, C12, R1, S1), well volume 0 µL, no over aspirate

Dispense 1000 nL to (P4, C23, R1, S1), well volume 0 µL, mix cycles 3, mix volume 1000nL, mix move 5mm, manual height: 5 mm

Change pipettes

Aspirate 1000 nL from (P3, C12, R2, S1), well volume 0 µL, no over aspirate

Dispense 1000 nL to (P4, C24, R1, S1), well volume 0 µL, mix cycles 3, mix volume 1000nL, mix move 5mm, manual height: 5 mm

Aspirate 1000 nL from (P3, C12, R2, S1), well volume 0 µL, no over aspirate

Dispense 1000 nL to (P4, C24, R1, S1), well volume 0 µL, mix cycles 3, mix volume 1000nL, mix move 5mm, manual height: 5 mm

Change pipettes

References

[i] J. B., Onuska N. P. R. & Nicewicz D. A. Generation and alkylation of α-carbamyl radicals *via* organic photoredox catalysis. *J. Am. Chem. Soc.* **140**, 9056−9060 (2018).
[ii] Proctor, R. S. J., Davis, H., Phipps, R. J. *Science* Catalytic enantioselective Minisci-type addition to heteroarenes. *Science* (2018) DOI: 10.1126/science.aar6376.
[iii] Jin, J. & MacMillan, D. W. C. Direct α-arylation of ethers through the combination of photoredox-mediated C–H functionalization and the Minisci reaction. *Angew. Chem. Int. Ed.* **54,** 1565–1569 (2015).
